# Revision of the fungus-farming ant genus *Sericomyrmex* Mayr (Hymenoptera, Formicidae, Myrmicinae)

**DOI:** 10.3897/zookeys.670.11839

**Published:** 2017-04-24

**Authors:** Ana Ješovnik, Ted R. Schultz

**Affiliations:** 1 Department of Entomology, National Museum of Natural History, Smithsonian Institution, Washington, District of Columbia, United States of America; 2 Maryland Center for Systematic Entomology, Department of Entomology, University of Maryland, College Park, Maryland, United States of America

**Keywords:** Attini, Neotropics, taxonomy, rapid radiation, ultraconserved elements, systematics

## Abstract

The genus *Sericomyrmex* Mayr (Formicidae: Myrmicinae: Attini) is a Neotropical group of fungus-farming ants known for its problematic taxonomy, caused by low morphological variability across the species, vague and old species descriptions, and an outdated and incomplete key published in 1916. Recent molecular studies revealed that *Sericomyrmex* is the product of a rapid recent radiation, with a divergence date of 4.3 million years ago. Here we present a comprehensive taxonomic revision of the genus *Sericomyrmex* based on morphology and a recently published molecular phylogeny. We discuss and illustrate morphological characters for *Sericomyrmex* workers, males, queens, and larvae. We report 18 standard morphological measurements and 5 indices for 529 workers, 50 queens, and 39 males, which we employ in morphometric analyses. The revised genus *Sericomyrmex* comprises eleven species, including three new species, here described as *S. maravalhas* **sp. n.**, *S. radioheadi* **sp. n.**, and *S. saramama* **sp. n.** We also redescribe *S. amabilis* Wheeler, *S. bondari* Borgmeier, *S. lutzi* Wheeler, *S. mayri* Forel, *S. opacus* Mayr, *S. parvulus* Forel, *S. saussurei* Emery, and *S. scrobifer* Forel. The number of recognized species (11) is lower than the previously recognized 19 species and 3 subspecies. The following species and subspecies are synonymized: under *S. opacus* [=*S. aztecus* Forel **syn. n.**, *S. zacapanus* Wheeler **syn. n.**, and *S. diego* Forel **syn. n.**]; under *S. bondari* [=S. *beniensis* Weber **syn. n.**]; under *S. mayri* [=*S. luederwaldti* Santschi **syn. n.**, *S. moreirai* Santschi **syn. n.**, *S. harekulli* Weber **syn. n.**, *S. harekulli arawakensis* Weber **syn. n.**, *S. urichi* Forel **syn. n.**]; under *S. saussurei* [=*S. burchelli* Forel **syn. n.**, *S. impexus* Wheeler **syn. n.**, *S. urichi maracas* Weber **syn. n.**]; and under *S. parvulus* [=*S. myersi* Weber **syn. n.**]. We provide a key to *Sericomyrmex* species for the worker caste and information on the geographic distributions of all species.

## Introduction

The ant genus *Sericomyrmex* Mayr belongs to the fungus-farming ants (Formicidae: Myrmicinae: Attini: subtribe Attina; hereafter “attine” ants), a New World clade of over 250 species (Bolton 2014, Ward et al. 2015), all of which cultivate fungus gardens for food (Mehdiabadi and Schultz 2010). The ants provide the fungus with the substrate on which it grows, either organic detritus or fresh vegetation, thus effectively practicing agriculture (Schultz et al. 2005). *Sericomyrmex* belongs to the so-called “higher”-attine ants, a clade of fungus-farming ants that farms highly specialized, higher fungal cultivars, which are obligate symbionts of ants and never found outside ant nests (Schultz and Brady 2008, Ješovnik et al. 2017). The higher-attine ants include five other genera: the paraphyletic *Trachymyrmex* Forel, the leaf-cutting *Atta* Fabricius and *Acromyrmex* Mayr, a monotypic social parasite *Pseudoatta* Gallardo, and a sister lineage to *Sericomyrmex* with a single known representative, the species *Mycetosoritis explicatus* Kempf. The position of *Sericomyrmex* in the attine-ant phylogeny and its close relatedness to leaf-cutting ants makes the study of *Sericomyrmex* species important for understanding the evolutionary origins of higher-attine ant agriculture and the factors that influenced the immense ecological success of leaf-cutter ants.

**Figure 1.**
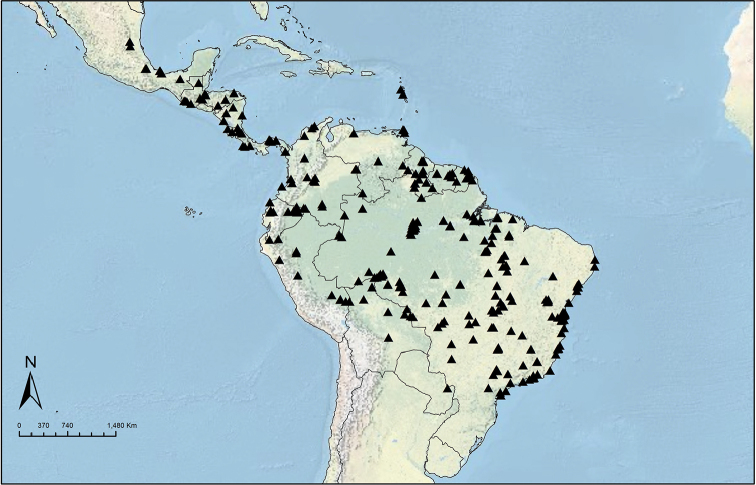
Distribution map of *Sericomyrmex*. Localities of specimens examined for this study.

Like the majority of attine-ant genera, *Sericomyrmex* has a wide Neotropical distribution (Figure 1), ranging from Mexico (Sánchez-Peña 2010) southward to Bolivia, Paraguay, and Paraná, Brazil (Kempf 1972, Brandão 1991, Mayhé-Nunes and Jaffé 1998, Fernández and Sendoya 2004). *Sericomyrmex* species can be found in a variety of habitats, from dry savanna to tropical wet forest, as well as in disturbed, open, and urban habitats (Mehdiabadi and Schultz 2010). Mayr described the genus *Sericomyrmex* and its first species, *Sericomyrmex opacus* Mayr, based on specimens collected by the American naturalist E. Norton in Córdoba, Mexico (Mayr 1865). Since then, a total of 19 species and 3 subspecies have been described, mostly during the early 20^th^ century. The most recent description, of *S. beniensis* Weber from Bolivia, was published in 1938. Forel (1912) published the first key to *Sericomyrmex*, but it contained only the nine species known at that time. Wheeler (1916) modified that key a few years later, adding the newly described *S. lutzi* Wheeler, and since then no keys have been attempted.

The genus *Sericomyrmex* is considered taxonomically difficult mostly because of its substantial morphological homogeneity, further complicated by considerable variation within species and sometimes within colonies, because some species are mildly polymorphic. The differences between some species (e.g., between *S. parvulus* Forel and *S. opacus*) can be very subtle, lacking discrete character states, and not much greater than the differences sometimes observed between two workers of the same species. The original species descriptions tend to be short and are often based on just a few collected foragers, both common practices at the time in which they were written, instead of on complete nest series taken from several geographical locations. As a consequence, some of the characters cited in those descriptions are not useful for distinguishing between *Sericomyrmex* species, since they are now known to vary within species. Thus, even though *Sericomyrmex* ants are commonly collected by standard arthropod collecting methods (e.g., Winkler sifting and pitfall traps), most present-day biologists cannot identify them to species level, and even sorting to morphospecies can be difficult. Previous ant taxonomists were in fact aware of this problem, e.g., Wheeler, in his 1925 paper, writes: “… it now appears that there are several forms … which are so closely related that they may be merely geographical races, or subspecies of one or few highly variable species” (Wheeler 1925b). A moderate degree of within-colony polymorphism, as well as other natural history traits, are shared by *Sericomyrmex* and *Trachymyrmex* species. This separates them from both the monomorphic lower-attine ants, which have smaller colonies and collect insect frass and organic debris, and from the highly polymorphic leaf-cutters, which have enormous colonies and forage for fresh vegetation (Leal et al. 2011).

## Short review of natural history

*Sericomyrmex* ants are light yellow to deep ferrugineous brown and densely covered with long, flexuous hairs, which, to the naked eye, give them a silky, velvety appearance and which earned them their name: “*sericeus*” means “silky” in Latin. When encountered in nature they move slowly and when disturbed they react like most other attine ants: by curling into a ball and becoming immobile. Because of their slow movements and opaque integuments, they are difficult to notice on the forest floor. The nest entrances of some species can be recognized by their raised cylindrical craters consisting of excavated soil particles, but those of other species often consist of just a simple hole in the ground, difficult to notice on the forest floor (Urich 1895, Forel 1912, Wheeler 1925b). *Sericomyrmex* species nest underground and the nest consists of a minimum of one but usually of at least a few subspherical to subelliptical chambers connected by narrow tunnels (Urich 1895, Forel 1912, Weber 1969, Weber 1972, Sosa-Calvo et al. 2015). Each chamber is filled with a fungus garden, a spongy, yellow-brown or yellow-grey mass of mycelium and substrate containing eggs, brood, and workers. The fungus garden can be sessile on the chamber floor (Leal et al. 2011) but is more often suspended from the roof of the chamber by small rootlets (Weber 1972, Fernández-Marín et al. 2004, Mehdiabadi and Schultz 2010). A study in Panamá found that 40 out of 44 colonies of *S. amabilis* had a single foundress queen and that the remaining nests had two to four queens (Fernández-Marín et al. 2004), while a study of Brazilian cerrado species found one to two queens in colonies of *S. scrobifer* (Leal et al. 2011). Colony sizes vary from medium (several hundred workers) to large (~6000 workers), but *Sericomyrmex* colonies never become as large as the colonies of *Atta* leaf-cutter ants (Weber 1972). *Sericomyrmex* workers mostly forage alone, but in species with larger colonies (e.g., *S. mayri* Forel and *S. bondari* Borgmeier) they sometimes form short, dense foraging columns in the immediate area of the nest entrance. They collect organic material, mostly freshly fallen leaflets, sometimes moss, grass, leaves, and fruits (Leal and Oliveira 1998, De Fine Licht and Boomsma 2010). They can also feed on seeds but will usually not bring them back to the nest (Feldmann et al. 2000). They cut leaves on some occasions (Weber 1967, 1972) but do not climb vegetation in order to cut leaves as leaf-cutter ants do (Leal et al. 2011). Local occurrences of *Sericomyrmex* are often patchy, with colonies abundant in some areas but absent or sparse in adjacent areas. In areas where they are abundant they can be very dense, with nest entrances very close to each other. For example, during an attine-ant study in Trinidad and Tobago, 67 *Sericomyrmex* nest entrances were observed in an 18 x 77-meter area in one year (Weber 1972). The species *Sericomyrmex amabilis* Wheeler is the host to the socially parasitic ant *Megalomyrmex symmetochus* Wheeler, which specializes on *Sericomyrmex* species (Wheeler 1925b, Adams et al. 2013) and which provides protection to the host-ant colony by defending it from the specialized ant agro-predator *Gnamptogenys hartmani* Wheeler (Adams et al. 2013).

Here we present the first comprehensive taxonomic revision of the ant genus *Sericomyrmex*, recognizing 11 species. Eleven species and two subspecies are synonymized and three new species (*maravalhas*, *saramama*, *radioheadi*) are described. In addition to morphological characters of workers, queens, males, and larvae, this revision was strongly informed by molecular data. A well-supported phylogeny of 88 *Sericomyrmex* individuals and 5 outgroup taxa (including *Trachymyrmex iheringi* group species and *Mycetosoritis explicatus*), based on genomic data from ultraconserved elements (UCE), informed our decisions about species delimitation (Ješovnik et al. 2017).

## Materials and methods

### Morphological analyses

The majority of specimens examined for this study were collected during extensive field work in South and Central America by members of the Smithsonian Ant Lab, mainly Ted R. Schultz, Jeffrey Sosa-Calvo, and Ana Ješovnik. In all, we obtained a total of ~19,000 specimens from 17 countries for this study, including 19 complete *Sericomyrmex* nests that accounted for >17,550 individuals (even though each of those individuals was counted, not all were examined). Collected nests represent seven different species: *S. amabilis* (2 nests), *S. bondari* (3 nests), *S. saramama* (1 nest), *S. mayri* (8 nests), *S. opacus* (2 nests), *S. parvulus* (3 nests), and *S. saussurei* (1 nest). The total number of pinned specimens examined was >1,400. The collection localities are indicated in Figure 1 and the collection and specimen data for all specimens examined are listed in Suppl. material 2: Table S2f. All data, including all images, are available on AntWeb (http://www.antweb.org) (California Academy of Sciences).

We examined and measured adult ant specimens with an MZ16 Leica stereomicroscope with an ocular micrometer. We recorded measurements to the nearest 0.001 mm, but we conservatively report them to an accuracy of two decimal places. A minimum of 25 workers were measured for each species, except for *S. lutzi* and *S. radioheadi* Ješovnik & Schultz, for which only eight and nine specimens were available, respectively. We chose measured workers to represent the geographic range of the species and to estimate within-colony variation, with up to 12 workers from the same nest measured when nest series were available.

We photographed workers, queens, and males using a JVC KY-F70B video camera mounted on an M420 Leica stereomicroscope. The images were assembled using Automontage Pro version 5.03.0018 software. We prepared queen and male wings on microscope slides with Euparal mounting medium. To prepare larvae we dehydrated them in 100% absolute ethanol, critical-point dried in liquid CO_2_ in Balzers CPD-030, and coated with gold and palladium alloy in a Cressington Scientific 108 Auto sputter-coater to a thickness of 20–25 nm. We then imaged the coated larval specimens with a Philips XL–30 ESEM Scanning Electron Microscope (SEM) in the SEM Lab in the National Museum of Natural History in Washington, D.C. Adult worker, queen, and male specimens and dissected male genitalia were air-dried for a few minutes, then coated with gold to a thickness of 30–70 nm using a Cressington Scientific 108 Auto sputter-coater. We took SEM images of the workers, queens, and males with a Hitachi TM3000 Tabletop SEM. We used Adobe Photoshop CC to edit and enhance images and prepare all figures following the ant-image editing instructions on AntWeb (http://www.antweb.org).

All latitudes and longitudes are provided in decimal degrees and elevations in metric units. For specimens for which GPS coordinates were unavailable, we estimated the longitude and latitude based on locality data using AntWeb (http://www.antweb.org), GEONet Names Server (http://geonames.nga.mil/gns/html/index.html), Google Earth (http://www.google.com/ earth/index.html), and/or Google Maps (https://www.google.com/maps). Estimated coordinates are indicated by square brackets. Date of collection is in the day/month/year format, with the month spelled with the first three letters to avoid uncertainty. Data for type material follows the format: [Country], [First administrative district], [Locality], [GPS coordinates], [Elevation], [Collection code], [Collector], [Collection date], [Habitat], and for each of the type specimens examined: (Repository: number and caste of specimens, specimen code). In cases where no holotype exists, we designate one of the specimens as the lectotype and the rest of the syntype specimens as paralectotypes. When there is more than one ant on the pin, the position on the pin of the lectotype is indicated (e.g., “topmost specimen on the pin”). Data for the material examined is not an exhaustive list of every specimen seen, but instead provides an overview of the geographic range of the examined specimens. The data for material examined is organized alphabetically by country, first administrative district, and then in the following format: [Locality], [GPS coordinates], [Elevation], [Collection date], [Collector].

The collections visited, from which material was borrowed and/or into which material was deposited, are referred to by the following acronyms (Brandão 2000):

**AMNH** American Museum of Natural History, New York, New York, USA.

**BMNH** The Natural History Museum, London, U.K.

**CASC** California Academy of Sciences, San Francisco, California, USA.

**CEPEC** Laboratório de Mirmecologia Itabuna, Bahia, Brazil.

**DZUP** Museu de Entomologia Pe. Jesus Santiago Moure, Universidade Federal do Paraná, Curitiba, Paraná, Brazil.

**IAVH** Instituto de Investigación de Recursos Biológicos Alexander von Humboldt, Villa de Leyva, Colombia.

**ICN** Insect Collection, Instituto de Ciencias Naturales, Universidad Nacional de Colombia, Bogotá D.C., Colombia.

**INPA** Instituto Nacional de Pesquisas da Amazônia, Manaus, Amazonas, Brazil.

**MBC–UFU** Museu de Biodiversidade do Cerrado, Universidade Federal de Uberlândia, Uberlândia, Minas Gerais, Brazil.

**MCZ** Museum of Comparative Zoology, Harvard University, Cambridge, Massachusetts, USA.

**MHNG** Museum d’Histoire Naturelle, Genève, Switzerland.

**MHNL** Museo de Historia Natural, Lima, Peru.

**MPEG** Museu Paraense Emílio Goeldi, Belém, Brazil.

**MPUJ** Museo Javeriano de Historia Natural “Lorezo Uribe”, S.J. Pontifcia Universidad Javeriana, Bogotá D.C., Colombia.

**MSNG** Museo Civico di Storia Naturale “Giacomo Doria”, Genoa, Italy.

**MZSP** Museu de Zoologia da Universidade de São Paulo, São Paulo, Brazil.

**NHMB** Naturhistorisches Museum, Basel, Switzerland.

**NHMW** Naturhistorisches Museum Wien, Vienna, Austria.

**PSWC** Philip S. Ward Collection, University of California, Davis, California, USA.

**UFVB** Museu de Entomologia, Universidade Federal de Viçosa, Viçosa, Minas Gerais, Brazil.

**USNM** National Museum of Natural History, Washington, DC, USA.

**UVGC** Colección de Artrópodos, Universidad del Valle de Guatemala, Guatemala City, Guatemala.

### Measurements and indices

Morphological terminology and measurement indices follow Snodgrass (1910), Tulloch (1929), Bolton (1994), Schultz and Meier (1995), Mackay et al. (2004), Rabeling et al. (2007), Sosa-Calvo and Schultz (2010), and Ješovnik et al. (2013). Male genitalia terminology follows Boudinot (2013). Morphological measurements, index abbreviations, and definitions are as follows:

### Measurements

**HWe** Head width: in full-face view, the maximum width of the head including eyes.

**HW** Head width: in full-face view, the maximum width of the head just above the eyes.

**HW1** Head width at the top of head: in full-face view, the maximum width between the points where the frontal carina meets the posterior cephalic corner.

**HW2** Head width posterior: in full-face view, the maximum width of the posterior part of the head.

**HW3** Anterior head width most: in full-face view, the maximum width of most anterior end of head capsule, measured between the points where lateral most edge of the clypeus connects with the head capsule.

**Figure 2.**
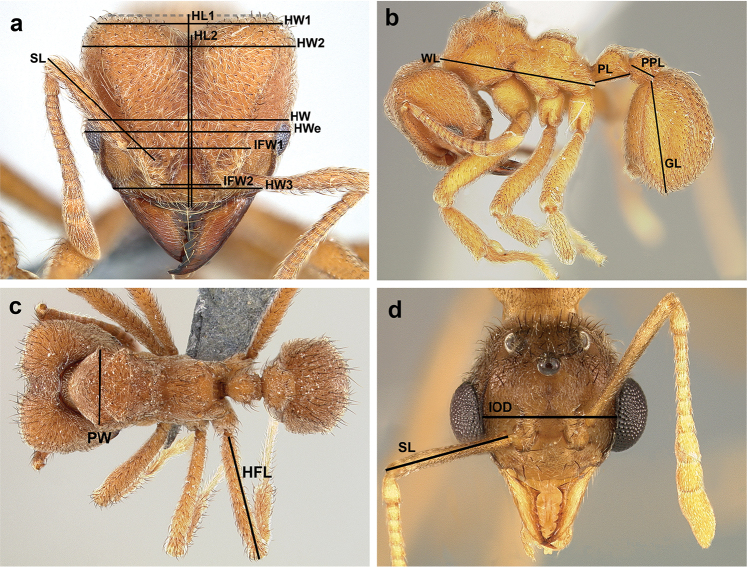
Morphological measurements. Indicating measurements of the worker (**a, b, c**) and male (**d**). **HWe** Head width including the eyes **HW** head width just above the eyes **HW1** head width at the top of the head **HW2** posterior head width **HW3** anterior head width **HL1** head length 1 **HL2** head length 2 **SL** scape length **IFW1** interfrontal width 1 **IFW2** interfrontal width 2 **WL** Weber’s length **PL** petiole length **PPL** postpetiole length **GL** gaster length **PW** pronotal width **HFL** hind femur length **IOD** interocular distance.

**HL1** Head length 1: in full-face view, the maximum length of the head from a line tangential to the posteriormost margins of the head to the line tangential to anteriormost margin of clypeal apron (since both posterior cephalic border and clypeus are medially notched, see Figure 2a).

**HL2** Head length 2: HL1 minus the posterior cephalic emargination.

**SL** Scape length: in full face-view, the maximum length of the scape, measured from the middle of the frontal lobe fenestra (because the frontal lobe conceals the base of the scape) to the distal end of the scape.

Scape length male (measured differently in males because the base of the scape is visible): in full face-view, the maximum length of the scape, excluding the constriction that occurs just distal of the condylar bulb (Figure 2d).

**IFW1** Interfrontal width 1: in full face-view, the maximum distance between the lateralmost points of the frontal lobes, which, in all *Sericomyrmex*, is the point where the lateral and posterior margin of the frontal lobe meet.

**IFW2** Interfrontal width 2: in full face-view, the maximum distance between the interior margins of the frontal lobes, or the distance between the points where the lateral and medial margin of each frontal lobe meet.

**IOD** Interocular distance (male only): in full-face view, the maximum width of the head measured at the midpoint of the internal margin of the eyes (Figure 2d).

**Om** Ommatidia count: in lateral view, number of ommatidia visible in the maximum diameter of the eye.

**WL** Weber’s length: in lateral view, length of the mesosoma from the point where the pronotum curves into the cervical shield to the posteriormost ventral angle of the propodeum.

**HFL** Hind femur length: in most appropriate view (usually the dorsal), length of the hind femur, not including the trochanter.

**PL** Petiole length: in lateral view, the maximum length of the petiole measured from the posteriormost margin of the metapleural gland lobe to the posteriormost margin of the petiole.

**PPL** Postpetiole length: in lateral view, the maximum length of the postpetiole.

**GL** Gaster length: in lateral view, the distance from the anteriormost point of the tergo-sternal articulation of the first gastral (fourth abdominal) segment to the posteriormost tip of the gaster.

**PW** Pronotal width: in dorsal view, the maximum width of the pronotum.

**FWg** Front wing length (queen and male only).

**HWg** Hind wing length (queen and male only).

### Indices

**CI** Cephalic index worker and queen: (HW/HL)×100.

Cephalic index male: (IOD/HL)×100.

**CEI** Cephalic emargination index: ((HL1-HL2)/HW)×100.

**FLI** Frontal lobe index worker and queen: (IFW1/HW)×100.

Frontal lobe index male: (IFW/IOD)×100.

**SI** Scape index worker and queen: (SL/HW)×100.

Scape index male: (SL/IOD)×100.

**OI** Ocular index: (EL/HW)×100.

### Morphometric analysis

The quantitative morphological data set consists of 529 measured worker individuals and 23 variables (18 measurements and 5 indices) (Suppl. material 2: Table S2a). Measurements that we could not take (e.g., due to a damaged specimen or to the position of a specimen on a pin) were scored as “NA” and treated as missing values in the analyses, except for the principal component analysis (PCA), from which individuals with missing values were excluded. We performed all analyses in R (R Development Core Team 2014) using the MorphoTools functions set (Koutecký 2015) and R packages vegan and ade4. The R script we used and the text file with the data are available on GitHub (see Data resources below).

We ran basic descriptive statistics to examine the data visually and to detect possible errors and outliers. We manually rechecked all outliers for all species by remeasuring them. Not all size-related measurements were repeated, however, in cases in which the remeasuring of one variable was sufficient to confirm an outlying large- or small-sized individual. Based on a Shapiro-Wilk normality test run on the entire data set, only two variables, CI (cephalic index) and SI (scape index), had normal distributions, so the data set was log-transformed for PCA analyses. To check for correlations between different variables we calculated Spearman’s correlation coefficient. After removing the correlated parameters (HW, HL1, HL2, WL, HFL) the data set for PCA contained 18 variables. We calculated the PCA of individuals using the functions pca.cor, pca.eigen, and pca.scpres and the correlations of characters (character loadings) with the function pca.cor (Koutecký 2015). The character loadings indicate to what extent each character contributes to each of the principal components and therefore indicate what characters are responsible for most of the variation.

In addition to the full data set, containing all the species, we created reduced data matrices, with only certain taxa or populations included, to further explore morphological separation in just those groups. The species-level reduced data sets included: 1) *S. opacus* and *amabilis*, 2) *S. opacus* and *parvulus*, and 3) *S. scrobifer* Forel and *maravalhas* Ješovnik & Schultz. The population-level reduced data sets included: 1) populations of striate-mandibled vs. smooth-mandibled *S. amabilis*, 2) populations 1, 2, and 3 of *S. opacus*, 3) populations of typical *S. bondari* vs. reduced-hair *S. bondari*, 4) populations of striate-mandibled vs. smooth-mandibled *S. saussurei* Emery, and 5) populations of *S. mayri*. Also, we ran basic descriptive statistical analyses on data sets of queen-only (50 individuals) and male-only (39 individuals) specimens.

## Data resources

The source code for MorphoTools, our customized script, and the complete data set used in the analyses are publicly available at https://github.com/anajesovnik/Sericomyrmex-morphology. Specimen data for all material examined in this study, along with all ant images, are publicly available at AntWeb (http://www.antweb.org).

## Results

### Morphometric analysis

**Figure 3.**
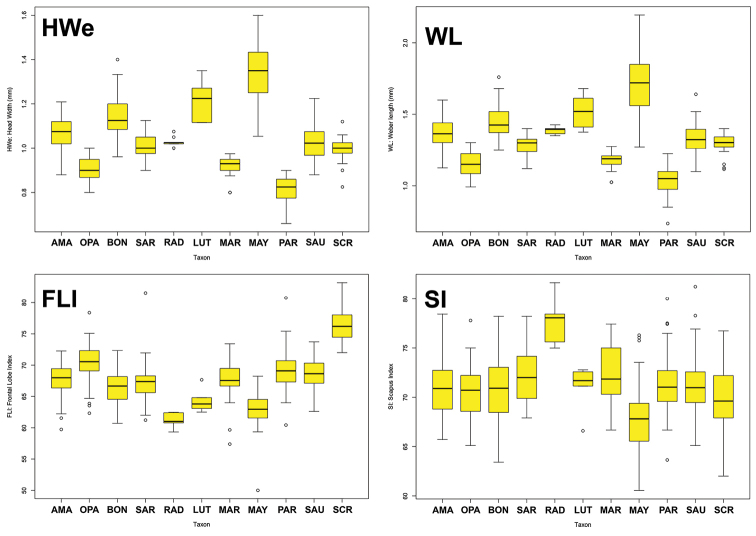
Box plots for morphological variables. **HWe** Head width including the eyes **WL** Weber’s length **FLI** frontal lobe index **SI** scape index. Species name abbreviations: **AMA** *amabilis* **OPA** *opacus* **BON** *bondari* **SAR** *saramama* **LUT** *lutzi* **MAR** *maravalhas* **MAY** *mayri* **PAR** *parvulus* **RAD** *radioheadi* **SAU** *saussurei* **SCR** *scrobifer*.

**Figure 4.**
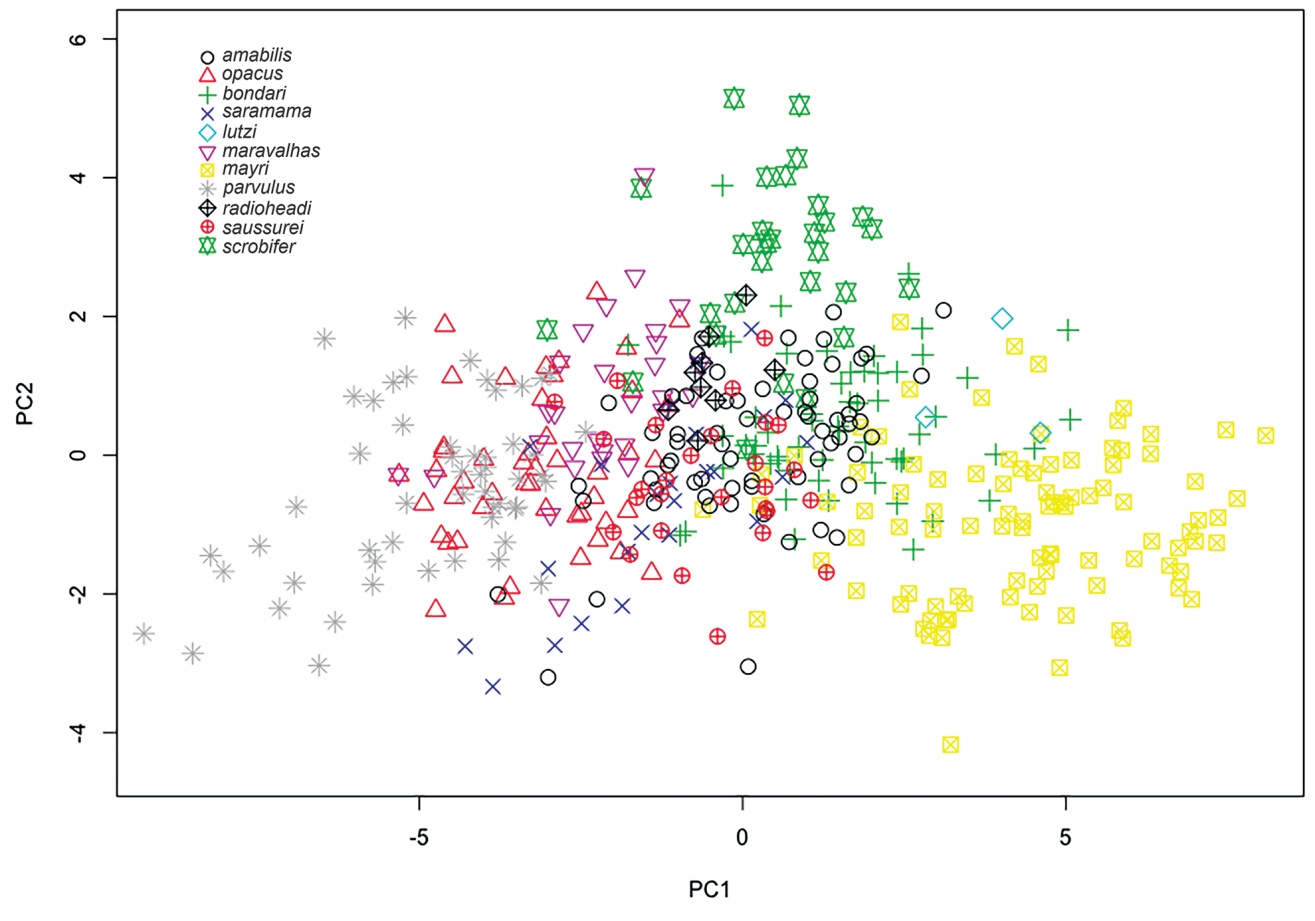
Principal component analysis for all species, scatter plot.

Table 1 summarizes the statistics for each species, with mean and standard deviation (SD) values for chosen variables. Figure 3 contains box plots for chosen variables. The plot in Figure 4 is based on the first two principal components identified by the principal component analysis, which together are responsible for 71.8% of the observed variance. The first principal component is strongly correlated with head width and other measurements of the head (Table 2) and it accounts for 60.8% of the observed variance. The second axis, principal component two, accounts for 11.2% of the observed variance. It is influenced mostly by the ocular index (OI) and the cephalic emargination index (CEI), and somewhat less by the cephalic index (CI) and the frontal lobe index (FLI). We observe that along the *y* axis (PCA2) individuals are separated mainly by the relative sizes of their eyes and by the relative depths of their posterior cephalic emarginations but also to a lesser extent by head shape and relative frontal lobe width. In general PC1 represents size and PC2 represents shape, the latter represented by ratios of certain characters. The third principal component did not add much more resolution when plotted in three dimensions. It is similar to PC2, influenced mostly by CEI and OI, and it accounts for 6.97% of variance. The fourth component (PC4), which accounts for only 5.02 % of variance, is interesting because it is highly correlated with SI, the scape index, with all other variables having much lower variation (Table 2).

**Figure 5.**
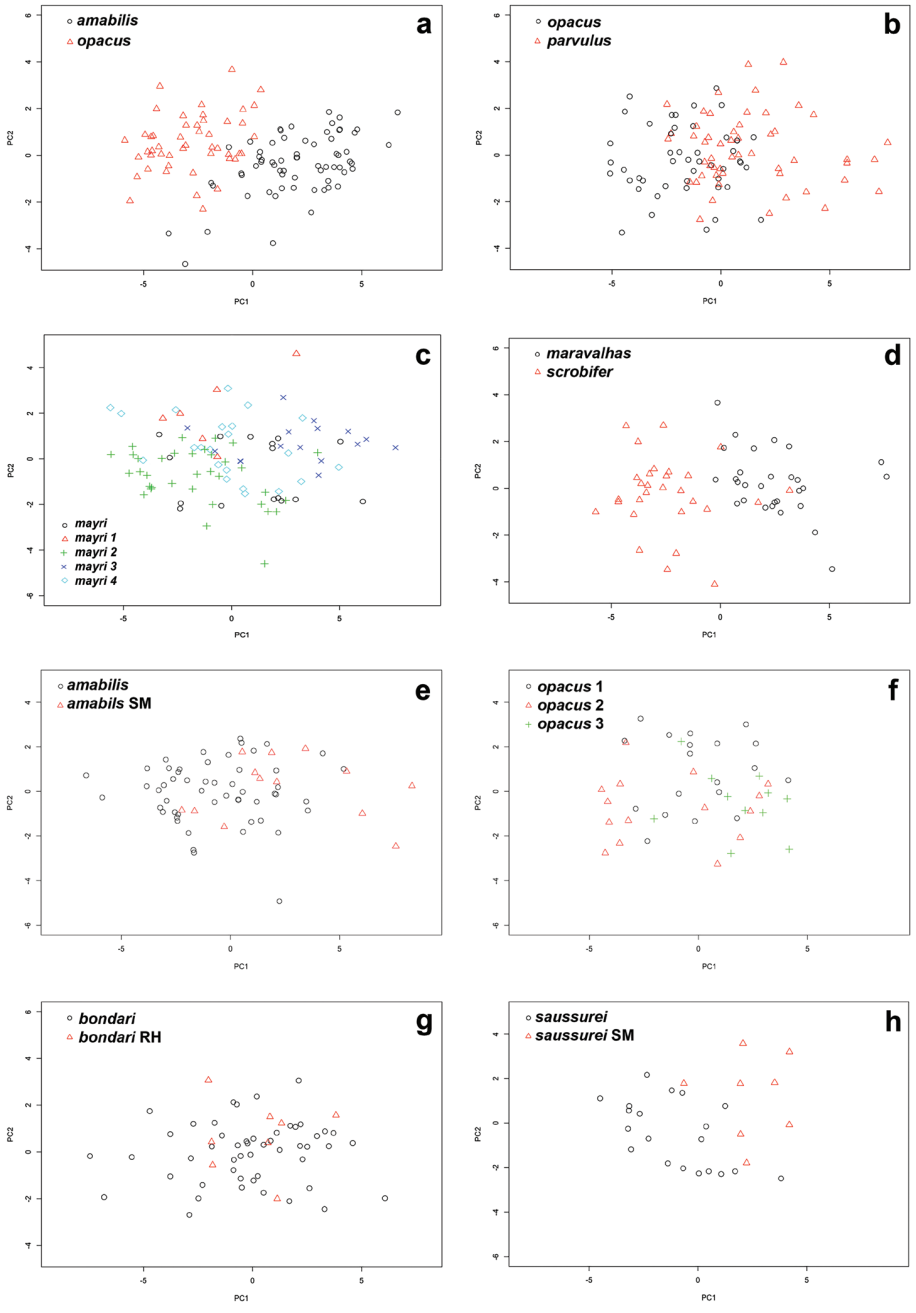
Principal component analysis of reduced data sets, scatter plots. **a** *S. amabilis* and *S. opacus* **b** *S. opacus* and *S. parvulus* **c** populations of *S. mayri* **d** *S. maravalhas* and *S. scrobifer* **e** populations of striate-mandibled *S. amabilis* vs. smooth-mandibled (SM) *S. amabilis* **f** populations of *S. opacus* **g** populations of typical *S. bondari* vs. reduced-hair (RH) *S. bondari* **h** populations striate-mandibled *S. saussurei* vs. smooth-mandibled (SM) *S. saussurei*.

In general, the results of the PCA analyses (Figure 4) are congruent with genetic data for *Sericomyrmex*, which showed high genetic similarity, indicating that all species are very closely related and that speciation in this genus occurred recently (Ješovnik et al., 2017). Species clusters overlap, with a subset of species separated mostly due to size (axis *x*). PCA analyses of the reduced data sets are easier to interpret (Figure 5). These results are discussed in more detail in the notes sections of the corresponding species.

**Table 1.** Morphological measurements. Mean and standard deviation (SD) values for selected morphological measurements and indices for the *Sericomyrmex* species. All measurement values are in millimeters. N represents the number of workers measured for each species. The full statistics for all of the morphological measurements can be found in Suppl. material 2: Table S2e.

**Species (N)**	***amabilis* (70)**		***bondari* (59)**		***lutzi* (6)**		***maravalhas* (30)**	
	mean	SD	mean	SD	mean	SD	mean	SD
HWe	1.06	0.07	1.15	0.09	1.22	0.09	0.92	0.04
IFW1	0.72	0.06	0.76	0.07	0.78	0.07	0.62	0.05
HL1	1.02	0.07	1.10	0.07	1.25	0.08	0.88	0.05
SL	0.75	0.05	0.81	0.05	0.86	0.05	0.66	0.04
WL	1.37	0.10	1.44	0.11	1.52	0.13	1.18	0.06
HFL	1.15	0.09	1.27	0.09	1.29	0.07	0.99	0.06
CI	104	3	104	3	103	2	104	3
FLI	68	2	67	2	64	2	68	3
SI	71	3	71	3	71	2	72	3
CEI	10	2	13	2	15	1	10	2
	***mayri* (103)**		***opacus* (68)**		***parvulus* (55)**		***radioheadi* (9)**	
HWe	1.35	0.13	0.90	0.05	0.81	0.06	1.03	0.02
IFW1	0.85	0.08	0.64	0.05	0.56	0.05	0.63	0.02
HL1	1.25	0.12	0.90	0.04	0.80	0.06	1.02	0.03
SL	0.91	0.07	0.64	0.07	0.58	0.05	0.80	0.02
WL	1.71	0.18	1.15	0.08	1.04	0.10	1.39	0.03
HFL	1.48	0.13	0.92	0.06	0.82	0.08	1.24	0.04
CI	108	3	100	3	102	3	101	2
FLI	63	3	70	3	69	3	61	1
SI	68	3	71	3	71	3	77	2
CEI	10	2	9	3	9	2	14	2
	***saramama* (25)**		***saussurei* (68)**		***scrobifer* (31)**			
HWe	1.01	0.06	1.02	0.08	1.00	0.05		
IFW1	0.68	0.06	0.70	0.07	0.76	0.04		
HL1	0.98	0.07	0.99	0.08	0.97	0.05		
SL	0.72	0.04	0.73	0.06	0.70	0.04		
WL	1.28	0.08	1.33	0.11	1.29	0.07		
HFL	1.10	0.07	1.12	0.12	1.11	0.08		
CI	103	3	104	3	103	3		
FLI	68	4	69	2	76	3		
SI	72	3	71	3	70	4		
CEI	10	2	10	2	12	1		

**Table 2.** Principal component analysis. Character loadings of the first six principal components for all species. The values above 0.6 are indicated in bold font.

Character	PC1	PC2	PC3	PC4	PC5	PC6
HWe	**0.98**	-0.14	0.03	0.04	0.00	-0.04
IFW1	**0.96**	0.07	-0.07	-0.11	0.19	-0.11
IFW2	**0.87**	-0.06	-0.02	0.08	0.18	0.02
SL	**0.95**	-0.05	0.11	0.25	0.08	-0.11
EL	**0.80**	0.37	-0.37	0.16	-0.21	0.04
Om	**0.77**	0.30	-0.26	0.10	-0.18	-0.14
PL	**0.79**	0.04	-0.03	0.00	0.18	0.39
PPL	**0.80**	0.13	0.00	0.07	0.17	0.26
GL	**0.94**	-0.06	-0.05	0.03	0.07	-0.02
PW	**0.96**	-0.06	-0.01	0.02	0.05	0.01
HW3	**0.95**	-0.13	0.02	0.08	0.03	0.01
CI	0.54	-0.54	-0.29	-0.06	-0.31	-0.26
FLI	-0.38	0.58	-0.27	-0.40	0.49	-0.17
SI	-0.45	0.35	0.23	**0.68**	0.29	-0.22
OI	-0.33	**0.69**	-0.54	0.16	-0.27	0.11
FLD	**0.88**	0.15	-0.10	-0.24	0.16	-0.18
CEI	0.37	**0.62**	0.57	-0.20	-0.30	-0.03

### Species delimitation

In delimiting species, we adopted the modified biological species concept (BSC) (Mayr 1942) in the sense of Coyne and Orr (2004), which defines species as groups of interbreeding natural populations that are characterized by substantial but not necessarily complete reproductive isolation, thus not excluding some degree of introgression after evolutionary divergence. Unlike phylogenetic species concepts (De Queiroz and Donoghue 1988, Cracraft 1989, Baum and Donoghue 1995), which require sister species to be reciprocally monophyletic, the BSC allows for the reality that gene phylogenies may reconstruct sister species as non-monophyletic with respect to one another because of incomplete lineage sorting (Brown et al. 1994, Funk et al. 1995, Harrison 1998, Coyne and Orr 2004). Given the relatively recent origins of *Sericomyrmex* species (Ješovnik et al. 2017), such an allowance is critical. In fact, we encountered one such case, where *S. amabilis* is rendered paraphyletic by *S. saussurei* (see *S. amabilis* notes section for details).

Our practical criteria for recognizing species were (i) a well-supported sister-group relationship (not necessarily reciprocally monophyletic) in our molecular phylogeny (Suppl. material 1) and (ii) a unique combination of morphological character states, including characters of workers, queens, males, and larvae, as well as measurement characters based on our morphometric analyses of workers. In recognizing species we also took into account the geographic distributions of populations. E.g., we did not describe two molecularly distinct allopatric populations as separate species if they were morphologically indistinguishable, whereas we described co-occurring, genetically and/or morphologically distinct, sympatric populations as two separate species.

### Sericomyrmex genus description

Following are genus-level descriptions and diagnoses of *Sericomyrmex* workers, queens, males, and larvae, describing character states that are shared by all species within the genus. In the character discussion section below we discuss characters with states that are variable across the genus but fixed within species, and therefore important for distinguishing between *Sericomyrmex* species.

### *Sericomyrmex* worker

**Figure 6.**
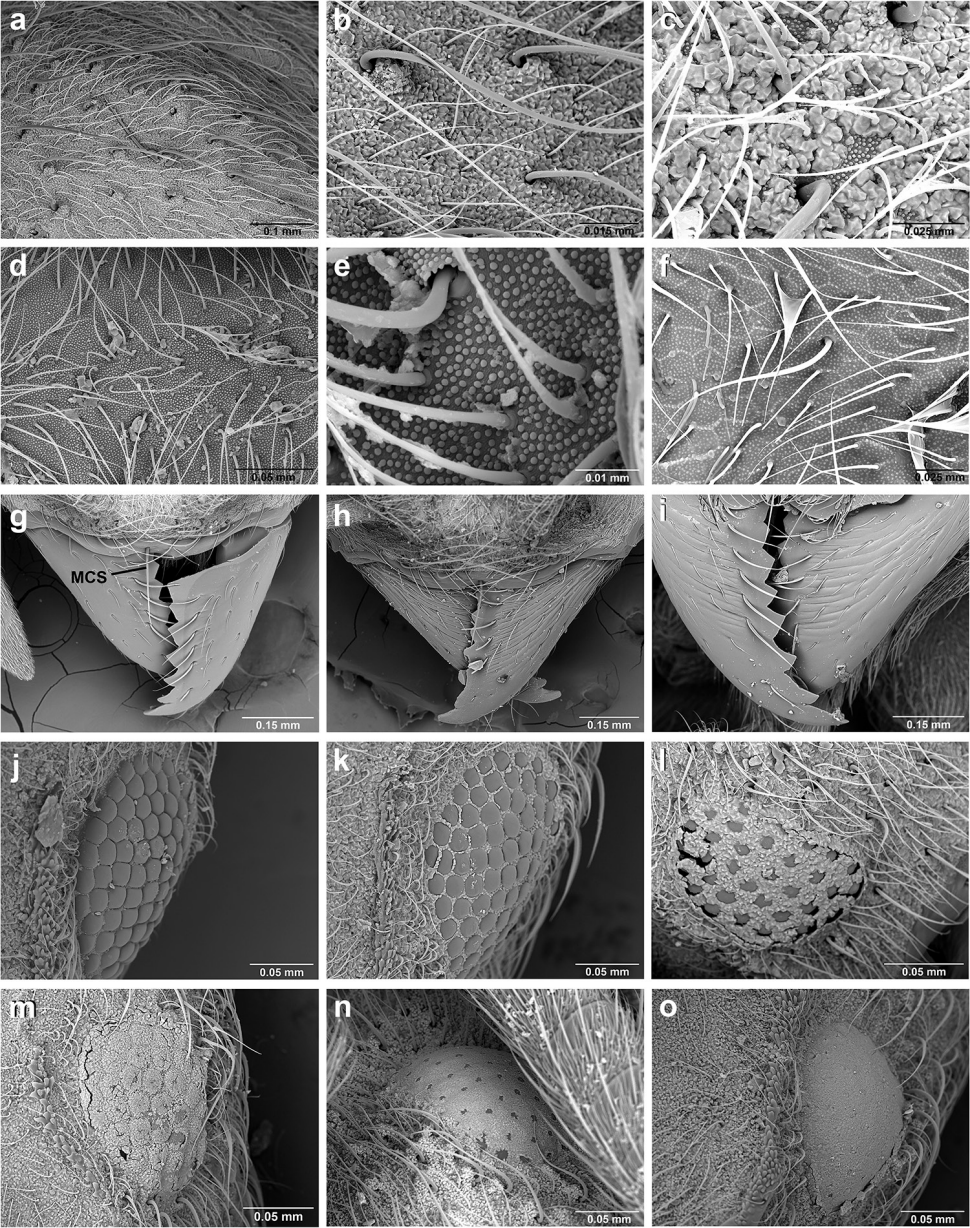
*Sericomyrmex* morphology, SEM images: integument, mandibles, and eyes. Integument: **a**, **b, c** adult worker integument **d**, **e** callow worker integument; **f** male integument. Mandibles: **g** smooth (*S. parvulus* worker) **MCS** median clypeal seta **h** striate (*S. saussurei* worker) **i** faintly striate (*S. mayri* callow worker). Eyes: **j, k** eyes without white layer; **l** partially covered eye (*S. parvulus*) **m** completely covered eye, but individual ommatidia still discernible (*S. opacus*) **n** eye with thick white layer, ommatidia visible through narrow holes (*S. saussurei*) **o** eye completely covered with white layer, individual ommatidia not visible (*S. saussurei*).

**General appearance.** Small to medium-sized (mean WL: *parvulus*=1.04 mm, *mayri*=1.71 mm), body color evenly light yellow or yellowish-brown to deep, ferrugineous-brown. Some individuals with darker areas on frontal lobe and along frontal carina. Integument dull and opaque, in adult workers covered with apparent waxy, crystal-shaped cuticular layer (Figure 6a–c), absent in callow workers and males (Figure 6d–f), and completely or partially absent in some workers of *S. maravalhas* (see *maravalhas* notes for details).

Entire body covered with dense pubescence: short, thin, appressed to decumbent, light yellow hairs. Entire body also with thicker (Figure 6b–c), longer, often flexuous hairs, yellow to gray or black, darker in color at base, appressed to erect. Dorsum of head, mesosoma, and metasoma with hair denser and longer than remaining body. Mandible and metapleural gland bulla devoid of hairs, glossy.

**Figure 7.**
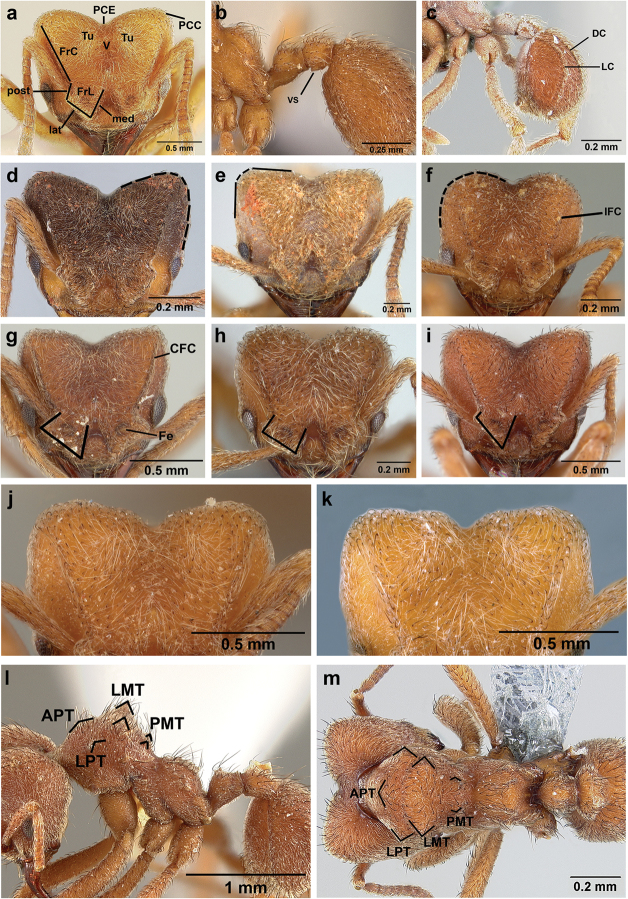
*Sericomyrmex* worker morphology and terminology. **a** Head, full-face view **b** petiole and postpetiole, lateral view; **c** metasoma, lateral view. Posterior cephalic corner shapes: **d** acute **e** rectangular **f** rounded. Frontal lobe shapes: **g** trapeziform **h** rectangular **i** triangular. Posterior cephalic border shapes: **j** posterior cephalic border gradually impressed **k** posterior cephalic border abruptly impressed. Mesosoma morphology: **l** mesosoma, lateral view **m** mesosoma, dorsal view. **PCC** Posterior cephalic corner **PCE** posterior cephalic emargination **Tu** tumulus **V** vertexal impression **FrC** frontal carina **FrL** frontal lobe **post** posterior frontal lobe margin **lat** lateral frontal lobe margin **med** medial frontal lobe margin **vs** postpetiolar ventral sternite **LC** longitudinal lateral gastral carina **DC** anteromedial dorsal gastral carina **IFC** incomplete frontal carina **CFC** complete frontal carina **Fe** frontal lobe fenestra **APT** anterior pronotal tumulus **LPT** lateral pronotal tubercle **LMT** lateral mesonotal tubercle **PMT** posterior mesonotal tubercle.

**Head.** In full-face view cordate, tapering anteriorly, lateral margin slightly convex, posterior cephalic corner acute to rounded, never with tubercles or spines. Posterior cephalic margin medially emarginate. Vertex medially impressed in full-face view (Figure 7a: V), low tumuli (Figure 7a: Tu) laterad of vertexal impression, one on each side, sometimes distinct and sometimes barely visible. Anterior clypeal border broadly convex. Clypeal apron with shallow median notch, median clypeal seta arising from its middle (Figure 6g: MCS). Mandible triangular, with 7–9 teeth, dorsally glossy, variably striate or smooth. Lateral margin of mandible in full-face view straight in basal two-thirds, curving at apex. Edge of masticatory margin with 2–6 short, decumbent hairs, directed medially. Slender, light-colored, short hairs evenly but sparsely distributed over entire mandibular dorsum, directed apically (Figure 6g–i). Palp formula 4, 2. Preocular carina directed posterad, fading posterior to eye, never reaching posterior cephalic corner. Eye distinct, placed laterally on anterior half of head, variable in size and shape, from almost flat to convex and protruding laterally, in some species covered with silvery-white layer of unknown, waxy substance, hereafter referred to as “white layer.” Number of ommatidia across largest eye diameter 7–14. Frontal lobe always completely covering antennal condylar bulb; with three distinct margins: posterior, lateral, and medial (Figure 7a); and with dorsal fenestra, i.e., thinner, sometimes almost translucent area just above condylar bulb, circular in shape and darker in color on edges (Figure 7g: Fe). Lobe size and shape vary. Frontal carina straight to slightly curved laterally, diverging toward posterior cephalic corner, in some species reaching cephalic corner, in others not. Area laterad of frontal carina (incomplete antennal scrobe) with less hair than rest of head, mostly appressed pubescence. Antenna 11-segmented, lacking distinct antennal club. Antennal scape relatively short, in most species not reaching posterior cephalic corner, slightly narrower basally, angled in first third, slightly curved distally. Antennal pedicel longer than funicular segments two and three combined.

**Mesosoma** (Figure 7l–m). Anteroventral pronotal corner obtuse, sometimes bearing small, anterad-directed denticle, best seen in lateral view. Pronotum anteriorly with single low median tumulus and two larger lateral pronotal tubercles, both best seen in frontodorsal view. Mesonotum with two lateral mesonotal tubercles (most prominent feature on mesosoma) and two smaller, lower, posterior mesonotal tubercles. Metanotal groove distinct, best seen in lateral view, with reduced hairs. Propodeum dorsally with two longitudinal, low, posteriorly diverging carinae, sometimes weakly serrate, each often with small and blunt posterodorsal denticle. Propodeal spiracular carinae absent, propodeal spiracle opening oval, directed posterad, mounted on small tubercle. Metapleural gland bulla transparent, glossy, devoid of hairs. Mesotibial and metatibial spurs absent. Arolium present.

**Metasoma.** Petiole with short peduncle, in lateral view longer than postpetiole, lacking subpetiolar process. Petiole and postpetiole each with pair of low, short, sometimes serrate, longitudinal dorsal carinae, sometimes reduced to low denticles, best seen in dorsolateral view. Postpetiole in some species with another pair of carinae laterally, sometimes reduced to low denticles. Postpetiole in dorsal view broader than long and broader than petiole. Ventral sternite of postpetiole protruding anteriorly, sometimes forming lobe in lateral view (Figure 7b). First gastral tergite (A4) larger and longer than first sternite, dorsally overhanging remaining segments (A5–A8). First gastral tergite laterally impressed on both sides, with weakly to strongly developed lateral longitudinal carinae along anterior two-thirds, sometimes also with weakly to strongly developed anteromedian dorsal carinae (Figure 7c).

### *Sericomyrmex* queen

(Genus-level description of *Sericomyrmex* queen based on *S. amabilis*, *S. bondari*, *S. lutzi*, *S. maravalhas*, *S. mayri*, *S. opacus*, *S. parvulus*, *S. saramama*, and *S. saussurei*. Queens of *S. radioheadi* and *S. scrobifer* unknown.)

**General appearance.** Larger than worker (e.g., *amabilis* worker and queen, mean values: HW=1.06 w, 1.32 q; WL=1.37 w, 2.05 q; GL=0.96 w, 1.77 q), color ferrugineous-brown, often darker than worker. Pilosity as in worker or somewhat denser.

**Figure 8.**
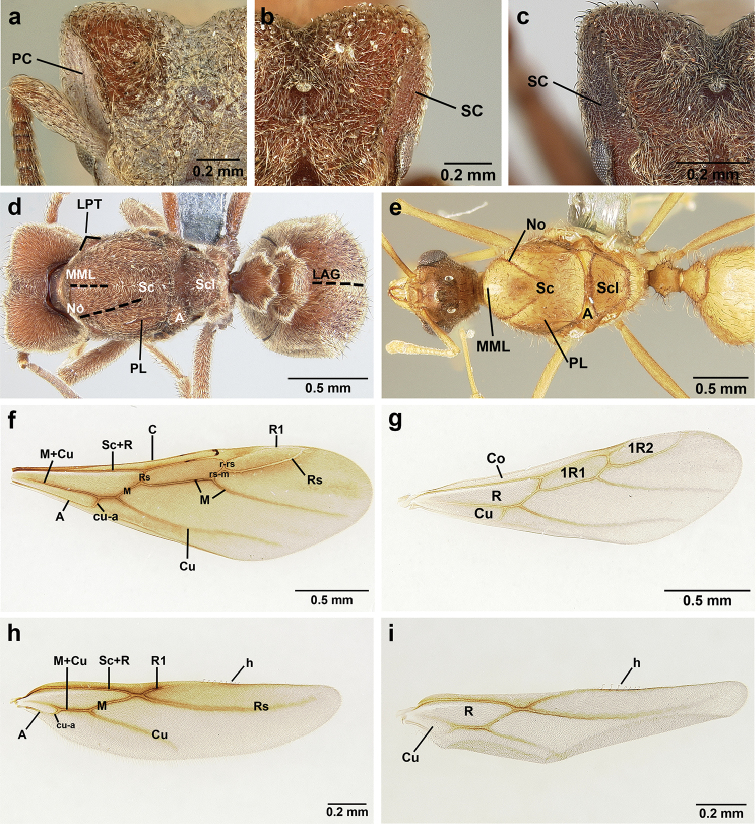
Queen and male morphology. **a** *S. maravalhas* queen head, full-face view, preocular carina **b** *S. parvulus* queen head, full-face view, supraocular carina; **c** *S. bondari* queen head, full-face view, supraocular carina **d** queen, dorsal view; **e** male, dorsal view **f** queen forewing, veins indicated **h** queen hindwing, veins indicated **g** male forewing, cells indicated; **i** male hindwing, cells indicated. **PC** Preocular carina **SC** supraocular carinae **MML** median mesoscutal line **No** notaulus **LPT** lateral pronotal tubercle **PL** parapsidal line **A** axilla **Sc** scutum **Scl** scutellum **LAG** longitudinal anteromedian groove **h** hamuli; wing veins (**f, h**): **C** costa; **Sc+R** subcosta+radius **M** media **Cu** cubitus **M+Cu** media-cubitus **A** anal vein **cu-a** cubitus-anal **Rs** radial sector **R1** radius; wing cells (**g, i**): **Co** costal; **R** radial **Cu** cubital **1R1** first radial 1 **1R2** first radial 2.

**Head.** In full-face view cordate, cephalic emargination distinct. Vertexal tumulus more pronounced than in worker, bearing glossy, light yellow to dark grey ocellus, integument surrounding ocellus sometimes darker than elsewhere. Ocelli half embedded in integument and covered with hair so in full-face view usually only median anterior ocellus visible. Preocular carina in some species fading posterior to eye, as in worker. In one species, preocular carina extending beyond eye, becoming thinner posterad and almost meeting frontal carina to form complete scrobe (Figure 8a). In third state preocular carina fades posterior to eye but one to several short, weak, isolated supraocular carinae are visible, not reaching posterior cephalic corner (Figure 8b–c). Eye larger and more convex than in worker, without white layer (except partially in eye of *S. saussurei* queen). Frontal lobe as in worker or more robust.

**Mesosoma** (Figure 8d–e). Lateral pronotal tubercles conical, short and blunt, best seen in dorsal view. Anapleural suture (=median episternal groove) wide, shallow, curved in lateral view, anepisternum inflated. Scutum in dorsal view with notauli reduced, not converging medially, forming faint, shallow impression, sometimes entirely absent. Median mesoscutal line absent or with only anterior portion visible, sometimes forming weak costa, posteriorly with shallow longitudinal impressions at each side. Parapsidal lines thin, slightly curved. Axillae in dorsal view laterally rounded, narrowing medially, entirely separated from one another by shallow groove, groove sometimes transversely costate. Scutellum slightly convex in lateral view, in dorsal view narrowing posteriorly, posterior margin with wide, shallow, median V-shaped notch, notch sometimes continuing into median impression that divides scutellum in two lateral parts. Propodeum in dorsal view with two low, posteriorly diverging carinae, often reduced to laterally flattened, obtuse denticles.

**Wings** (Figure 8f, h). Terminology follows Goulet and Huber (1993) and Yoshimura and Fisher (2012). Light to dark brown, opaque, covered with minute pilosity, veins brown. Forewing (length: 4.85–8.03 mm) with following veins: costa (C), Sc+R, media (M), cubitus (Cu), anal vein (A), radius (R1), radial sector (Rs), M+Cu, r-rs, and cu-a. Radial sector reaching costal margin, cubitus and media weaker towards wing margin, anal vein not extending distad after cu-a, cubitus in *S. mayri* sometimes with 1–2 short spur veins distally. Five closed cells: costal (C), radial (R), cubital (Cu), first radial 1 (1R1), and first radial 2 (2R1). Pterostigma weakly developed.

Hindwing (Length: 6–5.28 mm) with reduced venation: Sc+R, radial sector (Rs), M+Cu, cubitus (Cu), media (M), anal vein (A), and cu-a. Two enclosed cells: radial and cubital, cubital much smaller. Radial sector vein and media not reaching wing margin, cubitus in *S. mayri* sometimes with 1–3 short spur veins distally. Anterior margin of hindwing with 7–9 hamuli, varying within species.

**Metasoma.** Petiole compact, without subpetiolar process, petiolar peduncle short. Petiole with two dorsal denticles, often acute, more prominent than in worker, and with two smaller lateral denticles, best seen in frontodorsal view. Postpetiole broader than long and broader than petiole in dorsal view, with two dorsal and two lateral short longitudinal carinae, sometimes reduced to low denticles, best seen in frontodorsal view. Size and sharpness of petiolar denticles usually correlated with body size. First gastral tergite (A4) larger and longer than first sternite, laterally impressed on both sides, with pair of strongly developed lateral longitudinal carinae, dorsally with shallow longitudinal anteromedian groove (Figure 8d), anteromedian dorsal carinae on each side of groove moderately developed to absent.

### *Sericomyrmex* male

(Genus-level description of *Sericomyrmex* male based on *S. amabilis*, *S. lutzi*, *S. mayri*, *S. opacus*, and *S. saussurei*. Males of *S. bondari*, *S. maravalhas*, *S. parvulus*, *S. radioheadi*, *S. saramama*, and *S. scrobifer* unknown.)

**General appearance.** Body color pale yellow-brown to dark brown, head darker, antennae and legs lighter than rest. Body covered with fine pubescence and thicker hairs, sparser than in worker and queen. Area around ocellus and petiolar and postpetiolar dorsum with more hair; mesosoma and gaster, dorsally and laterally, with less hair. Integument relatively dull, with reticulate sculpture, but shinier than very opaque integument of worker and queen. SEM images indicate males lack waxy, crystal-shaped cuticular layer, present in workers and queens (Figure 6f).

**Head.** In full-face view obovate to subquadrate, as long as wide to longer than wide (CI=100–133), posterior cephalic margin straight, without emargination, lateral margin straight or slightly convex. Vertex with three large, white to grey ocellus, mounted on sides of small tumuli. Clypeus in full-face view broadly convex. Clypeal apron with shallow median notch, median clypeal seta arising from its middle. Mandible triangular, with lateral margin straight, except curving at apex, dorsal surface finely reticulate near the basal angle and with thin, light yellow, sparse hairs, directed apically. Masticatory margin with 5–7 teeth, usually 4–5 teeth in distal two-thirds, gap between single basal tooth and rest (Figure 18i); sometimes this gap absent and teeth distributed more or less evenly (Figure 48b). Palp formula: 4, 2. Preocular carina varying in length, directed posterad, curved medially before fading. Eye convex, large, protruding laterally, 20–28 ommatidia across largest diameter. Frontal lobe small and narrow, not completely concealing condylar bulb, without clearly defined medial, lateral, and posterior margin. Frontal carina absent. Antennal scape long, straight, extending well beyond posterior cephalic corner (SI=74–95). Antenna 12-segmented, without distinct club, pedicel thicker and longer than funicular segments two or three.

**Mesosoma** (Figure 8e). Anteroventral pronotal corner obtuse, never denticulate. Lateral pronotal tubercles absent. Mesopleuron with shallow anapleural suture. Katepisternum inflated and rounded ventrally, best seen in lateral view. Scutum in dorsal view with notauli distinct, almost converging posteriorly. Median mesoscutal line faint, best visible in antero-dorsal view, fading posteriorly. Parapsidal lines thin, slightly curved. Notauli, mesoscutal, and parapsidal lines sometimes lighter than surrounding integument and sometimes darker; this variation seen within males from same nest. Axillae rounded laterally, entirely separated from one another by shallow groove, groove sometimes transversely costate. Scutellum inflated, convex in lateral view, in dorsal view narrowing posteriorly, posterior margin straight or with shallow medial notch. Propodeum dorsally completely smooth or with two low, short propodeal carinae. Propodeal spiracle directed posterad, mounted on small tubercle, best seen in dorsal view, propodeal spiracular carina absent.

**Wings** (Figure 8g, i). Venation and cells same as in queen, wings smaller and lighter in color. Forewing length: 3.48–6.25 mm, hindwing length: 2.20–4.29 mm. Anterior margin of hindwing with 7–10 hamuli.

**Metasoma.** Petiole with short peduncle, without subpetiolar process, longer than postpetiole in lateral view, with pair of small denticles laterally and sometimes also dorsally. Postpetiole in dorsal view broader than long, broader than petiole, laterally with pair of very reduced denticles, dorsally smooth. Gaster in dorsal view elliptical, without any carinae. First gastral tergite and sternite equal in length and size, gastral tergites and sternites 2–5 (i.e. A5 to A8) visible in dorsal view and with rows of long, decumbent to suberect hairs on posterior borders, hairs on dorsal side slightly longer than ventral hairs.

**Figure 9.**
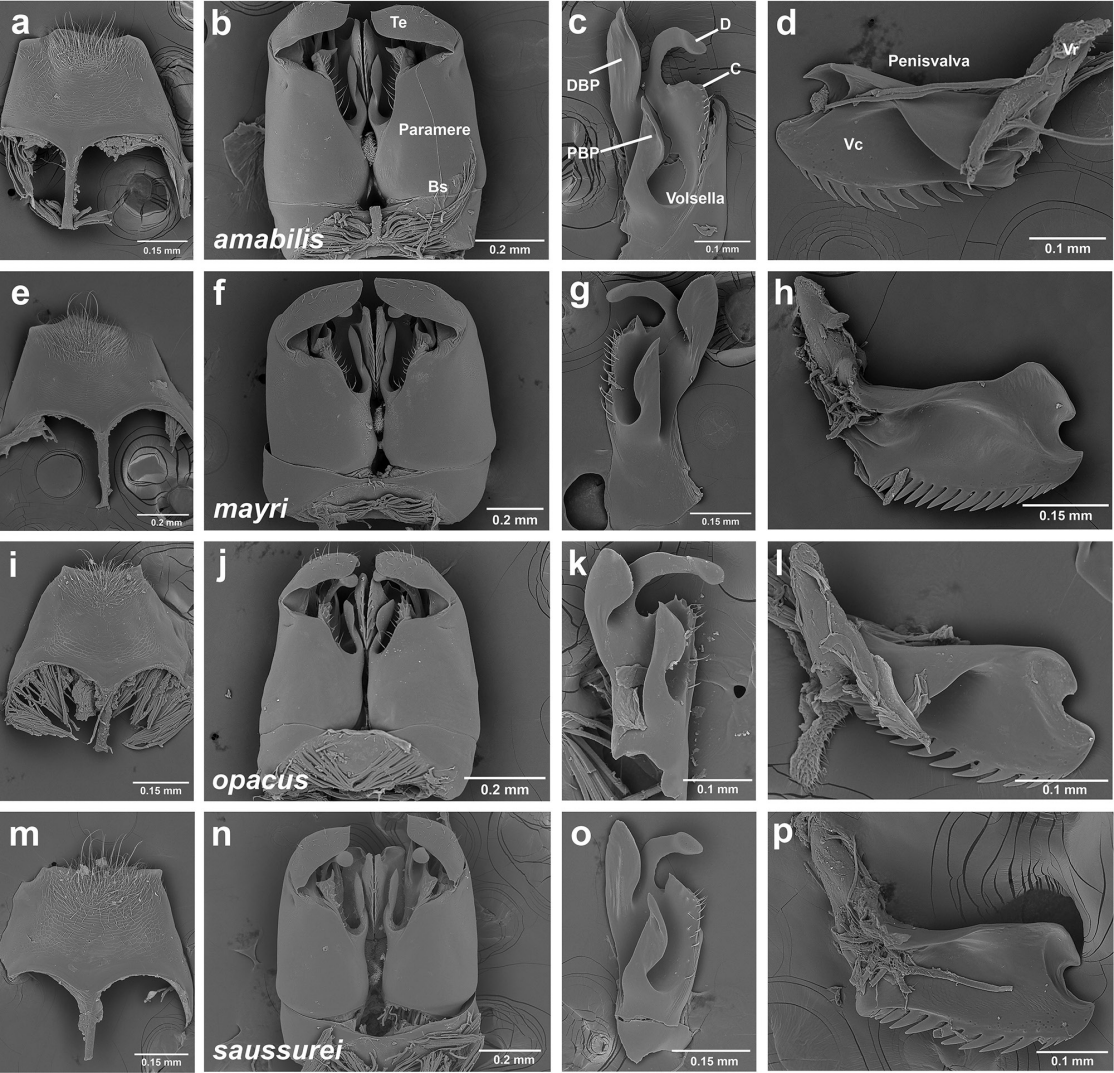
*Sericomyrmex* male genitalia. *S. amabilis* (**a**–**d**) *S. mayri* (**e**–**h**) *S. opacus* (**i**–**l**) *S. saussurei* (**m–p**). Abdominal sternum IX, ectal view (**a, e, i, m**) genital capsule, ventral view (**b, f, j, n**) volsella, mesal view (**c, g, k ,o**) and penisvalve, mesal view (**d, h, l, p**). **Bs** Basimere **Te** telomere **D** digitus **C** cuspis **DBP** distal basivolsellar process **PBP** proximal basivolsellar process **Vr** valvura **Vc** valviceps.

**Genitalia** (Figure 9). Abdominal sternum IX with long and thin spiculum, lateral margins extending anteriorly about length of spiculum, tapering posteriorly, posterior margin straight, without apical triangle (Figure 9i, m) or with apical triangle low (Figure 9a, e), surface weakly reticulate and with simple hairs posteriorly. Basimere smooth, longer than broad, telomeres short, medially curved and bluntly rounded apically, with sparse simple hairs. Volsella with strongly medially curved, clubbed digitus, cuspis ventrally produced into rounded lobe, sometimes with 1–2 teeth, with thin, sparse, medially pointed hairs. Volsella basally with two additional processes, best seen in ventral view, here named proximal basivolsellar and distal basivolsellar process, previously undescribed structures of male ant genitalia. Valviceps of penisvalve with 10–12 long, pointed denticles along ventral edge, distally broadly notched.

### *Sericomyrmex* larva

Description based on SEM study of 23 prepupal larvae from nine different nests of *Sericomyrmex amabilis*, *S. bondari*, *S. mayri*, *S. opacus*, *S. parvulus*, *S. saramama*, and *S. saussurei*. Larvae for *S. lutzi*, *S. radioheadi*, *S. scrobifer*, and *S. maravalhas* unknown. Terminology follows Schultz and Meier (1995), including “seta” (with visible basal setal socket) and “hair” (without visible socket). We only use the term seta, since the socket is always visible. When setal base is visible but seta is either absent or very short, we use sensilliform or papilliform, respectively.

**Figure 10.**
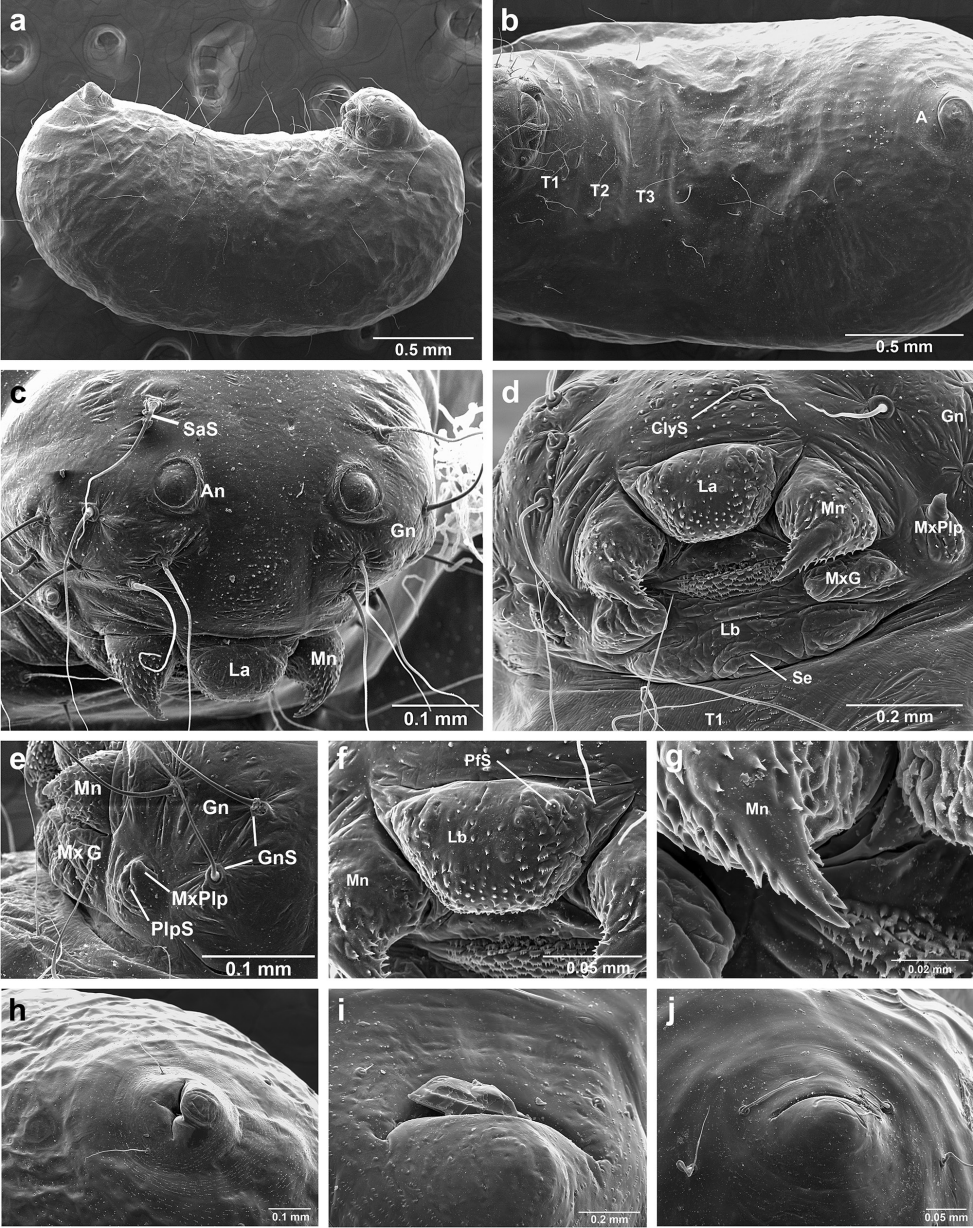
*Sericomyrmex* larval morphology, SEM images. **a** Lateral view **b** ventral view **c** head, frontodorsal view **d** mouthparts, ventral view **e** head, lateral view **f** labrum detail **g** mandible detail **h, i, j** anal setae. **T1**, **T2**, **T3** Thoracic segments 1, 2, and 3 **A** anus **SaS** supra-antennal seta **An** antenna **Gn** gena **La** labrum **Mn** mandible **MxG** maxillary galea **Lb** labium **Se** sericteries **MxPlp** maxillary palp **ClyS** clypeal seta **GnS** genal setae **PlpS** maxillary palp sensillum **PfS** papilliform seta.

Body profile “attoid” *sensu* Wheeler and Wheeler (1948), i.e., longitudinally curved, bean-shaped, and with ventral profile shorter than dorsal (Figure 10a). Thoracic-abdominal articulation absent, thoracic intersegmental constrictions superficial, deep lateral depressions associated with abdominal spiracles absent, and leg vestiges visible as open slits ventrally on thorax. Dorsal and lateral body surfaces from bare (*S. parvulus*) to with more than 15 setae on each side (*S. amabilis*). Setae simple, some long and flexuous, some (e.g., on clypeus and labrum) reduced, papilliform, with only setal socket and very short bristle (Figure 10f: PfS).

**Head.** Genal lobe present (Figure 10c–e: Gn). One long supra-antennal seta (Figure 10c: SaS) posterior to each antenna (“*Sericomyrmex* condition” *sensu* Schultz and Meier 1995) in some species (*S. amabilis*, *S. bondari*, *S. mayri*), absent in others (*S. opacus*, *S. parvulus*, *S. saramama*, *S. saussurei*). No setae between antennae, four setae on each gena, except in *S. mayri* with six. Two supraclypeal setae in all species, reduced to papillae, except *S. bondari* with two long supraclypeal setae (Figure 10d: ClyS). Spinules on head restricted to clypeus, genae, and vertex; in *S. opacus* almost completely absent, restricted to few on clypeus.

**Mouthparts.** Labrum monolobate, narrow, inflated, with anterior setae absent or reduced to sensilla or papillae. Mandible fleshy and subconical, covered with spinules. Subapical mandibular tooth absent, mandibular apical tooth distinct, divided in some species (Figure 10g), undivided in others. Mandibular gnathobases absent. Basal portion of maxilla fused with head capsule, maxillary palp digitiform, widely removed laterad from galea. Maxillary accessory palpal sensillum present (Figure 10e: PlpS). Area between maxillary galea and palp with two reduced setae. Labium short, only feebly protruding, lateral sericteral protuberances absent, labial palps reduced to sensilla. Spinules on labium completely absent or sparse, present only anterior to sericteries. Ventral part of labium (posterior to sericteries) usually not visible. Hypopharyngeal spinules densely distributed and predominantly multidentate.

**Thorax and abdomen.** Thoracic segment one (T1) ventrally with transverse rows of sparsely distributed multidentate spinules (except *S. saussurei*). Thoracic segments two and three (T2 and T3) without multidentate spinules, except in *S. bondari*, which has sparse spinules on T2. Number of setae on ventral thoracic segments varies from 0–5 on each side of each segment, in general T1 with more setae than T2 or T3. Less than ten setae on T1 in most species, except *S. mayri* with 10–14 setae on T1, the number of setae considered sufficient to form, in combination with genal setae (six in *S. mayri*), “feeding basket” of attine ant larvae (Schultz and Meier 1995). Abdominal segments lacking any ventromedian protuberances, with variable numbers of long and simple setae, except in *S. parvulus*, which has no ventral abdominal setae. Anal setal pattern: single pair of papilliform to long setae anterior to anal opening (Figure 10h–i). Additional pair of setae sometimes ventrolaterally on segment nine (e.g., in *S. bondari*), but widely removed (Figure 10j). Ventral anal lip absent.

### *Sericomyrmex* genus-level differential diagnosis

Ants of the genus *Sericomyrmex* are small- to medium-sized, larger than ants of most of the lower-attine ant genera (e.g., *Cyphomyrmex*, most *Apterostigma*) and similar in size to most *Trachymyrmex* species and to the media workers of leaf-cutter ants (*Atta* and *Acromyrmex*). They are monomorphic to slightly polymorphic and can easily be separated from other attine ant genera by the following combination of characters: a silky, woolly appearance due to dense pilosity and hair; cordate head shape; well-developed frontal lobes; robust and short, moderately tuberculate mesosoma; and a smooth gaster, lacking tubercles. Members of the *Trachymyrmex iheringi* clade are similar to *Sericomyrmex* in size, general appearance, and the presence of long, flexuous hairs in some species (e.g., *T. opulentus* Mann and *T. dichrous* Kempf), but *Sericomyrmex* can be distinguished from them by the following: posterior cephalic corners in full-face view smooth (most *Trachymyrmex* with multiple small to large denticles, tubercles, or spines, with exception of *T. dichrous*); antennal scape short, in full-face view almost reaching cephalic margin but never surpassing it (scape longer in *Trachymyrmex*, always surpassing cephalic margin); frontal lobes wide, concealing the base of the scape, with three distinct margins (in *Trachymyrmex* often rounded or with just two margins, smaller and narrower, often not concealing the base of the scape); tubercles on mesosoma smooth, simple, not bearing minute tubercles (tubercles on tubercles are very common in *Trachymyrmex*, giving the mesosoma a spiny appearance); gaster smooth, unsculptured, if mildly tuberculate this is visible only under SEM (in *Trachymyrmex* the gaster often visibly tuberculate). Because of the absence of denticles and spines on the posterior cephalic corners, dense hair, and tubercles on the mesosoma without additional, minute tubercles, *T. dichrous* can be mistaken for a *Sericomyrmex* species. It is easily identified and separated from *Sericomyrmex* by its bicolored integument (head dark brown, rest of body light ferrugineous brown), narrow and rounded frontal lobes, and strongly convex eyes protruding from the sides of the head.

Ants of the genus *Apterostigma* Mayr can be separated from *Sericomyrmex* as follows: head long, narrowing posterad of the eyes (except for *A. megacephala* Lattke); posterior cephalic border evenly rounded, never medially emarginate (median emargination always present in *Sericomyrmex*); frontal lobes frequently rounded (rectangular or triangular with three distinct margins in *Sericomyrmex*); mesosoma and general appearance slender, similar to some non-attine ant Myrmicinae genera (e.g., *Aphaenogaster* Mayr) (*Sericomyrmex* habitus more robust).

Males of *Sericomyrmex* can be separated from most other attine males by the presence of 12 antennal segments, deviating from the usual 13 segments in the males of most attine species, and from the 11 segments found in males of some social parasites (Gallardo 1916, Schultz et al. 1998, Rabeling and Bacci 2010). However, 12 antennal segments occur in parallel in *Cyphomyrmex* (e.g., *C. faunulus* Wheeler and *C. auritus* Mayr), *Mycetophylax conformis* Mayr, *Mycetagriocus inflatus* Brandão & Mayhé-Nunes, and *Trachymyrmex opulentus* (Mayr 1887, Wheeler 1925a, Ješovnik et al. 2013, Klingenberg and Brandão 2009). Males of *Sericomyrmex* can be separated from the other attine ant males with 12-segmented antennae by the following: presence of long, flexuous hairs on most of the body (no hair or very short and sparse hair in *M. inflatus*, *Cyphomyrmex*, *Mycetophylax*, and *Trachymyrmex*); dull, light brown integument, faintly reticulate and not strongly sclerotized (strongly sclerotized and dark brown to black in *C. faunulus* and *M. conformis*, light brown but costate on head and mesosoma in *Trachymyrmex*), scutellum with flat to slightly notched posterior margin, but never with sharp and long posterior processes (as in *Trachymyrmex*, *C. faunulus*), smooth posterior cephalic corners (with a small sharp denticle in *C. faunulus* and *C. auritus*).

The larvae of *Sericomyrmex* can easily be recognized as those of attine ants because of the following synapomorphies: short, narrow, monolobate labrum; fleshy subconical mandibles; leg vestiges present as open slits in the integument; and a reduced number of supra-antennal setae (one to no setae in *Sericomyrmex*). The larvae of *Sericomyrmex* can be separated from larvae of closely related *Trachymyrmex* species by anal hairs in a typical “*Sericomyrmex* pattern” (sensu Schultz and Meier 1995): a single pair of hairs anterior to the anus (5–8 extremely short to sensilliform setae in the *Trachymyrmex iheringi* group) and the presence of setae on the lateral and dorsal surfaces of the body in most species (setae absent in *Trachymyrmex*).

There are some characters that *Sericomyrmex* larvae share with the closely related *M. explicatus*, in particular labial spinules that are present only on the anterior surface dorsal to sericteries (ventral side of labium hidden) and the absence of supra-antennal setae (in some species of *Sericomyrmex*). The larva of *M. explicatus* differs from *Sericomyrmex* larvae by the anal setae pattern: *M. explicatus* with ~eight extremely short setae anterad and 0–2 setae dorsad of the anus, whereas *Sericomyrmex* species we examined have two setae anterad, 0–2 setae dorsad; and by the number of genal setae: three in *M. explicatus*, 4–6 in *Sericomyrmex*.

### Character discussion and terminology definitions

**Worker**

**General appearance.** Worker color varies within species and even within single colonies and can be altered by collecting and preservation methods as well as by age, especially in dried, pinned specimens. The body color of an individual *Sericomyrmex* worker is homogeneous except in some *S. bondari* individuals from Carajás, Brazil (Figure 23a–c), and in a few workers of *S. saussurei* in which some, but not all, nestmate workers have lighter-colored areas on their heads. We refer to thicker, non-pubescent hairs simply as “hairs,” whereas, when referring to pubescence, we use the terms “pubescence” or “pubescent hairs.” The density of pubescence may vary both between and within species. Hair length, color, and degree of curvature (appressed to nearly erect) vary within species. Curvature in particular can be altered by collecting and preservation methods; for example, the hair of specimens collected by pitfall trapping may differ from that of workers from same nest collected directly into ethanol and subsequently point-mounted. Hair is, however, a useful character for recognizing the species *S. bondari*, which possesses uniquely thick, dark hairs (Figure 22).

**Body size**. Body size is a useful character for separating some species. Because it varies to some extent within species and within colonies, it should be used with caution and in combination with other characters. We use the combination of head width including the eyes (HWe), Weber’s length (WL), and hind femur length (HFL) as an approximation for body size to group *Sericomyrmex* species into three size categories. The mean values for the size categories are as follows: small (HW<0.92, WL<1.18, HFL<0.99); medium (HW=1.0–1.06, WL=1.28–1.39, HFL=1.1–1.24); and large (HW>1.15, WL>1.44, HFL>1.27).

**Head.** The head in *Sericomyrmex* is characteristically cordate, but the shape of the posterior corners and the size and shape of the posterior cephalic emargination vary between species. Here we distinguish between three main categories of posterior corner shape: 1) *acute*, in which (in dorsal, i.e., full-face view) the lateral and posterior cephalic margins meet at an angle of less than 90 degrees (Figure 7d); 2) *angular*, in which these margins meet at an angle approaching 90 degrees and in which the lateral and posterior cephalic margins are more or less straight (Figure 7e); and 3) *rounded*, in which the transition between the lateral and posterior margins is smoothly rounded and in which the lateral and posterior borders are usually convex (Figure 7f). Even though intermediate states exist, these three character states are very useful in describing the variation across *Sericomyrmex* species and, in combination with other characters, are useful in distinguishing species.

The shape of the median cephalic emargination varies in a manner that can be broadly captured by two states: it can be gradually impressed (Figure 7j) or it can be abruptly impressed (Figure 7k). Intermediate states also exist and in some species, particularly *S. amabilis* and *S. saussurei*, both states are often found within the same colony. However, the second (abruptly impressed) state predominates in the species *S. mayri*, whereas the gradual state almost always occurs in the species *S. bondari*, *S. lutzi*, *S. maravalhas*, *S. opacus*, *S. parvulus*, *S. radioheadi*, *S. saramama*, and *S. scrobifer*.

**Mandibles.** In most species (*S. bondari*, *S. lutzi*, *S. maravalhas*, *S. opacus*, *S. parvulus*, *S. radioheadi*, *S. saramama*, *S. scrobifer*) the dorsal surfaces of the mandibles are glossy and smooth, lacking any striae except for very short and faint ones restricted to the masticatory margin. In *S. amabilis*, *S. saussurei*, and *S. mayri*, however, the mandibles are dorsally striate (Figure 6g–h). Exceptions occur in each species: smooth mandibles occur in some populations of *S. amabilis* in Costa Rica, Ecuador, and Colombia, in populations of *S. saussurei* mostly in southeast Brazil, and in populations of *S. mayri* in Trinidad and Tobago and in Suriname. In callow workers of all species, dorsal striation is not very distinct (Figure 6i). An intermediate stage exists in all three species, with mandibles just partly or faintly striate. Because the presence of smooth mandibles may indicate either that a specimen belongs to one of the smooth-mandibled species or that it belongs to a rare population of an otherwise striate-mandibled species, additional characters obviously need to be examined for species diagnoses. The presence of striate mandibles, in contrast, always indicates that a specimen belongs to one of the three species with striate mandibles. There are no consistent differences between species in the number of teeth; in fact, the opposite mandible of a single specimen may have different numbers of teeth.

**Eyes.** Relative eye size, reflected in the ocular index (OI), varies moderately within most species (OI 14–16), but it is noticeably larger in two species, *S. scrobifer* and *S. maravalhas* (OI 18–19). For describing eye size, we use three categories based on OI value: “small” eyes for species with OI=14, “medium-sized” eyes for species with OI between 15–16 (the majority of species), and “large” eyes for species with OI 18–20. Eyes can be flat to strongly convex, the latter protruding from the lateral borders of the head. Most species have flat to mildly convex eyes, but the eyes of *S. scrobifer* and *S. saussurei* are consistently moderately (*S. saussurei*) or strongly (*S. scrobifer*) convex, so eye shape is useful for recognizing those species.

Another important character of the eyes is the presence or absence of a white, waxy layer. This layer may be absent (Figure 6j–k); it may only partially cover the eyes (in *S. parvulus*, Figure 6l), or it may completely cover them. When completely covering the eye it can be relatively thin, so that the individual ommatidia are still discernible (as in *S. opacus*, Figure 6m), or it can be so thick that individual ommatidia are hard to distinguish (as in *S. saussurei*, Figure 6o), even though in some individuals the centers of individual ommatidia can be seen showing through the white layer in rare adult individuals (as in some *S. saussurei*, Figure 6n). Below we refer to this layer simply as a “white layer”; its origin, whether as a cuticular secretion or due to some other cause, remains the subject of future investigation.

**Frontal lobes.** Frontal lobe size and shape are useful characters for *Sericomyrmex*, and here we distinguish three main states: 1) *trapeziform* (Figure 7g), in which the posterior margin of the frontal lobe is subequal in length to the medial margin and in which the angles between the lateral and the other two margins are subequal and often acute; 2) *rectangular* (Figure 7h), similar to trapeziform, but in which the medial and posterior margins are parallel and in which the angles between the lateral and the other two margins approximate 90 degrees; and 3) *triangular* (Figure 7i), in which the posterior margin is at least half the length of the medial margin, sometimes much less than half; in which the angle between the lateral and posterior margins is obtuse; and in which the angle between the lateral and medial margins is acute, and the lobe is directed anterad. Although variants and intermediate states occur, and some degree of variation frequently occurs between individuals in the same nest, frontal lobe shape remains a very informative character. The frontal lobes of *S. scrobifer*, for example, can vary from trapeziform to rectangular, but are never triangular; and the frontal lobes of *S. bondari* are always triangular and narrow, with long lateral margins. Among these three states, triangular is the one with the most variation.

**Frontal carinae.** The length, shape, and robustness of the frontal carinae vary between species, but also to some extent within species. Frontal carinae can be complete and robust or, at the opposite extreme, weak and incomplete, fading before reaching the posterior corners. Complete and robust frontal carinae (Figure 7g: CFC) are a good diagnostic criterion for some species (*S. scrobifer*, *S. maravalhas*), but the alternate state of reduced frontal carinae can be fairly variable within species, e.g., *S. parvulus* and *S. mayri* mostly have reduced frontal carinae (Figure 7f: IFC), but in some individuals or populations this is not the case, so this character must be examined across nest series and employed in conjunction with other characters.

**Scape length.** Scape index is a useful character only in combination with other characters and is diagnostic only for *S. radioheadi*, which has an unusually long scape (Figure 3: SI).

**Mesosoma and metasoma.** The mesosomal tubercles and propodeal carinae and denticles are in general sharper and longer in some species and blunter and shorter in others. However, because within-species and even within-colony variation are common, this character must be used with caution. The same holds for the petiolar and postpetiolar denticles and carinae, which, again, can be more or less pronounced within workers of the same colony. The presence or absence of a pair of anteromedian dorsal carinae on the first gastral tergite (Figure 7c) is a more consistent character that in general holds well for *S. scrobifer* and *S. maravalhas*, in which it is strongly developed, and for *S. parvulus* and *S. opacus*, in which it is faint or completely absent. In *S. amabilis* and *S. mayri* it is variable (usually well developed but faint in some specimens).

**Queen**

Characters that are useful for separating workers are also useful for separating queens, especially size, mandibular striation, and frontal lobe shape. A queen-specific character that is useful for separating queens of *S. maravalhas* from queens of similarly sized species is the presence of a full preocular carina (Figure 8a). Queens of other species either have the preocular carinae ending posterior to the eye or have a few isolated, short, faint supraocular carinae that do not reach the posterior cephalic corners. The notauli, median mesoscutal line, and parapsidal lines, as well as the depth of the scutellar notch, can vary from prominent to very faint, but these variations seem to be as common within species as between species and are thus not useful for separating species.

**Male**

Male genitalia show differences in the following characters: the apical triangular lobe on abdominal sternum IX (present/absent) and the volsellar cuspis either with a simple lobe (*S. amabilis*, Figure 9c), with a single tooth (*S. mayri*, *S. saussurei*, Figure 9g, o), or with several minute denticles (*S. opacus*, Figure 9k). Further research is needed to determine character-state variability and distributions in *Sericomyrmex* male genitalia. Apart from genitalia and body size, we found no morphological characters of males useful for separating species, but this might be due to the small sample sizes and to the fact that we examined males for only five out of 11 species.

**Larva**

Based on examination of the larvae of seven species, larval characters useful for separating *Sericomyrmex* species are the number of dorsal and lateral setae (ranging from absent in *S. saramama* and *S. parvulus* to ~30 in *S. amabilis*), supra-antennal setae (presence/absence), mandibular teeth (divided/undivided), number of genal setae (six in *S. mayri*, four in all others), the presence of an additional pair of anal setae (*S. bondari* only), and the number of setae on T1.

### Taxonomic synopsis

(w–worker, m–male, q–queen, l–larva)

***Sericomyrmex amabilis*** Wheeler 1925b, Mexico to northwestern South America (w, q, m, l).

=*Sericomyrmex bierigi* Santschi, 1931, synonymy by Weber (1958).

***Sericomyrmex bondari*** Borgmeier, 1937, Colombia to Bolivia and Brazil (w, q, l).

=*Sericomyrmex beniensis* Weber, 1938, **syn. n.**

***Sericomyrmex lutzi*** Wheeler, 1916, Guyana (w, q, m).

***Sericomyrmex maravalhas*** Ješovnik & Schultz, Brazil (w, q), **sp. n.**

***Sericomyrmex mayri*** Forel, 1912, Colombia to Bolivia and Brazil (w, q, m, l).

=*Sericomyrmex urichi* Forel, 1912, **syn. n.**

=*Sericomyrmex luederwaldti* Santschi, 1925, **syn. n.**

=*Sericomyrmex moreirai* Santschi, 1925, **syn. n.**

=*Sericomyrmex harekulli* Weber, 1937, **syn. n.**

=*Sericomyrmex harekulli arawakensis* Weber, 1937, **syn. n.**

***Sericomyrmex opacus*** Mayr, 1865, Mexico to northwestern Brazil (w, q, m, l).

=*Sericomyrmex aztecus* Forel, 1885, **syn. n.**

=*Sericomyrmex diego* Forel, 1912, **syn. n.**

=*Sericomyrmex zacapanus* Wheeler, 1925a, **syn. n.**

***Sericomyrmex parvulus*** Forel, 1912, Colombia to Bolivia and Brazil (w, q, m, l).

=*Sericomyrmex myersi* Weber, 1937, **syn. n.**

***Sericomyrmex radioheadi*** Ješovnik & Schultz, Venezuela (w), **sp. n.**

***Sericomyrmex saramama*** Ješovnik & Schultz, Colombia, Ecuador, Peru (w, q, l), **sp. n.**

***Sericomyrmex saussurei*** Emery, 1894, Colombia to Bolivia and Brazil (w, q, m, l).

=*Sericomyrmex burchelli* Forel, 1905, **syn. n.**

=*Sericomyrmex impexus* Wheeler, 1925a, **syn. n.**

= *Sericomyrmex urichi maracas* Weber, 1937, **syn. n.**

***Sericomyrmex scrobifer*** Forel, 1911, Brazil, Paraguay (w).

### Key to the worker caste of *Sericomyrmex*

When we refer to sizes or shapes we provide mean values, indicated with bold font, followed by ranges. Because of frequent overlap of minimum values of one species with maximum values of another, we advise users of this key to examine several individuals from the same nest/collection event whenever possible and to consult images and distribution maps when indicated. The mean values for the size categories are as follows: small (HW<0.92, Wl<1.18, HFL<0.99), medium (HW=1.0–1.06, WL=1.28–1.39, HFL=1.1–1.24), and large (HW>1.15, WL>1.44, HFL>1.27). All measurements are in millimeters.

**Table:** 

1	Mandible dorsally smooth except for fine transverse striae along the masticatory margin (Figure 6g, Figure 11a)	**2**
–	Mandible striate across the most or all of the dorsal surface (Figure 6h, 11b)	**12**
2 (1)	Entire body covered with thick, dark hair (Figure 11c). Large species (HWe=**1.15**, 0.96–1.4; WL=**1.44**, 1.25–1.76; HFL=**1.27**, 1.12–1.52), posterior cephalic emargination deep, frontal lobe narrow, triangular, directed anterad (Figure 22a)	***bondari***
–	Body not covered with thick, dark hair (Figure 11d); small to large species; posterior cephalic margin from shallow to deep; frontal lobe variable	**3**
3 (2)	Mesosoma with very long and sharp lateral mesonotal tubercles (Figure 12c); scape long (SI=**77**, 75–82), reaching the posterior cephalic corner in full-face view (Figure 12a); posterior cephalic emargination deep (CEI=**14**, 12–17); frontal lobe narrow, triangular; medium-sized species	***radioheadi***
–	Lateral mesonotal tubercles from low and obtuse to more developed, but never as long or as sharp as in *radioheadi* (Figure 12d); scape relatively short (Figure 12b), cephalic emargination shallow to deep, size small to large, frontal lobe variable	**4**
4 (3)	Cephalic emargination deep (CEI=**15**, 16–17) (Figure 13a); mesosomal tubercles low and obtuse; body size large (HWe=**1.22**, 1.12–1.35; WL=**1.52**, 1.38–1.68; HFL=**1.29**, 1.2–1.4); probably endemic to Mt. Roraima and surrounding tepuis in Guyana, Brazil, and Venezuela (Figure 30)	***lutzi***
–	Either body size small to medium (HWe<1.06, WL<1.39, HFL<1.24) and cephalic emargination shallow to moderately deep (CEI=9–12), or body size large (HWe=**1.35**, 1.05–1.6; WL=**1.71**, 1.27–2.20; HFL=**1.48**, 1.15–1.70) and cephalic emargination shallow (Figure 13b)	**5**
5 (4)	Eye large (OI>18), frontal carina complete, usually robust (Figure 14a); body size small or medium; gaster with two lateral and two dorsal carinae strongly developed (Figure 14c)	**6**
–	Eye medium-sized (OI 14–16), frontal carina complete to incomplete, but not robust (Figure 14b), gaster with lateral carinae weakly to strongly developed, dorsal carinae absent or faint (Figure 14d)	**7**
6 (5)	Frontal lobe wide, trapeziform (Figure 61a) (FLI=**76**, 72–83), eye large, strongly convex, and strongly protruding from the sides of the head, medium-sized species (HWe=**1.0**, 0.84–1.12; WL=**1.29**, 1.12–1.4; HFL=**1.11**, 0.93–1.26)	***scrobifer***
–	Frontal lobe triangular (Figure 32a), narrower than in *scrobifer* (FLI=**69**, 64–73), eye large, moderately protruding, small species (HWe=**0.92**, 0.8–0.98: WL=**1.18**, 1.03–1.28; HFL=**0.99**, 0.82–1.08)	***maravalhas***
7 (5)	Small to medium-sized species (WL=**1.04**–**1.28**, 0.74–1.4), head relatively small, usually as long as broad (HWe= **0.81**–**1.01**, 0.66–1.13; CI=**100**–**102**, 94–108), frontal lobe small and reduced, rectangular or triangular (Figure 15a–c)	**8**
–	Larger species (mean WL=**1.33**–**1.71**, 1.1–2.2) , head relatively large, usually broader than long (HWe=**1.02**–**1.34**, 0.88–1.6; CI=**104**–**107**, 94–115), frontal lobe either triangular and diverging, or triangular and very narrow, directed anterad (Figure 15d–f)	**10**
8 (7)	Small species (WL=**1.04**, 0.74–1.23; HWe=**0.82**, 0.66–0.90; HFL=**0.82**, 0.65–0.99); frontal carina incomplete to complete, usually faint; frontal lobe small and triangular; eye small and flat, often covered with thin white layer (Figure 14b, d)	***parvulus***
–	Small to medium-sized species (mean WL =1.15–1.28, mean HWe=0.9–1.01); frontal carina incomplete or complete; frontal lobe rectangular to triangular; eye larger and without white layer or smaller in size and with white layer (as in *parvulus*), but then frontal lobe rectangular	**9**
9 (8)	Small-sized species (WL=**1.16**, 0.99–1.30; HWe=**0.91**, 0.8–1.0; HFL=**0.92**, 0.8–1.02); frontal lobe rectangular (Figure 15b); head corners smoothly rounded; eye often with transparent white layer.	***opacus***
–	Medium-sized species (WL=**1.28**, 1.12–1.40; HWe=**1.01**, 0.90–1.13; HFL=**1.10**, 0.95–1.20); frontal lobe triangular (Figure 15c); eye without white layer	***saramama***
10 (7)	Eye distinctly convex, mildly protruding, covered with thick white layer that makes it difficult to discern individual ommatidia (Figure 15e, 16a); medium-sized species	smooth-mandibled variant of ***saussurei***
–	Eye flat to mildly convex, without white layer, ommatidia visible; medium-sized to large (WL=**1.37**–**1.71**; HWe=**1.06**–**1.35**)	**11**
11 (10)	Large species (WL=**1.71**, 1.27–2.20; HWe=**1.35**, 1.05–1.60; HFL=**1.48**, 1.15–1.70); head broad (CI=**108**, 101–115); posterior cephalic corner acute to rectangular; frontal lobe narrow (FLI=**63**, 59–68), directed anterad; frontal carina often incomplete (Figure 16c); distribution: Colombia to Bolivia and Brazil (Figure 41)	smooth-mandibled variant of ***mayri***
–	Medium-sized species (WL=**1.37**, 1.13–1.60; HWe=**1.06**, 0.88–1.21; HFL=**1.15**, 0.93–1.38); head narrower than *mayri* (CI=**104**, 97–111); posterior cephalic corner acute to rounded; frontal lobe relatively wide (FLI=**68**, 60–72), directed laterally; frontal carina complete (Figure 16b); distribution: Mexico to Venezuela and Ecuador (Figure 21)	smooth-mandibled-variant of ***amabilis***
12 (1)	Eye distinctly convex, mildly protruding, covered with thick white layer that makes it difficult to discern individual ommatidia (Figure 15e, 16a); medium-sized species	***saussurei***
–	Eye flat to mildly convex, without white layer, ommatidia visible; medium-sized to large species (WL=**1.37**–**1.71**; HWe=**1.06**–**1.35**)	**13**
13 (12)	Large species (WL=**1.71**, 1.27–2.20; HWe=**1.35**, 1.05–1.60; HFL=**1.48**, 1.15–1.70); head broad (CI=**108**, 101–115); posterior cephalic corner acute to rectangular; frontal lobe narrow (FLI=**63**, 59–68) , directed anterad; frontal carina often incomplete (Figure 16c); distribution: Colombia to Bolivia and Brazil (Figure 41)	***mayri***
–	Medium-sized species (WL=**1.37**, 1.13–1.60; HWe=**1.06**, 0.88–1.21; HFL=**1.15**, 0.93–1.38); head narrower than *mayri* (CI=**104**, 97–111); posterior cephalic corner acute to rounded; frontal lob relatively wide (FLI=**68**, 60–72), directed laterally; frontal carina complete (Figure 16b); distribution: Mexico to Venezuela and Ecuador (Figure 21)	***amabilis***

**Figure 11.**
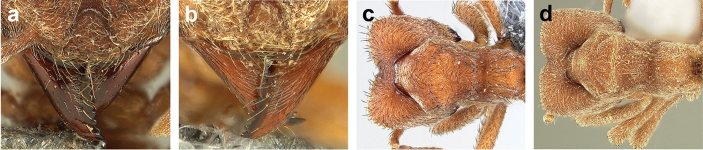
Mandibles and hairs. **a** Smooth mandibles (*S. scrobifer*) **b** striate mandibles (*S. saussurei*) **c** dorsal view of *S. bondari*: thick, dark hairs **d** dorsal view of *S. amabilis*: light yellow hairs.

**Figure 12.**
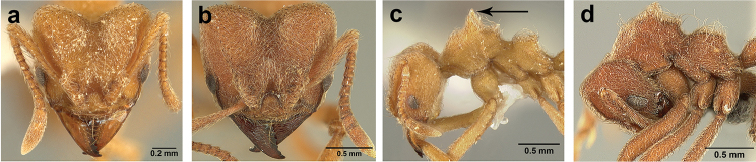
*S. radioheadi* and *S. mayri*. **a** *S. radioheadi* head, full-face view **b** *S. mayri* head, full-face view **c** *S. radioheadi* head and mesosoma, lateral view (arrow indicates lateral mesonotal tubercle) **d** *S. mayri* head and mesosoma, lateral view.

**Figure 13.**
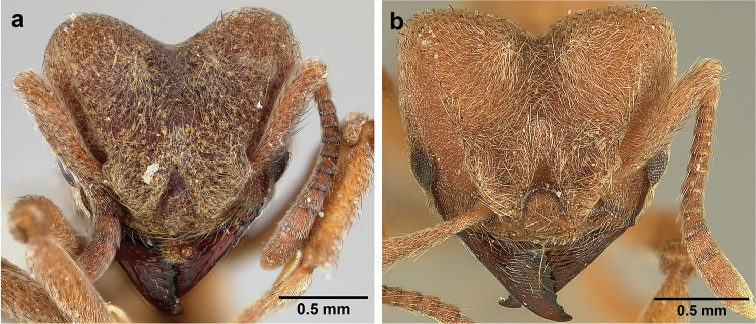
*S. lutzi* and *S. mayri*. **a** *S. lutzi* head, full-face view **b** *S. mayri* head, full-face view.

**Figure 14.**
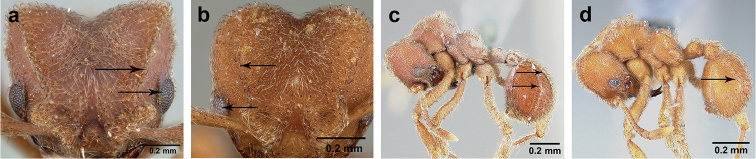
*S. scrobifer*, *S. parvulus*, and *S. maravalhas*. **a** *S. scrobifer* head, full-face view, arrows indicate complete frontal carina and large, convex eye **b** *S. parvulus* head, full-face view, arrows indicate incomplete frontal carina and small, almost flat eyes **c** *S. maravalhas*, lateral view, arrows indicate lateral and dorsal carinae on gaster **d** *S. parvulus*, lateral view, arrow indicates lateral carina on gaster, dorsal carina absent.

**Figure 15.**
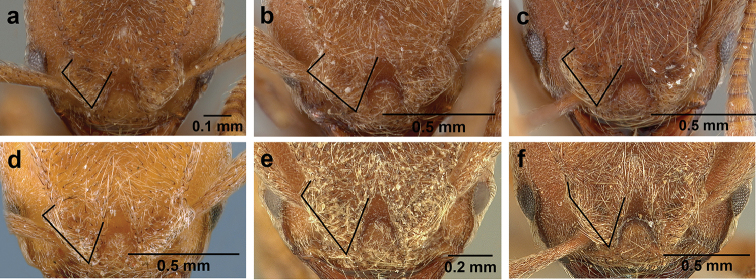
Frontal lobes. **a** *S. parvulus* **b** *S. opacus*; **c** *S. saramama* **d** *S. amabilis* **e** *S. saussurei* **f** *S. mayri*.

**Figure 16.**
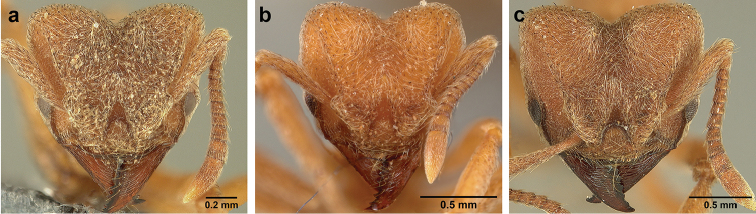
*S. saussurei*, *S. amabilis*, and *S. mayri*. **a** *S. saussurei* head, full-face view **b** *S. amabilis* head, full-face view **c** *S. mayri* head, full-face view.

### Species accounts

#### Sericomyrmex amabilis

Taxon classificationAnimaliaHymenopteraFormicidae

Wheeler, 1925b

**Figure 17.**
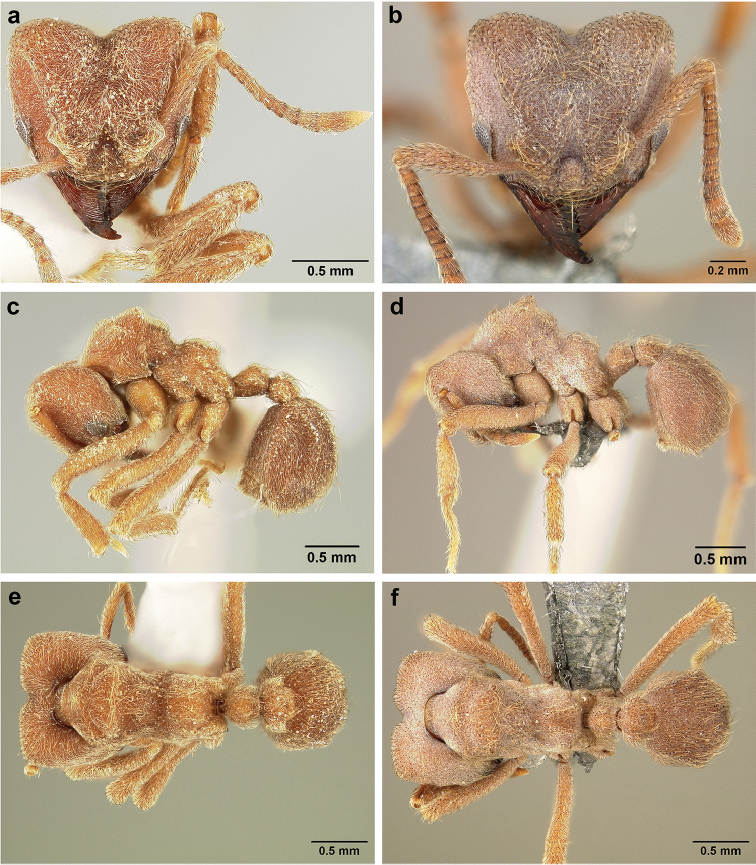
*S. amabilis* worker; head, lateral profile, and dorsal view. Striate-mandibled form (USNMENT00308236) (**a, c, e**) Smooth-mandibled form (USNMENT01125194) (**b, d, f**).

**Figure 18.**
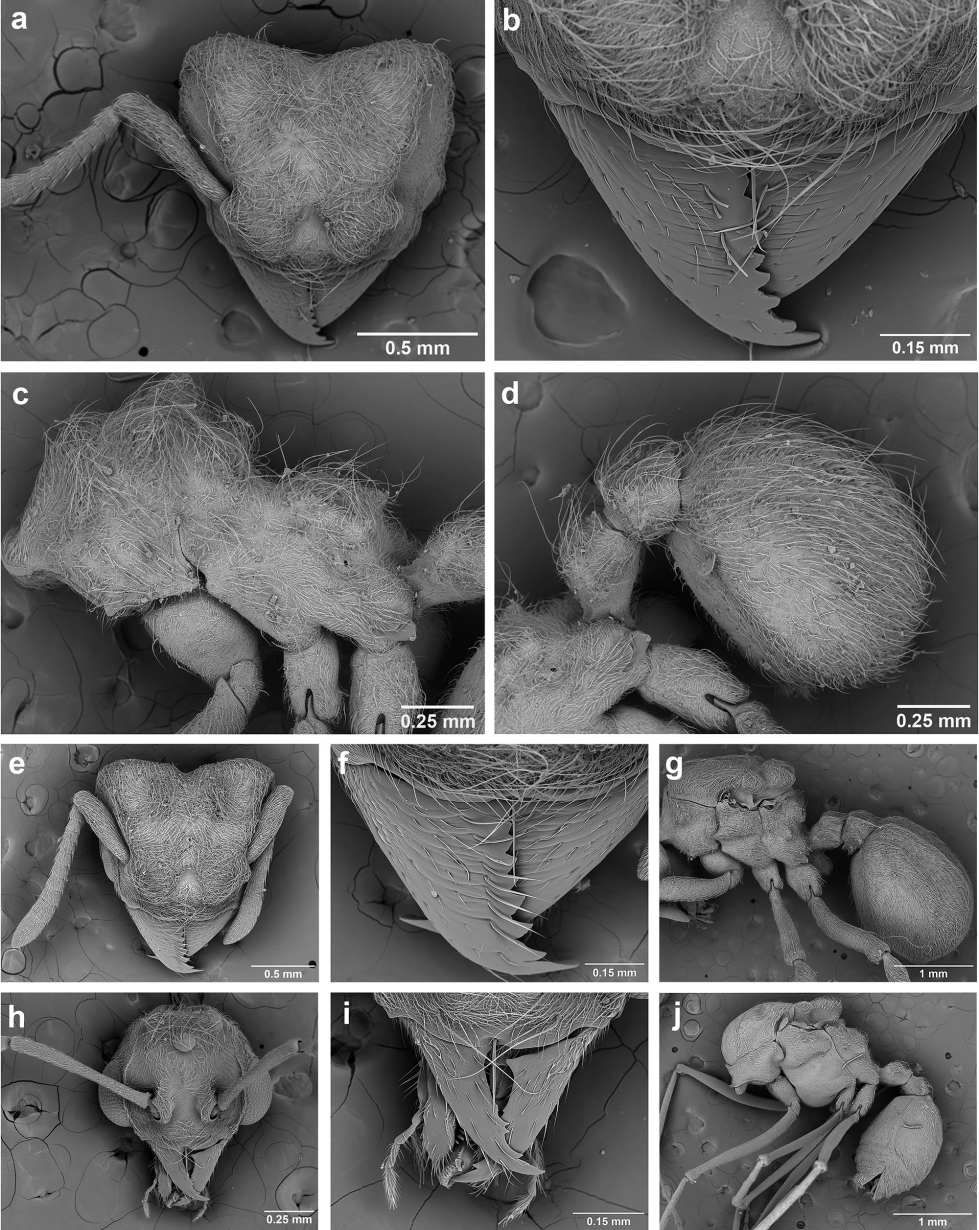
*S. amabilis* worker, queen, and male, SEM images. Worker (USNMENT01125862): **a** head, full-face view **b** mandibles **c** mesosoma, lateral view **d** metasoma, lateral view. Queen (USNMENT01125864): **e** head, full-face view **f** mandibles **g** mesosoma and metasoma, lateral view. Male (USNMENT01125863): **h** head, full-face view **i** mandibles **j** mesosoma and metasoma, lateral view.

**Figure 19.**
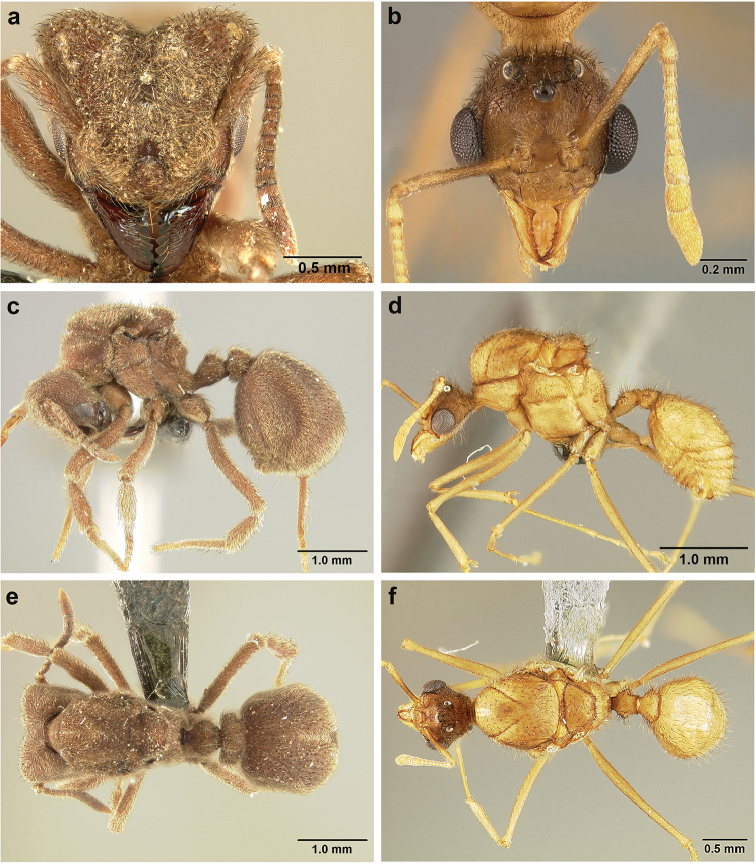
*S. amabilis* queen and male; head, profile, and dorsal view. Queen (USNMENT01125871) (**a, c, e**) Male (USNMENT01125846) (**b, d, f**).

**Figure 20.**
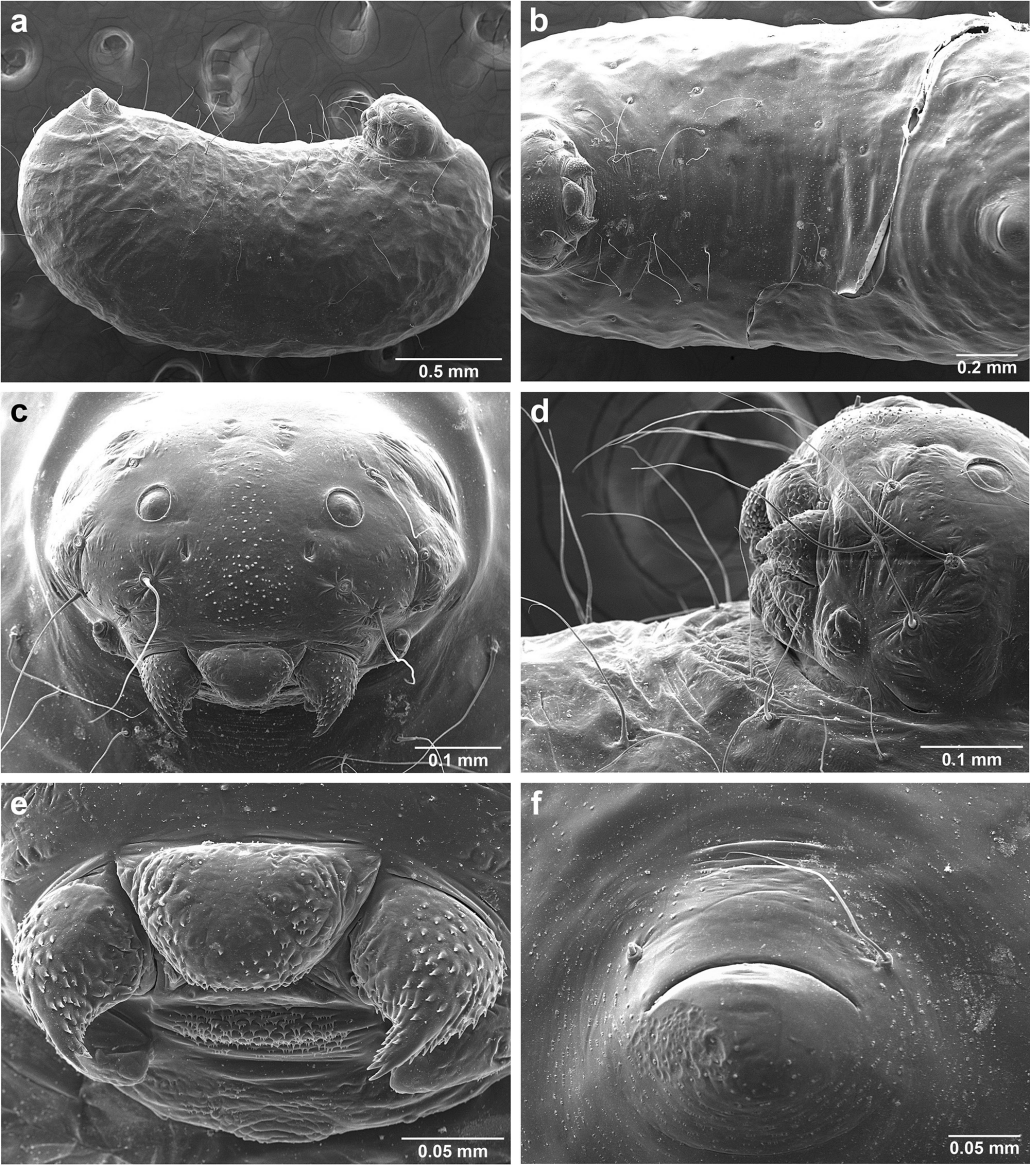
*S. amabilis* larva (USNMENT01126216), SEM images. **a** Lateral view **b** ventral view **c** head, frontodorsal view **d** head, lateral view **e** mouthparts; **f** anal setae.

**Figure 21.**
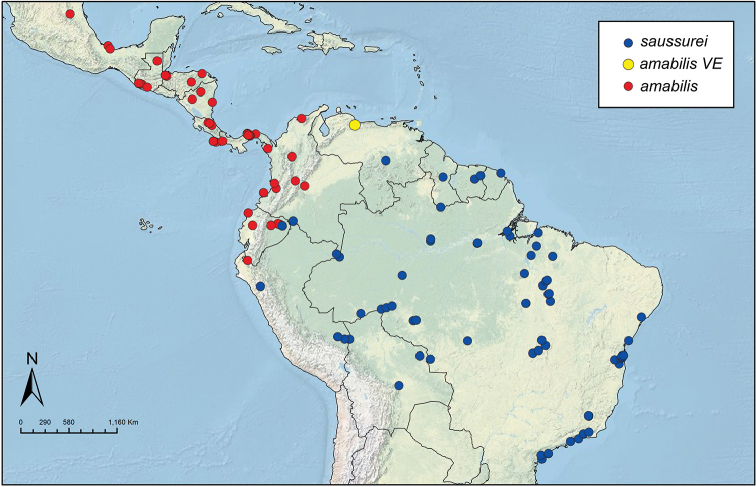
Distribution map of *S. amabilis* and *S. saussurei*.

Figures 17 , 18a–d (Worker); Figures 18e–j , 19 (Queen and male); Figure 20 (Larva); Figure 21 (Map)

Sericomyrmex amabilis Wheeler, 1925b: 166. Lectotype worker (here designated): PANAMÁ, Panamá, Barro Colorado Island, [9.1543, -79.8461], 3 Aug 1924, W. M. Wheeler, WMW838 (USNM: 3w, USNMENT00920034, topmost specimen on the pin). *Paralectotypes*: Same data as lectotype (USNM: 2w, USNMENT00920034, lower two specimens on the pin) (MCZ: 2w, 1q, MCZ40-42 21197).
Sericomyrmex bierigi = Santschi, 1931: 279. Type material examined: PANAMÁ, Chiriquí, La Concepción, 16 Jul 1930, Bierigi, (NHMB: 1w, CASENT0912515) [image examined]. Weber 1958: 263: junior synonym of amabilis.

##### *S. amabilis* worker diagnosis.

Medium-sized species; mandible usually striate; frontal carina complete; frontal lobe triangular; eye almost flat, without white layer; posterior cephalic margin with abrupt to gradual emargination; mesosomal tubercles from low and obtuse to well developed; first gastral tergite with lateral carinae strongly developed, dorsal carinae from weak to well developed.

***S. amabilis* worker description.** Measurements in mm, range (lectotype): HWe 0.88–1.21 (1.15) HW 0.88–1.24 (1.15) HW1 0.82–1.18 (1.12) HW2 0.92–1.36 (1.2) HW3 0.58–0.9 (0.75) IFW1 0.56–0.86 (0.78) IFW2 0.19–0.35 (0.3) HL1 0.82–1.2 (1.05) HL2 0.76–1.04 (0.93) SL 0.65–0.87 (0.76) EL 0.11–0.2 (0.162) Om 7–11 (8) WL 1.13–1.6 (1.44) PL 0.22–0.4 (0.3) PPL 0.16–0.29 (0.24) GL 0.78–1.12 (1.03) HFL 0.93–1.38 (1.21) PW 0.58–0.92 (0.78) CI 97–111 (110) FLI 60–72 (68) SI 66–78 (66) OI 12–19 (14) CEI 5–15 (10) [N=70]

***Pilosity.*** Pubescence dense, often lighter colored than integument, appressed to decumbent. Hairs curved, darker in color at base, yellow to gray, appressed to suberect, mostly decumbent.

***Head.*** In full-face view slightly broader than long (CI=104 ± 3, mean ± SD), posterior corner rounded to angular. Posterior cephalic margin with distinct median emargination (CEI=10 ± 2), gradually (Figure 7j) or abruptly (Figure 7k) impressed. Vertexal impression and frontal tumuli usually distinct. Mandible with 7–9 teeth, dorsally glossy and striate, striation sometimes reduced. Eye medium-sized (OI=16 ± 1), flat to slightly convex, 7–11 ommatidia across largest diameter. Frontal lobe triangular, posterior margin shorter than medial, lobe diverging laterally, relatively wide (FLI=68 ± 2). Frontal carina complete, reaching posterior cephalic corner. Antennal scape relatively short, never reaching posterior cephalic corner (SI=71 ± 3).

***Mesosoma.*** Mesosomal tubercles from low and obtuse to well developed. Propodeal carinae low, sometimes serrate, each with low posterodorsal denticle.

***Metasoma.*** Petiole and postpetiole each with pair of low, serrate carinae dorsally; in petiole sometimes reduced to two low denticles, seen in dorsolateral view. Postpetiole with another pair of low carinae laterally, sometimes reduced to low denticles. First gastral tergite with lateral carinae strongly developed, dorsal carinae faint in most specimens, sometimes strongly developed.

##### *S. amabilis* queen description.

Measurements in mm, range: HWe 1.27–1.40 HW 0.34–1.45 HW1 1.30–1.52 HW2 1.40–1.52 HW3 0.87–1.04 IFW1 0.90–1.00 IFW2 0.27–0.37 HL1 1.27–1.45 HL2 1.15–1.25 SL 0.81–0.99 EL 0.23–0.29 Om 15–22 EW 0.06–0.1 WL 1.95–2.2 PL 0.45–0.58 PPL 0.28–0.4 GL 1.68–1.87 HFL 1.16–1.58 PW 1.05–1.52 FWg 5.85–6.93 HWg 3.79–4.41 CI 93–104 FLI 70–77 SI 62–74 OI 18–22 [N=10]

***Head.*** Mandible with 8–9 teeth, dorsally glossy and striate. Preocular carina usually fading posterior to eye. Eye large (OI=20 ± 1), nearly flat, 15–22 ommatidia across largest diameter. Frontal lobe as in worker, antennal scape not reaching posterior cephalic corner.

***Mesosoma.*** Scutum in dorsal view with notauli weak, median mesoscutal sulcus reduced. Groove between axillae in dorsal view sometimes weakly transversely costate. Scutellum slightly convex in profile view, narrowing posteriorly in dorsal view, posterior margin medially with wide shallow V-shaped notch, notch sometimes continuing into median impression that divides scutellum into two lateral parts. Propodeum in dorsal view with two low carinae, each with posterodorsal denticle.

***Metasoma.*** First gastral tergite with lateral carinae well developed, dorsal carinae absent, anteromedian groove distinct.

##### *S. amabilis* male description.

Measurements in mm, range: HWe 0.61–0.98 HW 0.6–0.78 IFW1 0.24–0.48 IFW2 0.17–0.25 HL1 0.61–0.78 SL 0.59–0.76 EL 0.24 0.35 Om 20–26 EW 0.1–0.18 WL 1.43–2.02 PL 0.25–0.43 PPL 0.18–0.25 GL 1.08–1.75 HFL 1.48–1.95 PW 0.58–0.90 IOD 0.54–0.63 FWg 4.28–5.77 HWg 2.68–3.68 CI 100–129 FLI 33–60 SI 74–99 OI 34–40 [N=10]

Head in full-face view longer than broad (CI=120 ± 7). Eye large (OI=38 ± 2), 20–26 ommatidia across largest diameter. Preocular carina slightly curved medially, fading posterior to eye. In dorsal view, scutum with notauli well developed, mesoscutal line faint, groove between axillae with up to four transverse costae. Propodeal carinae short and faint. Petiole with lateral and dorsal denticles, postpetiole with very reduced lateral denticle.

##### *S. amabilis* larva description.

About 15 setae on each side of dorsal and lateral body surfaces (i.e., total ~30). Supra-antennal setae present. Four genal setae on each side. Mandibular apical tooth divided. Labial denticles present anterior to sericteries, sparse. First thoracic segment ventrally with multiple multidentate spinules, arranged in transverse rows. Numbers of ventral setae: six on T1, four on T2, four on T3, and around ten on abdomen (not including anal setae). Single pair of setae anterior to anal opening.

##### *S. amabilis* geographic range.

Central America, Colombia, Ecuador, Mexico, Venezuela. Map: Figure 21.

##### *S. amabilis* notes.

*S. amabilis* differs from the sympatric *S. opacus* by its larger size, striate mandibles (in *opacus* always smooth), triangular frontal lobes (rectangular in *opacus*), the shape of the posterior cephalic corners (in *opacus* smoothly rounded), and the presence of dorsal carinae on the gaster. The differences in size and mandibles also help in separating queens of these two species. In addition, the notauli and mesoscutal line are often pronounced in *amabilis* and faint or absent in *opacus*, although this can vary in both *amabilis* and *opacus*.

*S. amabilis* can be separated from its sister species *S. saussurei* by its flatter, uncoated eyes (convex, with a thick white layer in *saussurei*) and usually by geography (Figure 21). It can be separated from *S. mayri* by its smaller size, narrower head, complete frontal carinae, and wider frontal lobes.

Within *S. amabilis*, there is variation in the character of smooth versus striate mandibles. In the majority of specimens examined for this revision (including the type specimens) the mandibles are consistently striate across the entire dorsal surface (Figure 17a). In some populations, however, some specimens from a given locality have striate mandibles and some have completely smooth mandibles (Figure 17b). In those populations intermediate forms are also found, with faint striations most obvious along the posterior lateral edge of the mandible.

The population of *amabilis* at Costa Rica, Heredia, La Selva Biological Station, exemplifies the polymorphic state in which both smooth and striate mandibles co-occur, whereas the population from Costa Rica, Puntarenas, Osa Peninsula, contains only the striate state. A few specimens from Nicaragua and Ecuador also have smooth or faintly striate mandibles. Specimens of *amabilis* from Gorgona National Park (an island 35 km off the Pacific coast in the department of Cauca, Colombia) show the complete range of variation, from fully striate through intermediate to completely smooth mandibles. We did not find both forms, fully striate and completely smooth, co-occurring within a single nest, but we examined nest series from only a few localities. The smooth-mandibled populations do not form distinct clusters in molecular phylogenies of *amabilis*; rather, smooth-mandibled specimens group with striate-mandibled ones and vice versa. Likewise, statistical analyses of morphological measurements do not identify any distinct clusters correlated with mandibular sculpture (Figure 5e).

Members of the Gorgona population of *amabilis* have longer and sharper lateral mesonotal tubercles and more robust anteromedian dorsal carinae on the gaster compared to Central American populations. Some specimens from Nicaragua have a thin, translucent white layer covering part of the eye, similar to the condition observed in *S. opacus*. Other variable characters in *amabilis* are the frontal carinae (usually complete, but less developed in some), head shape (from angular to more rounded posterior head corners), and the size of the frontal lobes.

A single sequenced specimen from the population of *S. amabilis* from Venezuela (indicated with a yellow circle in the Figure 21) is molecularly distinct from the rest of the *amabilis* clade, as indicated by its position in the molecular phylogeny of *Sericomyrmex* (Suppl. material 1). This specimen, referred to as *S. amabilis* VE, is the sister of a clade that contains two reciprocally monophyletic subclades: (i) the monophyletic *S. saussurei* and (ii) all *S. amabilis* except *S. amabilis* VE. The molecular phylogeny, based on UCEs, reconstructs *S. amabilis* VE as a separate, third, species-level lineage (Ješovnik et al, 2017). The two available specimens, however, are morphologically identical to the specimens assigned to *amabilis*. Because of the small number of specimens and the lack of distinguishing morphological data, and because the molecular data are consistent with a scenario in which *S. saussurei* arises from within a paraphyletic *S. amabilis*, we are for now treating *S. amabilis* VZ as an allopatric population of *amabilis* that renders *amabilis* paraphyletic. We would like to encourage further study of this population and more thorough sampling in Venezuela in general, which will hopefully bring more insight into the species status of this population.

##### *S. amabilis* material examined.

**COLOMBIA**: **Antioquia**: Amalfi Cañon del Río Porce, El Caiman, 6.8572, -75.0958, 970m, 19 Dec 1999, E. Vergara, F. Serna; **Cauca**: PNN Gorgona Alto El Mirador, 2.9666, -78.1833, 180m, 21 Oct 2000, R. Duque; PNN Gorgona Antigua Laguna, 2.9666, -78.1833, 70m, 20 Dec 2000, R. Duque; PNN Gorgona El Helechal, 2.966, -78.1833, 30m, 17 Mar 2002, R. Duque; PNN Gorgona El Roble, 2.9666, -78.1833, 120m, 17 Jun 2001, R. Duque; PNN Gorgona El Samán, 2.9666, -78.1833, 5m, 14 Sep 2001, H. Torres ; PNN Gorgona Mancora, 2.9666, -78.1833, 60m, 20 Dec 2000, R. Duque; **Cundinamarca**: Melgar to Girardot, [4.249, -74.726], 28 Mar 1967, R. B. Root, W. L. Brown; **Magdalena**: 4 km N San Pedro, 10.95, -74.05, 220m, 14 Aug 1985, P. S. Ward; **Meta**: San Martín Caduceo, [3.6970, -73.6982], 400m, 4 May 2006, J. Ordonez; **Valle del Cauca**: Buenaventura, Bajo Calima, Villa Clara, [3.996, -76.974 ±2000m], 30m, 18 Mar 1967, R. B. Root, W. L. Brown; PNN Farallones de Cali, Anchicayá, 3.4333, -76.8, 730m, 1 Jul 2000, S. Sarria; **COSTA RICA**: **Heredia**: La Selva Biol. Station, 10.43, -84.017, 50-150m, 1 Aug 2004, J. Sosa-Calvo; **Limón**: Puerto Viejo, [10.51, -84.18], 18 Jun 1979, J. Paich; R. Toro Amarillo Vic., Guapiles, 10.2166, -83.7667, 25 Feb 1966, W. L. Brown; **Puntarenas**: 15 km SSW Puerto Jiménez, [8.4083, -83.3278 ±30m], 170m, 7 Mar 2010, J. T. Longino; Osa Peninsula, Corcovado, Sirena Station, 8.50, -83.6166, 31 May 1992, T. R. Schultz; **ECUADOR: Esmeraldas**: Las Vegas, 0.7547, -79.8490, 26 Jun 2003, A. G. Himler; **Loja**: Reserva Jorupe, Macará, -4.372, -79.906, 600m, 15 Sep 2011, T. Delsinne; **Los Ríos**: Río Palenque, 2km SSE Patricia Pilar, -0.5833, -79.3666, 160m, 1 Sep 1991, M. J. Stern; Río Palenque Research Station, 47 km S Santo Domingo [-0.583, -79.367], 28 May 1905, S., J. Peck; **Napo**: Limoncocha, -0.4, -76.6, 250m, 18 Jun 1976, S., J. Peck; Tiputini, La Selva, Chorongo trail, -0.4975, -76.3747, 12 Jun 2003, A. Little; **GUATEMALA: Escuintla**: Escuintla, [14.2445, -90.7995], 22 Sep 2008, W. M. Mann; **Izabal**: 16 km ESE Morales, 15.4111, -88.7118 ±58m, 440m, 19 May 2009, J. T. Longino; 5 km NW Morales, 15.5097, -88.8627 ±36m, 160m, 18 May 2009, J. T. Longino; **Petén**: Cerro Cahui, 17.0023, -89.7194, 210m, 3 Feb 2009, Llama team; **Retalhuleu**: El Asintal, 14.6555, -91.7344, 720m, 19 Feb 2013, K. Delgado; Nuevo San Carlos, 110 km NW Retalhuleu, 14.6258, -91.7234, 440m, 27 Feb 2013, K. Delgado; San Felipe, 14.6309, -91.581, 745m, 8 Feb 2013, K. Delgado; **HONDURAS: Gracias a Dios**: Las Marias, 15.6817, -84.8352 ±20m, 80m, Llama team; **Olancho**: 14 km WSW Catacamas, 14.7997, -86.0141 ±210m, 600m, 13 May 2009, J. T. Longino; **MEXICO: San Luis Potosí**: Río Santa Maria, Tamul, 21.8025, -99.1803, 200m, 2 Jul 1992, S. Sanchez-Pena; **Veracruz**: Catemaco, Tuxtla, 18.5865, -95.0779, 184m, 3 Oct 2013, A. Ješovnik; Ocotal Chico, 18.2588, -94.8619, G. N. Ross; **NICARAGUA: Matagalpa**: Pancasan, nr. Río Guapotal, 12.92, -85.55, 450m, 17 Jun 1992, T. R. Schultz, J. C. Gomez; Selva Negra, ca. 12 km N Matagalpa, Reserva Natural Cerro El Arenal, 12.9812, -85.9136, 1009m, 28 Dec 2007, C. Rabeling; **Región Autónoma del Atlántico Norte**: PN Cerro Saslaya, 13.7705, -84.9789 ±20m, 290m, 7 May 2011, M. G. Branstetter; **Región Autónoma del Atlántico Sur**: RN Kahka Creek, 12.6851, -83.7136, 50m, 8 Jun 2011, M. G. Branstetter; **PANAMÁ: Colón**: Fort Sherman, 9.36, -79.95, 28 Apr 1996, T. R. Schultz, U. G. Mueller, S. Rehner; Gamboa, PN Soberanía, Pipeline Rd. ca. 2 km past Río Frijoles, 9.1205, -79.7067, 54m, 25 May 2002, C. J. Marshall; Gatún, Punta da los Chivos, 3 km SW Gatún, [9.26, -79.91], 1 Jul 1979, W. L. Brown; Mt. Hope, nr. Colón, 9.2833, -79.9667, 24 Jul 1924, W. M. Wheeler; San Lorenzo Forest, 9.2833, -79.9666, 25 May 2004, R. K. Didham; **Darién**: Cana, 7.7166, -77.7, 800m, 23 Aug 1987, D. M. Olson; **Panamá**: Barro Colorado Island, 9.15, -79.84, 1 Aug 1946, J. Zetek; Gamboa, Pipeline Rd, ca. 2 km past Río Frijoles, 9.1205, -79.7066, 54m, 25 May 2002, C. J. Marshall; Nusagandi Biol. Stn, Markisgandi trail, [9.2848, -79.0280], 26 Apr 1996, T. R. Schultz; PN Soberanía, Plantation Rd., 9.08, -79.66, 4 May 2011, R. M. M. Adams; **VENEZUELA: Carabobo**: Mocundo, ca. Aguirre, [10.2533, -68.2683], 750m, 27 Dec 2010, J. Lattke.

#### Sericomyrmex bondari

Taxon classificationAnimaliaHymenopteraFormicidae

Borgmeier, 1937

**Figure 22.**
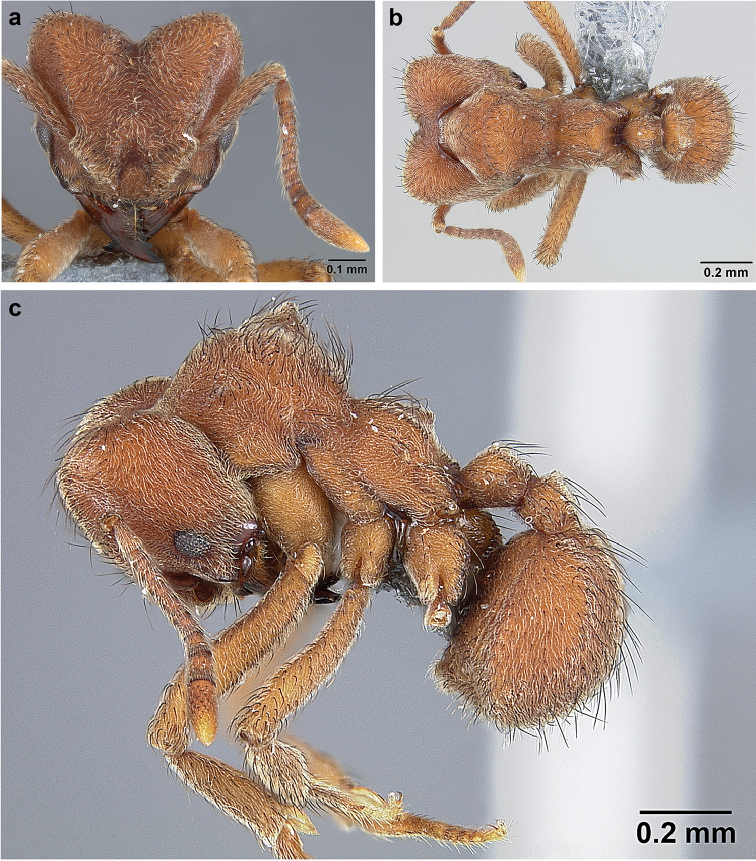
*S. bondari* worker (USNMENT01125207). **a** Head **b** dorsal view; and **c** lateral profile.

**Figure 23.**
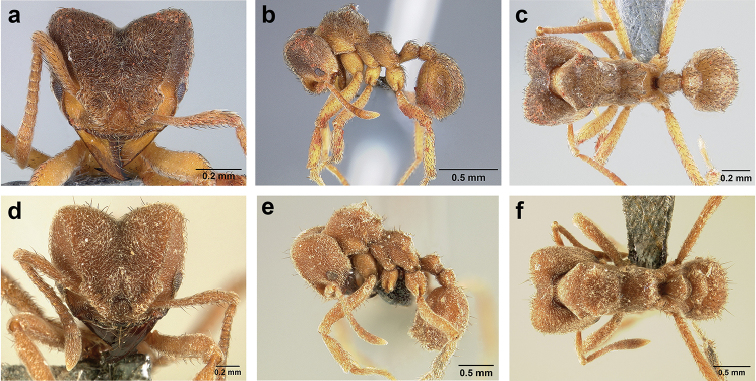
*S. bondari* worker variation. Worker with bicolored integument (USNMENT01125207) (**a, b, c**). Worker with reduced hairs (USNMENT01125823) (**d, e, f**).

**Figure 24.**
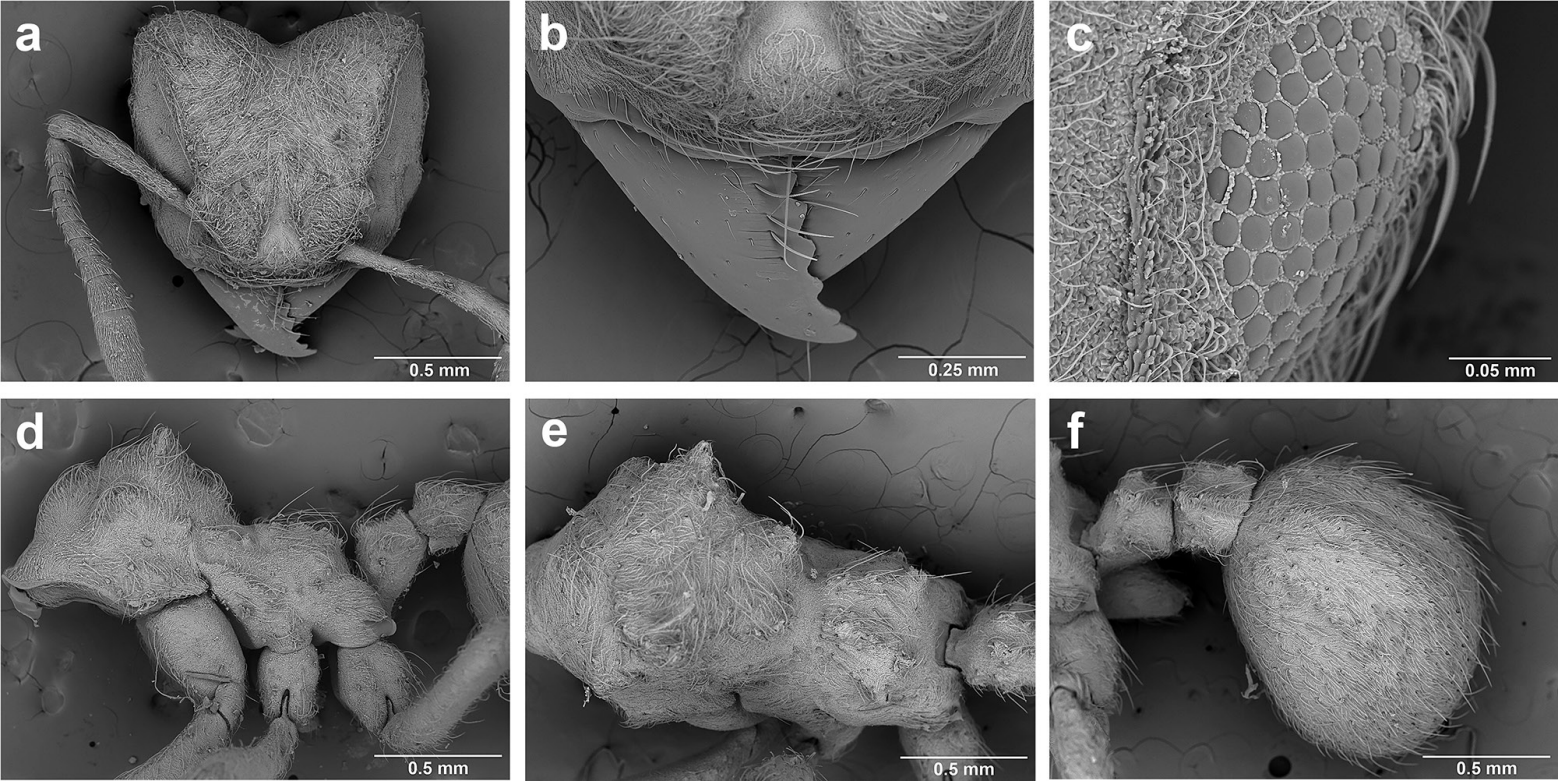
*S. bondari* worker (USNMENT01125813), SEM images. **a** Head, full-face view **b** mandibles **c** eye **d** mesosoma, lateral view **e** mesosoma, dorsal view **f** metasoma, dorsolateral view.

**Figure 25.**
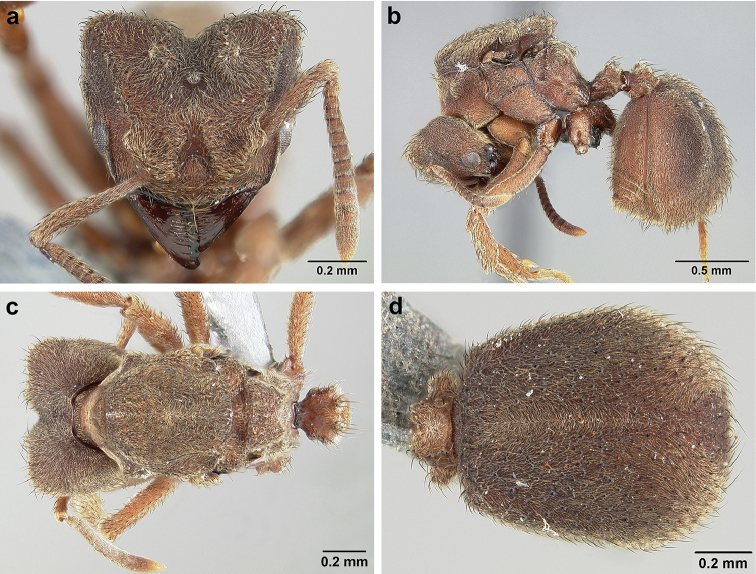
*S. bondari* queen (USNMENT01125803). **a** Head **b** lateral profile **c** mesosoma, dorsal view **d** metasoma, dorsal view.

**Figure 26.**
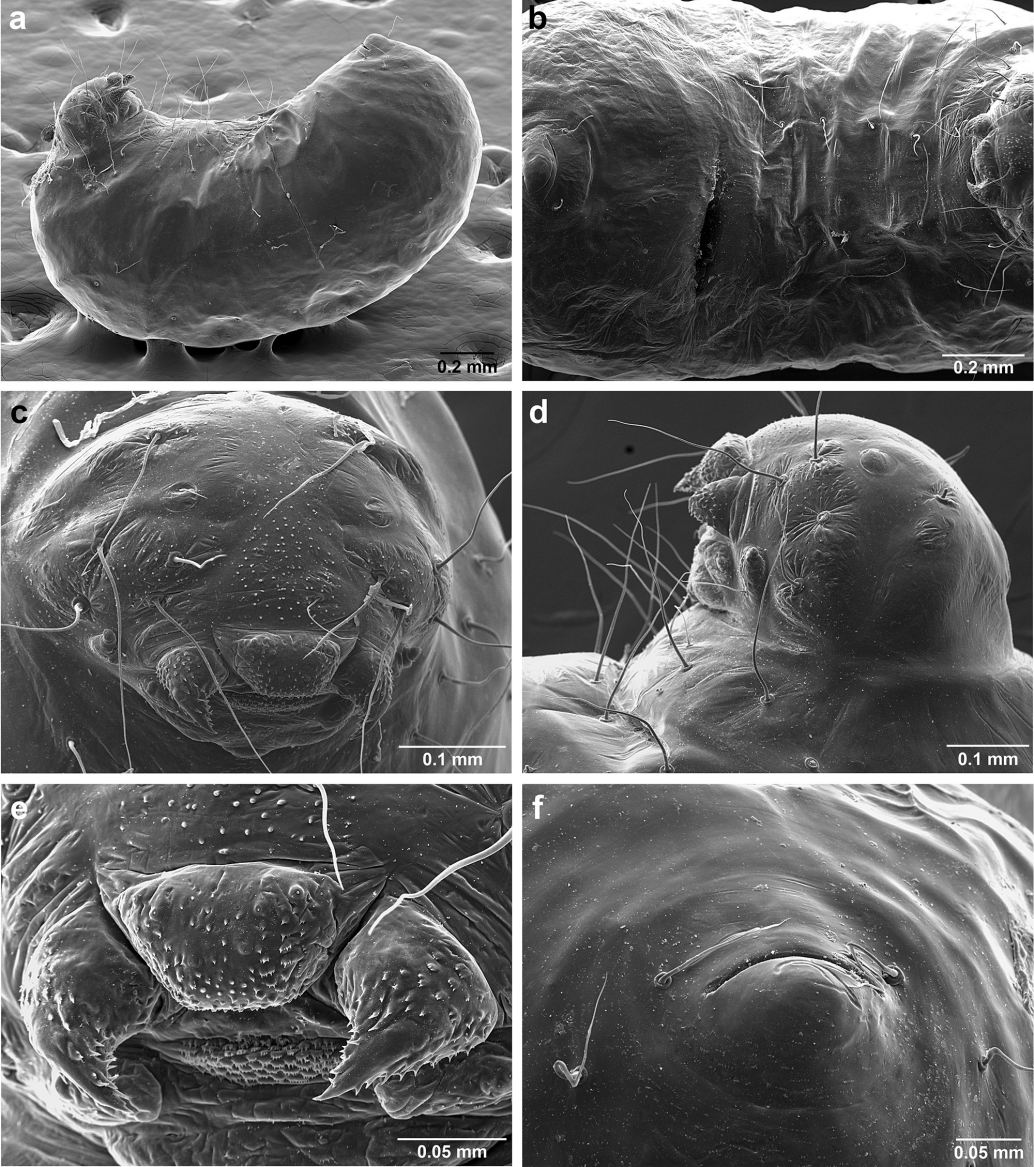
*S. bondari* larva (USNMENT01125807), SEM images. **a** Lateral view **b** ventral view **c** head, frontodorsal view **d** head, lateral view **e** mouthparts **f** anal setae.

**Figure 27.**
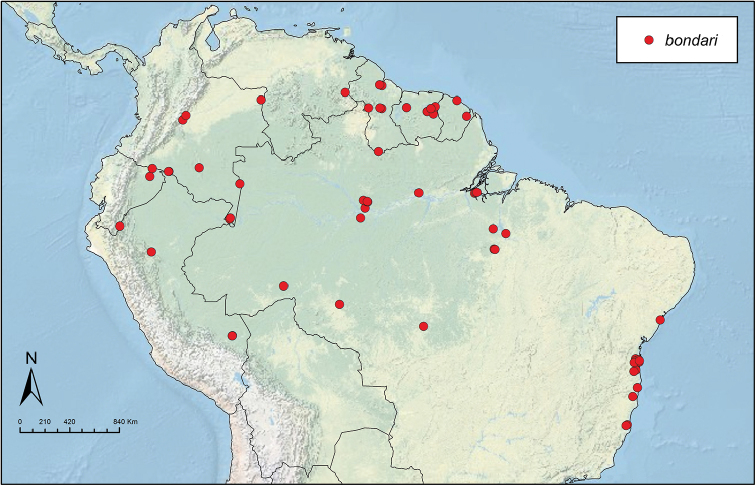
Distribution map of *S. bondari*.

Figures 22 , 23 , 24 (Worker); Figure 25 (Queen); Figure 26 (Larva); Figure 27 (Map)

Sericomyrmex bondari Borgmeier, 1937: 248. Lectotype worker (here designated): BRAZIL, Bahia, Sul da Bahia, Áqua Preta, [-8.8333, -66.1667], 1 May 1936, G. Bondar (MZSP: 3w; USNMENT01126238, topmost specimen on the pin). Paralectotypes: same data as lectotype (MZSP: 2w; USNMENT01126238, lower two specimens on the pin).
Sericomyrmex beniensis = Weber, 1938: 182. **syn. n.** Type material examined. BOLIVIA, Huachi Beni, 31 Dec 1921, W. M. Mann, WMM327. (USNM: 3w, USNMENT01126217; 3w, USNMENT01126218; 3w, USNMENT01126220; 3w, USNMENT01126221) (MCZ: 3w, USNMENT01126219).

##### *S. bondari* worker diagnosis.

Large species; hairs thick and dark; posterior cephalic emargination deep, gradually impressed; posterior cephalic corner acute to rounded; mandible dorsally smooth, glossy; frontal lobe triangular, narrow; mesosomal tubercles distinct, sometimes relatively sharp; first gastral tergite with lateral carinae weakly to moderately developed, dorsal carinae absent.

##### *S. bondari* worker description.

Measurements in mm, range (lectotype): HWe 0.96–1.40 (1.16) HW 0.98–1.40 (1.18) HW1 0.93–1.40 (1.2) HW2 1–1.56 (1.24) HW3 0.65–0.9 IFW1 0.65–1.00 (0.76) IFW2 0.22–0.38 (0.3) HL1 0.99–1.30 (1.01) HL2 0.87–1.12 (0.93) SL 0.68–0.96 (0.78) EL 0.14–0.20 (0.16) Om 8–12 (11) WL 1.25–1.76 (1.52) PL 0.24–0.4 (0.38) PPL 0.18–0.3 (0.19) GL 0.81–1.24 (0.96) HFL 1.12–1.52 (1.3) PW 0.66–1.02 (0.78) (0.8) CI 93–110 (107) FLI 61–72 (66) SI 63–78 (67) OI 12–17 (14) CEI 6–17 (13) [N=59]

***Pilosity.*** Hairs thick, black, often curved, appressed to erect, mostly suberect, longer and denser on dorsal than on lateral surfaces, e.g., mesosoma laterally with barely any hairs, just pubescence.

***Head.*** In full-face view slightly broader than long (CI=104 ± 3), posterior corner rounded to acute, posterior cephalic emargination distinct and deep (CEI=13 ± 2), gradually impressed. Vertexal impression often distinct and deep, sometimes extending anterad to include frons, frontal tumuli usually faint. Mandible with 7–9 teeth, dorsally smooth, glossy, finely transversely striate along masticatory margin, striation sometimes faint. Eyes medium-sized (OI =15 ± 1), mildly convex, lacking white layer, 8–12 ommatidia across largest diameter. Frontal lobe triangular, narrow (FLI=67 ± 2), posterior margin shorter than medial, and with long lateral margin, giving lobe appearance of being directed anterad. Frontal carina complete, reaching posterior cephalic corner, antennal scape relatively short (SI=71 ± 3), not reaching posterior cephalic corner. Antennal scape with thick black hairs, antennal flagellum lacking thick black hairs, but with pubescence and thin, long, light yellow hairs.

***Mesosoma.*** Lateral pronotal and lateral mesonotal tubercles from moderately developed to large and sharp, variable within species and within colonies. Propodeal carinae low, serrate, each with posterodorsal denticle.

***Metasoma.*** Petiole with two low denticles dorsally, postpetiole with two dorsal and two lateral carinae, lateral pair sometimes faint. First gastral tergite with lateral carinae distinct, dorsal carinae faint or absent. Decumbent to suberect hairs on gastral dorsum curved at base and sometimes hooked at tip, margins of gastral segments 2–5 (A5-A8) with curved suberect to erect hairs.

##### *S. bondari* queen description.

Measurements in mm, range: HWe 1.33–1.48 HW 1.35–1.48 HW1 1.40–1.46 HW2 1.15–1.58 HW3 0.93–1.09 IFW1 0.99–1.02 IFW2 0.34–0.40 HL1 1.32–1.44 HL2 1.12–1.24 SL 0.92–1.00 EL 0.27–0.29 Om 17–21 EW 0.08–0.08 WL 1.96–2.25 PL 0.40–0.45 PPL 0.24–0.28 GL 1.95–2.05 HFL 1.60–1.64 PW 1.15–1.28 FWg 6.71–6.71 HWg 4.66–4.66 CI 98–103 FLI 69–75 SI 68–71 OI 19–22 [N=3]

***Head.*** Mandible with 7–8 teeth, dorsally smooth, glossy, finely transversely striate only along masticatory margin. Preocular carina fading posterior to eye, several short and thin supraocular carinae present, never reaching posterior cephalic corner (Figure 8c). Eye large (OI=20 ± 1) and convex, 17–21 ommatidia across largest diameter. Frontal lobe wider than in worker (FLI=73 ± 3), antennal scape as in worker, not reaching posterior cephalic corner.

***Mesosoma.*** Lateral pronotal tubercles distinct. Scutum in dorsal view with notauli faint, sometimes absent. Median mesoscutal line sometimes anteriorly developed into weak costa, posteriorly with shallow longitudinal impressions on each side. Parapsidal lines thin, slightly curved. Scutellum inflated, short in dorsal view, narrowing posteriorly, posterior margin with V-shaped, relatively deep, medial notch; notch sometimes continuing into median impression that divides scutellum in two lateral parts. Propodeal carinae short, low, each with posterodorsal denticle, sometimes carinae reduced and only denticles visible.

***Metasoma.*** Petiole in frontodorsal view with two narrow, long dorsal denticles and two smaller, lateral denticles. Postpetiole with two short and low dorsal carinae and two low lateral denticles. First gastral tergite with lateral carinae strongly developed, dorsal carinae absent or faint, anteromedian groove distinct.

##### *S. bondari* male.

Unknown.

##### *S. bondari* larva description.

Two to four setae on dorsal and lateral body surfaces on each side. Supra-antennal setae present. Four genal setae on each side. Mandibular apical tooth undivided. Small number of labial denticles anterior to sericteries. First and second thoracic segments ventrally with multiple multidentate spinules, arranged in transverse rows. Numbers of ventral setae: six on T1 and T3, four on T2, around six on abdomen (not including anal setae). One pair of setae directly anterior to anal opening, another pair on abdominal segment 9 laterad of anal opening.

##### *S. bondari* geographic range.

Brazil, Bolivia, Colombia, Ecuador, French Guiana, Guyana, Peru, Suriname. Map: Figure 27.

##### *S. bondari* notes.

It is difficult to mistake *S. bondari* for any other *Sericomyrmex* species because of its thick, dark hairs. When individuals with reduced hairs are encountered (Figure 23d–f), the sympatric sister species, *S. mayri*, can be separated from *bondari* by its striate mandibles, wider head (CI *mayri*=108, CI *bondari*=104), narrower frontal lobes, and shallow posterior cephalic emargination. The morphologically similar *S. radioheadi* is smaller (medium-sized, while *bondari* is large); completely lacks dark hairs; and has a longer antennal scape (SI=77), reduced lateral pronotal tubercles, and unusually sharp, long lateral mesonotal tubercles. The thick, dark hairs and large size are also useful for separating *S. bondari* queens from those of *mayri*, as are the lateral pronotal tubercles, which are more pronounced in *bondari* than in queens of other *Sericomyrmex* species.

The specimens of *S. bondari* with slightly to very reduced hairs include five workers from Brazil (Melgaço, Pará), one from Ecuador (Cuyabeno), and three from Venezuela (Bolívar) out of a total of ~200 dry-mounted specimens we examined. Aside from reduced hairs they have the typical *bondari* morphology and measurements (Figure 5g). Individuals with an intermediate state (hairs slightly reduced) exist in Brazil, some from Melgaço (Pará) and some from Espírito Santo. The existence of intermediate forms, the molecular phylogeny, and PCA analysis of the morphological measurement data all indicate that populations with reduced hairs are rare variants of *bondari* rather than separate species. Another variation within *bondari*, occurring in the Brazil (Carajás, Pará) specimens, is a slightly bicolored (rather than uniformly colored) integument (Figure 23a–c), but this state occurs only in a minority of workers from the same nest, the rest of which are homogeneously colored.

*Synonymy*. The specimens we examined from the type series of *S. beniensis*, collected by Mann in Bolivia and described by Weber (1938), are morphologically identical to the lectotype of *S. bondari*. In his description Weber compares it to *saussurei* and *bierigi* (=*amabilis*), but does not mention *S. bondari*, so he might not have seen Borgmeier’s specimens or description. Interestingly, Weber’s description mentions some important characters such as head shape, deep cephalic margin, and smooth mandibles, but not the dark hairs that make this species so easy to recognize.

##### *S. bondari* material examined.

**BRAZIL: Amazonas**: Manaquiri, Br 319, km101, [-3.68, -60.31], 10 Oct 2010, F. Baccaro; Manaus, Camp 41, -2.4494, -59.7634, 118m, 10 Jan 2009, J. Sosa-Calvo; Manaus, Reserva Ducke, -2.9324, -59.9721, 95m, 26 Sep 2012, A. Ješovnik; Manaus, BDFFP Camp Gaviao, -2.4219, -59.8469, 29 Feb 2000, T. R. Schultz; Manaus, BDFFP Dimona Camp, 100-ha. Frag., -2.3388, -60.1026, 16 Aug 2000, R. M. M. Adams; ZF3- Km41, -2.4166, -59.8, 20 Sep 1996, A. C. Macedo; **Bahia**: Canavieiras, Oiticica, -14.4094, -30.0166, 30 Mar 1998, J. S. C. Carmo; Ilhéus, Pimenteira mata W-A17, -14.5352, -39.4275, 6 Oct 1997, J. R. M. Santos, J. S. C. Carmo; Ilhéus, Ponta do Ramo, -14.4977, -39.0405, 11 Feb 1997, J. R. M. Santos; Itacaré, -14.3177, -39.0719, 3 Aug 1998, J. R. M. Santos; Itamaraju, [-17.0438, -39.5300], 29 Mar 2004, J. H. C. Delabie; Jussari, Pratos, -15.1955, -39.4452, 18 Jul 1997, J. R. M. Santos; Maraú-Trembebé Mata WA4, -14.4022, -39.3233, 7 May 1997, J. R. M. Santos; Porto Seguro, E.E. Pau Brasil, -16.3925, -39.1694, 16 Jun 2000, J. R. M. Santos, S. M. Soares; Ubaitaba, -14.2502, -39.3213, 9 Apr 1998, J. R. M. Santos; Unacau A43, -15.0891, -39.295, 11 Feb 2000, J. R. M. Santos; Uruçuca, Mata A19, -14.5125, -39.2002, 24 Oct 2002, J. R. M. Santos; **Espírito Santo**: Guriri, -18.7167, -39.75, 1 Mar 2005, M. C. Teixeira; Parque Sooretama, Linhares, -19.0725, -39.9491, 17 Oct 1962, F. S. Pereira; **Mato Grosso**: Sinop, [-11.8581, -55.5056], 1 Oct 1974, Alv., Roppa; **Pará**: Melgaço, Caxiuanã, ECFPn V Transecto 3-700) Winkler #2, -1.7248, -51.4230, 26 Apr 2004, A. Y. Harada; Nova Ipixuna, Fazenda Bom Retiro, Parcela 04, -4.8412, -49.2180, 12 Apr 2012, M. Tavares, A. Palmeira; Novo Repartimento, Faz. Arataú, [-4.49, -50.19], 17 Jun 2002, A. M. Elizabeth; Oriximiná, Alega Reloiado, -1.76, -55.85, 8 Oct 1982, A. Y. Harada; Parauapebas, FL Nacional de Carajás, Parque Zoobotânico, -6.0629, -50.0571, 626m, 1 Oct 2014, A. Ješovnik; **Rondônia**: Ouro Preto do Oeste, Res do INPA No 0158, [-10.2, -61.9], 27 Mar 1985, W. Franca; **Sergipe**: Santa Luzia do Itanhy, Crasto, -11.3775, -37.4187, 21 Jun 2001, R. R. Silva, R. M. Feitosa, C. R. F Brandão; **COLOMBIA: Amazonas**: PNN Amacayacu Matamata, -3.6833, -70.25, 150m, 1 Oct 2001, D. Chota; PNN Amacayacu, San Martín, -3.7666, -70.3, 150m, 7 Nov 2001, D. Chota; **Caquetá**: Puerto Solano, PNN Serranía de Chiribiquete, Río Sararamano, 0.167, -72.6097, 250m, 1 Apr 2000, E. Gonzales; **Meta**: PNN Sumapaz, Cabaña las Marías, 3.8, -73.8666, 779m, 1 Oct 2003, H. Vargas; Villavicencio, La Vanguardia, Sector Pozo Azul, [4.1451, -73.6269], 375m, 16 Apr 2005; **Putumayo**: PNN La Paya Cabaña Chagra, -0.1166, -74.9333, 320m, 15 Oct 2001, R. Cobete; **Vaupés**: Est. Biol. Mosiro-Itajura (Caparu) Centro Ambiental, -1.0666, -69.5166, 60m, 1 Feb 2003, M. Sharkey, D. Arias; **Vichada**: Cumaribo, Cgto. Santa Rita, PNN El Tuparro, 5.3316, -67.8908, 135m, 8 Feb 2004, I. Quintero, E. Gonzales; **ECUADOR: Morona-Santiago**: Los Tayos, [-4.3, -78.67], 3 Aug 1976, Tjitte de Vries; **Sucumbíos**: Reserva Faunistico Cuyabeno, 0.1167, -76.1833, 1 Nov 1994, J. P. Caldwell; La Selva Lodge, Mandi Cocha, [-0.4973, -76.3747], 11 Jun 2003, S. Villamarin; **FRENCH GUIANA: Cayenne**: 10 km south Sinnamary, Paracou forest, 5.2808, -52.9465, 2006-2009; Nouragues Field Station, 4.09, -52.2, 1 Oct 2009; **GUYANA: Cuyuni-Mazaruni**: Mazaruni River, Forest Settlement, 6.3973, -58.6781, 1 Aug 1935, N. A. Weber; Oko R., Cuyuni trib., [6.4638, -58.8538], 22 Jun 1936, N. A. Weber; **Potaro-Siparuni**: Iwokrama For. Res. Whitewater Camp, 4.7168, -58.8333, 60m, 6 Nov 2002, J. S. LaPolla; Iwokrama, Kurapakari base Camp, [4.6698, -58.6854], 60m, 6 Apr 1996, T. R. Schultz; Paramakatoi, PK-Yawong Trail, [4.7167, -59.7], 704m, 16 Apr 1996, T. R. Schultz; **Upper Takutu-Upper Essequibo**: Acarai Mts., nr Romeo’s camp, 1.3833, -58.9333, 735m, 16 Oct 2006, T. R. Schultz, C. J. Marshall; **PERU: Madre de Dios**: Puerto Maldonado, Los Amigos Biol. Station, -12.5617, -70.0924, 276m, 20 Nov 2005, J. Sosa-Calvo; **San Martín**: Davidcillo, 30km NNE Tarapoto, -6.25, -76.25, 220m, 21 Aug 1986, P. S. Ward; **SURINAME: Brokopondo**: Maripaheuvel, near Dam on Sara creek, [4.67, -54.95], 1 Sep 1959, I. v. d. Drift; Poeroe man Kemisa, [4.67, -54.95], 1 Sep 1959, I. v. d. Drift; **Sipaliwini**: Bakhuis Mountains, 4.7451, -56.7832, 5 m, 11 Mar 2006, J. Sosa-Calvo; Lely Mountains, 4.2529, -54.7561, 619m, 26 Oct 2005, J. Sosa-Calvo; Nassau Mountains, 4.8172, -54.6067, 514m, 3 Nov 2005, J. Sosa-Calvo; **VENEZUELA: Bolívar**: via El Dorado-Santa Elena Km. 80, [5.89, -61.46], 300m, 26 Jun 1984, J. Lattke.

#### Sericomyrmex lutzi

Taxon classificationAnimaliaHymenopteraFormicidae

Wheeler, 1916

**Figure 28.**
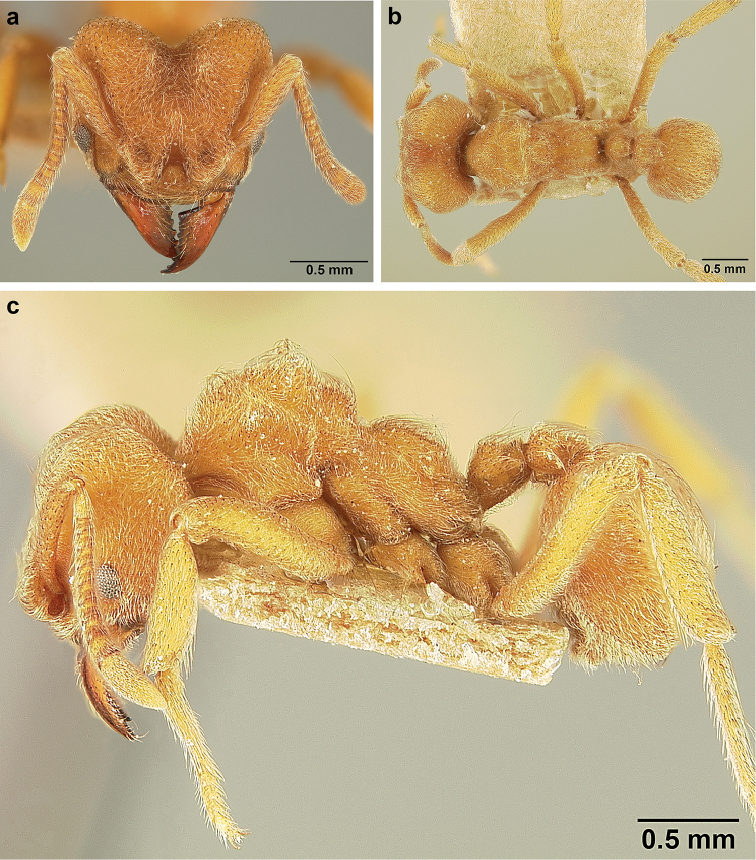
*S. lutzi* worker (MCZ 12-14 21139). **a** Head **b** dorsal view; and **c** lateral profile.

**Figure 29.**
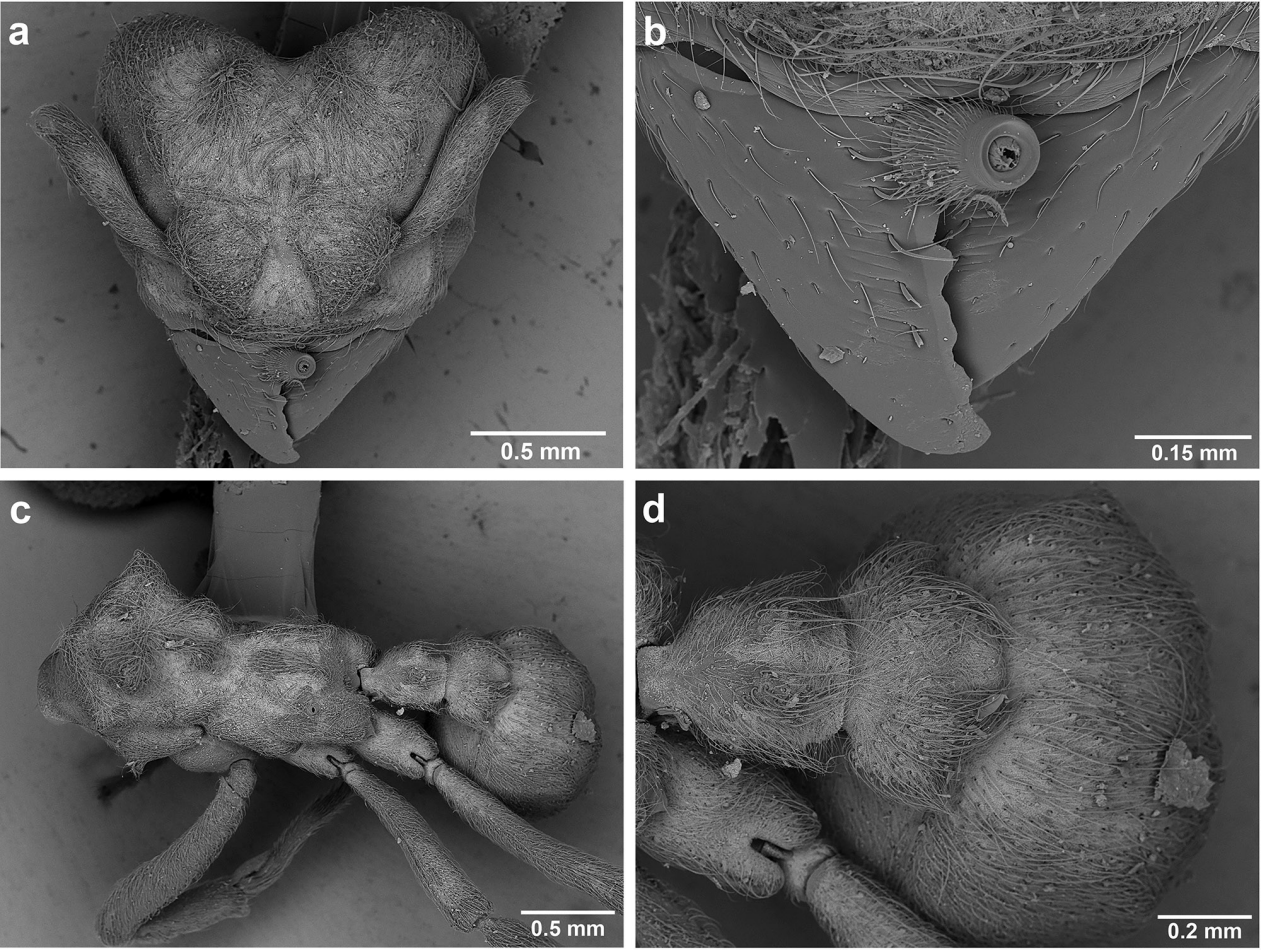
*S. lutzi* worker (USNMENT00445053), SEM images. **a** Head, full-face view **b** mandibles **c** mesosoma and metasoma, dorsolateral view **d** metasoma, dorsal view.

**Figure 30.**
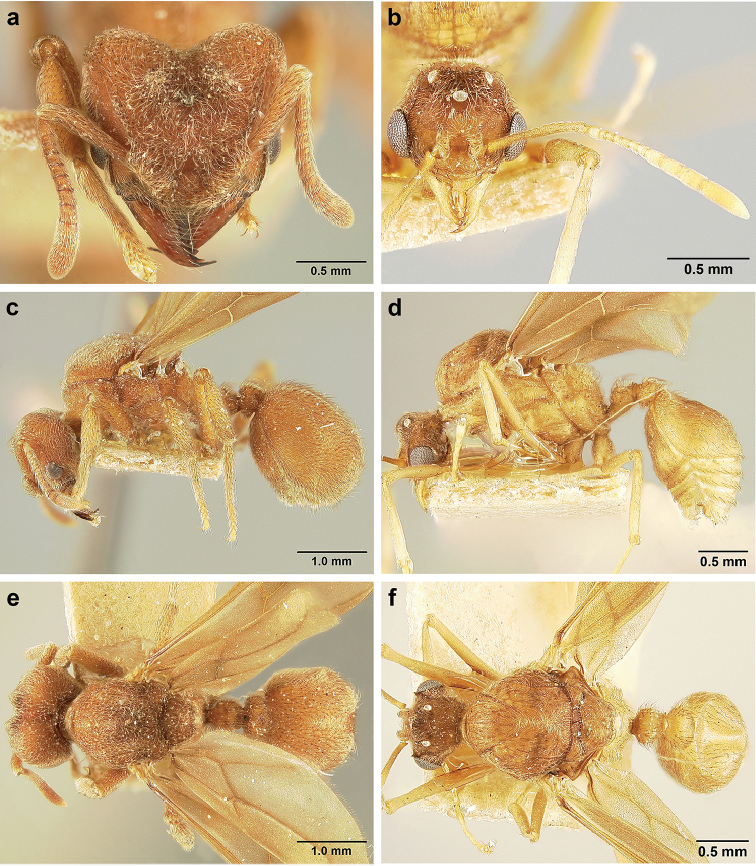
*S. lutzi* queen and male; head, lateral profile, and dorsal view. Queen (MCZ 9-11 21139) (**a, c, e**) Male (USNMENT01126226) (**b, d, f**).

**Figure 31.**
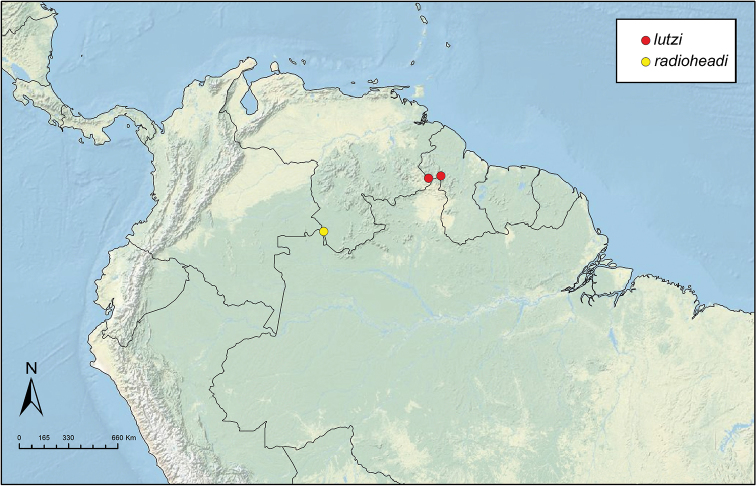
Distribution map of *S. lutzi* and *S. radioheadi*.

Figures 28 , 29 (Worker); Figure 30 (Queen and male); Figure 31 (Map)

Sericomyrmex lutzi Wheeler, 1916: 9. Lectotype worker (here designated): GUYANA, Roraima, Kauwa creek [5.223, -60.73], 13 Aug 1911, A. Crampton, AC3097 (MCZ: 1w, MCZ 12-14 21139, topmost worker on the pin). *Paralectotypes*: same data as lectotype (MCZ: 2w, MCZ 12-14 21139, lower two specimens on the pin) (MCZ: 2w, 1q, MCZ 9-11 21139) (AMNH: 1w, 1q, 1m, USNMENT01126226).

##### *S. lutzi* worker diagnosis.

Large species; posterior cephalic emargination deep, gradually impressed; mandible dorsally smooth and glossy; frontal lobe triangular, relatively narrow; mesosomal tubercles low; first gastral tergite with lateral carinae weak, dorsal carinae absent.

##### *S. lutzi* worker description.

Measurements in mm, range (lectotype): HWe 1.12–1.35 (1.21) HW 1.12–1.35 (1.21) HW1 1.08–1.27 (1.2) HW2 1.21–1.46 (1.32) HW3 0.86–0.90 (0.86) IFW1 0.70–0.88 (0.78) IFW2 0.27–0.33 (0.32) HL1 1.16–1.30 (1.16) HL2 0.96–1.12 (0.96) SL 0.80–0.93 (0.86) EL 0.14–0.19 (0.16) Om 9–11 (11) WL 1.38–1.68 (1.55) PL 0.3–0.44 (0.3) PPL 0.22–0.26 (0.26) GL 0.9–1.18 (0.99) HFL 1.20–1.40 (1.33) PW 0.73–0.94 (0.83) CI 100–104 (104) FLI 63–68 (65) SI 67–73 (71) OI 12–15 (13) CEI 14–17 (17) [N=7]

***Pilosity.*** Pubescence dense, lighter than integument, appressed to decumbent. Hairs curved, darker in color at base, yellow to gray, appressed to suberect, mostly decumbent.

***Head.*** Head in full-face view slightly broader than long (CI=103 ± 2), posterior corner acute, lateral margin of head convex, posterior cephalic emargination distinct, very deep (CEI=15 ± 1), gradually impressed. Vertexal impression relatively deep, frontal tumuli distinct. Mandibles with 7–9 teeth, dorsally smooth, glossy, finely transversely striate along masticatory margin. Frontal carina straight to slightly curved laterally, complete. Eyes medium-sized (OI =14 ± 1), weakly convex, 9–11 ommatidia across largest diameter. Frontal lobe triangular, relatively narrow (FLI=64 ± 2), posterior margin shorter than medial. Antennal relatively short, scape not reaching posterior cephalic corner (SI=71 ± 2).

***Mesosoma.*** Mesosomal tubercles low and obtuse. Propodeal carinae low, reduced, sometimes with posterodorsal denticles.

***Metasoma.*** Petiole and postpetiole each with two low, short, serrate carina dorsally, on petiole sometimes reduced to low denticles, best seen in dorsolateral view. First gastral tergite with lateral carinae weakly developed, dorsal carinae absent.

##### *S. lutzi* queen description.

Measurements in mm: HWe 1.4 HW 1.4 HW1 1.32 HW2 1.52 HW3 IFW1 0.9 IFW2 0.34 HL1 1.36 HL2 1.16 SL 0.92 EL 0.25 Om 18 EW 0.1 WL 2.08 PL 0.44 PPL 0.28 GL 1.72 HFL 1.56 PW 1.18 1 FWg 6.82 HWg 4.6 CI 103 FLI 64 SI 66 OI 18 [N=1]

***Head.*** Mandibles with nine teeth, dorsally glossy and smooth, finely transversely striate only along masticatory margin. Preocular carina fading posterior to eye. Eye large, convex, 18 ommatidia across largest diameter. Frontal lobe as in worker, antennal scape not reaching posterior cephalic corner.

***Mesosoma.*** Scutum in dorsal view with notauli and median mesoscutal line reduced. Parapsidal lines faint, slightly curved. Scutellum in dorsal view narrowing posteriorly, posterior notch shallow. Propodeum with two low, reduced denticles.

***Metasoma.*** First tergite of gaster with lateral carinae strongly developed, dorsal carinae absent, anteromedian groove distinct in dorsal view.

##### *S. lutzi* male.

Measurements in mm: HWe 0.9 HW 0.74 IFW1 0.27 IFW2 017 HL1 0.7 SL 0.74 EL 0.29 Om 30 EW 0.13 WL 1.74 PL 0.4 PPL 0.22 GL 1.4 HFL 1.67 PW 0.80 IOD 0.58 FWg 5.21 HWg 3.55 CI 128 FLI 30 SI 83 OI 32 [N=1]

Head in full-face view longer than broad (CI=128). Eyes large (OI=32), 30 ommatidia across largest diameter. Preocular carina extending posterior to median ocellus, medially curved before fading. In dorsal view scutum with notauli well developed, mesoscutal line distinct, with dark-brown reticulation, best seen in frontodorsal view. Groove between axillae with one transverse, short costa medially. Propodeum devoid of protuberances or carinae except for spiracular tubercles. Petiole with weak lateral carinae; postpetiole simple, smooth, without denticles or carinae.

##### *S. lutzi* geographic range.

Guyana. Map: Figure 31.

##### *S. lutzi* notes.

Based on morphology, *S. lutzi* seems to be most closely related to *S. mayri*, but *mayri* has a much shallower posterior cephalic emargination; striate mandibles; narrower, anterad-directed frontal lobes; and often at least faint anteromedian dorsal gastral carinae.

*S. lutzi* was originally described from a handful of specimens collected on Mt. Roraima, a mountain plateau (tepui) on the border of Guyana, Venezuela, and Brazil. The only other specimens of this species were collected on the slopes of the eastern-most tepui in Guyana, Mt. Ayanganna. It is very likely that this species has a restricted distribution and is endemic to the tepuis of the Guiana Highlands, an area known for its endemic flora and fauna (Brown 1975, Steyermark 1987, Berry et al. 1995, LaPolla et al. 2007).

##### *S. lutzi* material examined.

**GUYANA: Cuyuni-Mazaruni**: Mt. Ayanganna base camp, 5.3344, -59.9248, 732 m, 8 Oct 2002, T. R. Schultz.

#### Sericomyrmex maravalhas sp. n.

Taxon classificationAnimaliaHymenopteraFormicidae

http://zoobank.org/69D8F4AC-C4F2-4293-8092-8D741661C9D7

**Figure 32.**
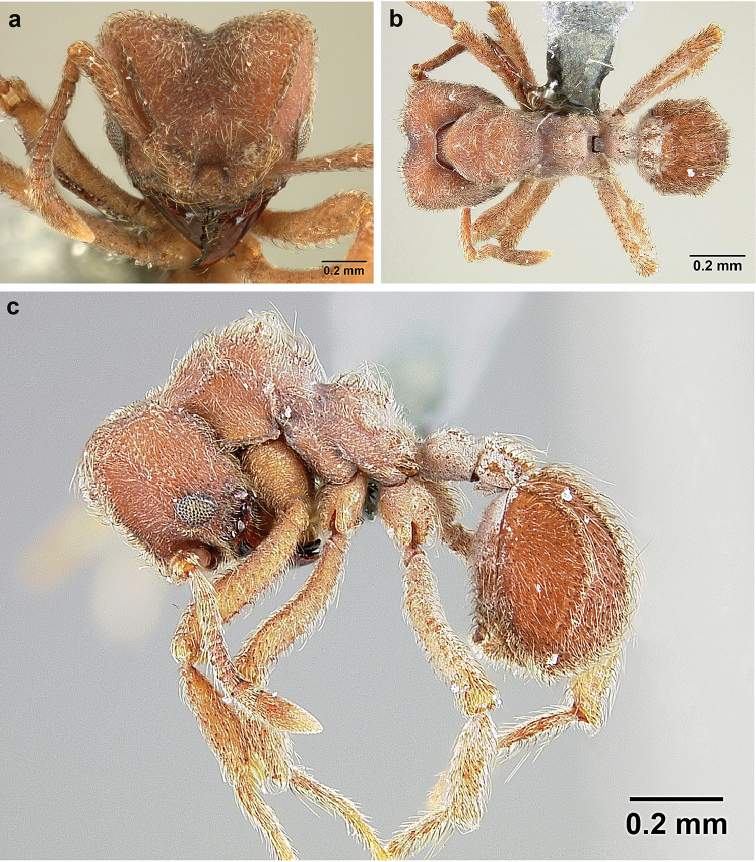
*S. maravalhas* worker (USNMENT00924081). **a** Head **b** dorsal view; and **c** lateral profile.

**Figure 33.**
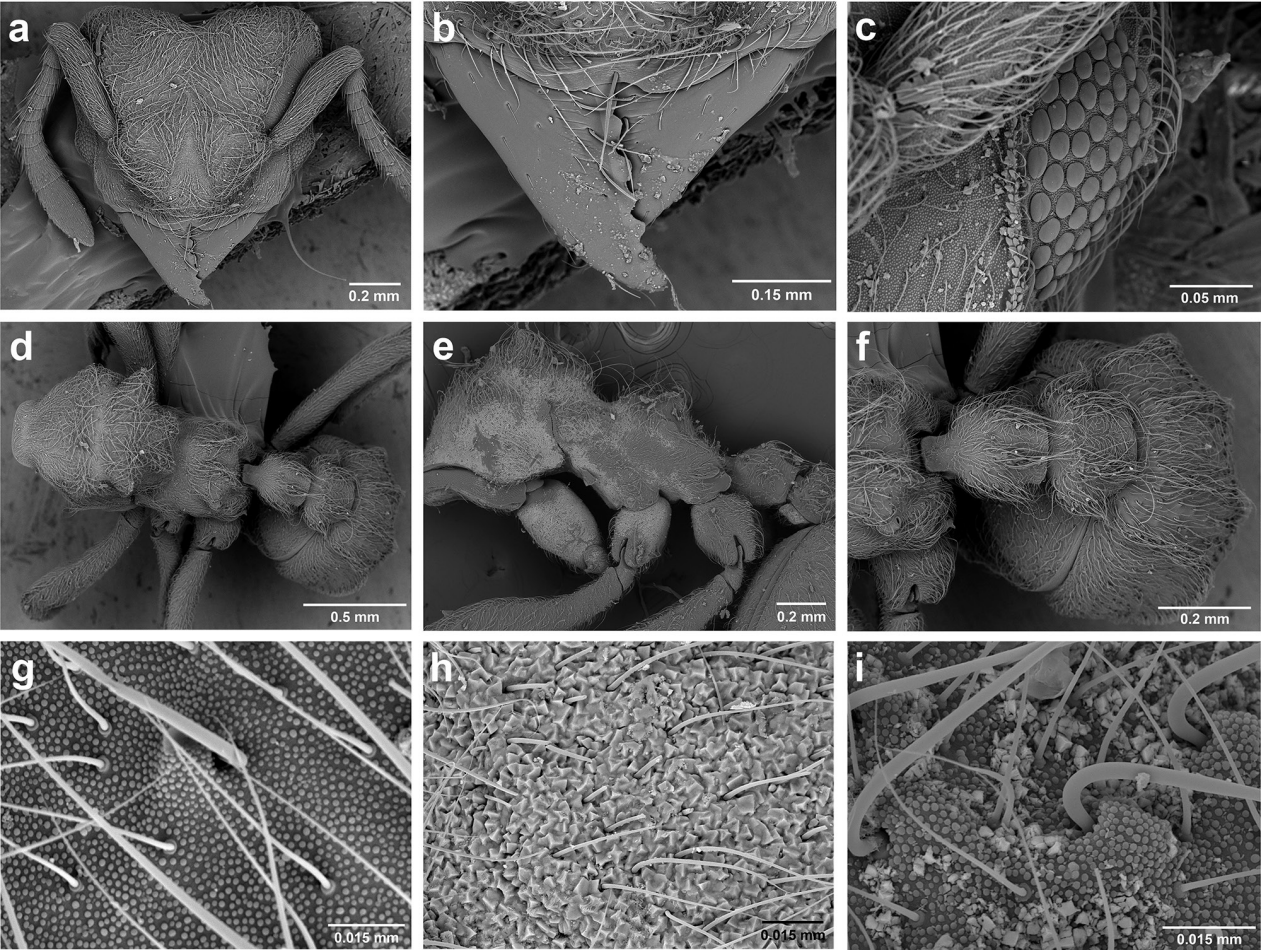
*S. maravalhas* worker (USNMENT00924090: (**a, b, c, d, f, g**) USNMENT01126226 (**e, h, i**), SEM images. **a** Head, full-face view **b** mandibles **c** eye **d** mesosoma and metasoma, dorsal view **e** mesosoma, lateral view **f** metasoma, dorsal view **g** head, papillate integument **h** mesosoma, integument with dense crystal-like layer **i** mesosoma, papillate integument with sparse crystal-like layer.

**Figure 34.**
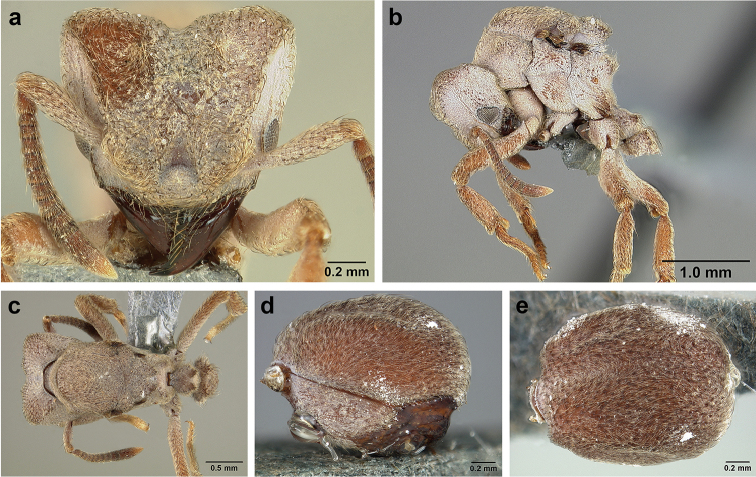
*S. maravalhas* queen (USNMENT00924082). **a** Head **b** lateral profile **c** mesosoma, dorsal view; and gaster **d** lateral and **e** dorsal views.

**Figure 35.**
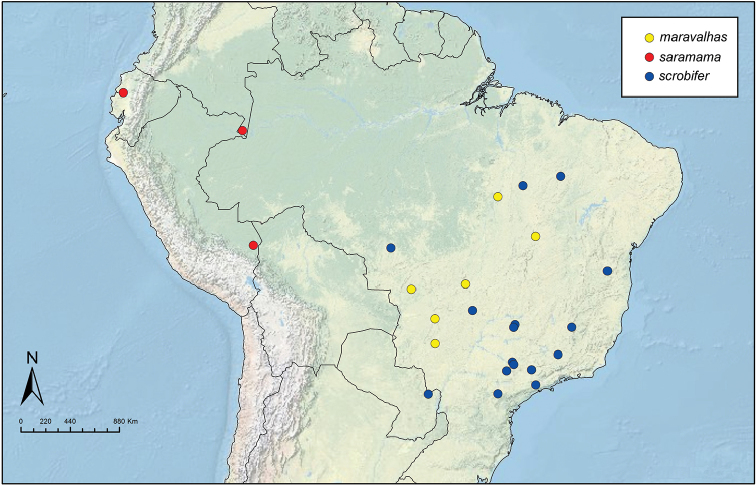
Distribution map of *S. maravalhas*, *S. saramama*, and *S. scrobifer*.

Figures 32 , 33 (Worker); Figure 34 (Queen); Figure 35 (Map)

##### Type material.

*Holotype worker*: BRAZIL, Mato Grosso, Serra Azul State Park, Barra do Garças, -15.8571, -52.2617, 539m, 5 Jun 2011, H. Vasconcelos (MZSP: 1w, USNMENT00924081). *Paratypes*: same data as holotype (MZSP: 1w, USNMENT00924090) (USNM: 1q, USNMENT00924082; 1w, USNMENT00924087; 1w, USNMENT00924089), (MBC-UFU: 1w, USNMENT00924083), (MCZ: 1w, USNMENT00924092), (CASC: 1w, USNMENT00924086; 1w, USNMENT00924091), (BMNH: 1w, USNMENT00924088), (MHNG: 1w, USNMENT00924095).

##### *S. maravalhas* worker diagnosis.

Small species; frontal lobe triangular; frontal carina robust, complete; eye convex, sometimes laterally protruding in full-face view; mandible dorsally glossy, smooth; gaster with four sharp carinae.

##### *S. maravalhas* worker description.

Measurements in mm, range (holotype): HWe 0.80–0.98 (0.95) HW 0.78–1.00 (0.96) HW1 0.75–1.2 (0.98) HW2 0.82–1.08 (1.03) HW3 0.54–0.75 (0.63) IFW1 0.51–0.68 (0.64) IFW2 0.19–0.30 (0.2) HL1 0.77–0.95 (0.88) HL2 0.68–0.83 (0.8) SL 0.58–0.73 (0.68) EL 0.13–0.2 (0.18) Om 8–11 (9) WL 1.03–1.28 (1.2) PL 0.21–0.32 (0.23) PPL 0.13–0.25 (0.21) GL 0.72–0.95 (0.85) HFL 0.82–1.08 (1) PW 0.52–0.71 (0.66) CI 97–110 (108) FLI 57–73 (67) SI 67–77 (72) OI 15–21 (19) CEI 7–17 (8) [N=30]

***Pilosity.*** Pubescence dense, lighter than integument, appressed to decumbent. Hairs curved, darker in color at base, appressed to suberect, mostly decumbent.

***Head.*** In full-face view slightly broader than long (CI=104 ± 3), posterior corner angular to acute, lateral margin of head slightly convex, posterior cephalic emargination distinct (CEI=10 ± 2), gradually impressed. Vertexal impression distinct, frontal tumuli faint. Mandibles with 7–8 teeth, dorsally smooth and shiny, finely transversely striate only along masticatory margin. Eye large (OI =18 ± 1), moderately convex, without white layer, 9–11 ommatidia across largest diameter. Frontal lobe relatively wide (FLI=68 ± 2), triangular, posterior margin shorter than medial, lateral margin in some specimens mildly concave. Frontal carina well developed, complete, reaching posterior cephalic corner. Antennal scape moderately long, sometimes almost reaching posterior cephalic corner (SI=72 ± 3).

***Mesosoma.*** Mesosomal tubercles low and obtuse to moderately pronounced. Propodeal carinae low, sometimes serrate, sometimes with posterodorsal denticles.

***Metasoma.*** Petiole and postpetiole each with two low, short, serrate carinae dorsally, on petiole sometimes reduced to denticles, best seen in dorsolateral view. Postpetiole usually with another pair of low carinae laterally. First gastral tergite with lateral and dorsal carinae strongly developed (Figure 32c, 33f).

##### *S. maravalhas* queen description.

Measurements in mm: HWe 1.12 HW 1.12 HW1 1.24 HW2 1.36 HW3 0.84 IFW1 0.84 IFW2 0.4 HL1 1.12 HL2 0.99 SL 0.75 EL 0.24 Om 20 EW 0.08 WL 1.72 PL 0.33 PPL 0.3 GL 1.6 HFL 1.25 PW 1 CI 100 FLI 75 SI 67 OI 22 [N=1]

***Head.*** Mandible with 8 teeth, dorsally glossy and smooth, finely transversely striate only along masticatory margin. Preocular carina extending posterior to eye, becoming thinner posteriorly, almost converging with frontal carina to form complete scrobe, best seen in lateral view. Eye large, convex, protruding from sides of head in full-face view, 20 ommatidia across largest diameter. Frontal lobe as in worker, antennal scape not reaching posterior cephalic corner (SI=67).

***Mesosoma.*** Lateral pronotal tubercles low and obtuse. Scutum in dorsal view with notauli and median mesoscutal line faint. Parapsidal lines thin, slightly curved. Groove separating axillae in dorsal view weakly transversely costate. Scutellum inflated, short in dorsal view, narrowing posteriorly, with relatively deep V-shaped posterior notch. Propodeum with two obtuse, laterally flattened, diverging denticles.

***Metasoma.*** First gastral tergite with lateral carinae strongly developed, dorsal carinae faint, anteromedian groove distinct.

##### *S. maravalhas* male.

Unknown.

##### *S. maravalhas* geographic range.

Interior of Brazil, cerrado habitats. Map: Figure 35.

##### *S. maravalhas* notes.

*S. maravalhas* can be separated from its sister species *S. scrobifer* by its smaller size; narrower, triangular frontal lobes (trapeziform in *scrobifer*); less robust frontal carinae; and eyes somewhat smaller and flatter (Figure 5d). The closely related *S. saramama* is similar in size but differs from *maravalhas* by the absence of dorsal gastral carinae, less robust frontal lobes and frontal carinae, and smaller, flatter eyes. *S. opacus*, which is similar in size and has smooth mandibles, can be distinguished from *maravalhas* by the absence of dorsal gastral carinae; smaller, flat eyes, sometimes with a white layer; weaker, incomplete frontal carinae; and *opacus*-typical rectangular frontal lobes.

In addition to characters that are the same as in the worker, the most diagnostic character of the *S. maravalhas* queen is the presence of fully developed preocular carinae that almost join with the frontal carinae posteriorly, a relatively deep notch in the posterior margin of the scutellum, and large propodeal denticles. However, because our description is based on a single specimen, we do not know if these characters vary within the species.

Sister species *S. maravalhas* and *S. scrobifer* have consistent morphological and molecular differences and their distributions overlap, possibly substantially, which reinforces our decision to recognize them as distinct species. However, distributional data for both species are clearly incomplete (Figure 35) and it remains entirely possible that, due to undersampling, we may be unaware of forms that are morphologically and/or molecularly intermediate between the two species, which, if they exist, might compel us to reevaluate them to be a single species. Currently, however, based on all of the material examined, the results of the morphological measurement analyses (Figure 3FLI, Figure 5d), and the branch lengths separating the two species in the molecular phylogeny (Suppl. material 1), we have chosen to recognize *maravalhas* as distinct from *scrobifer*.

An interesting feature of *S. maravalhas* is that, unlike adult workers of all other *Sericomyrmex* species that we examined under SEM, some of the *maravalhas* workers lack the thick, waxy, crystal-like cuticular layer (Figure 33h). In *S. maravalhas* this layer can be entirely (Figure 33g) or partially (Figure 33e, i) absent so that the minutely papillate integument is visible (Figure 33g), a condition otherwise known only in males and callow workers of other *Sericomyrmex* species (Figure 6d–e). We know nothing about the chemical composition or function of this white, crystal-like layer, but the two most likely explanations are that it is either a cuticular secretion or microbial in origin. Considering the known complex microbial interactions in the attine ant symbiosis (Currie et al. 2003, 2006, Fernández-Marín et al. 2006, Little and Currie 2007), and considering that this layer covers the eyes of some *Sericomyrmex* species (see *S. saussurei* notes for discussion), this phenomenon needs to be further investigated.

##### *S. maravalhas* etymology.

This species is named after our myrmecologist colleague Jonas Maravalhas, who sorted and sent to us the specimens of this species used in our molecular phylogenetic analyses. The molecular data were crucial, in combination with morphological evidence, for recognizing *maravalhas* as a distinct species. Even better, Jonas’ surname has the same root as “*maravilhas*” which means “wonders” in Portuguese, an appropriate adjective for this new species. The species name is a noun in apposition.

##### Material examined.

**BRAZIL: Mato Grosso**: Coxim, Rio Taquari, [-18.5264, -54.7465], 1 Dec 1963, V. C. Andzada; Near Poconé, Transpantaneira Km115, [-16.2597, -56.6269], 28 Nov 1984, J. C. Trager; Serra Azul State Park, Barra do Garças, -15.8571, -52.2617, 539m, 5 Jun 2011, H. Vasconcelos; **Mato Grosso do Sul**: Campo Grande, -20.4261, -54.7275, 532m, 7 Oct 2012; **Tocantins**: Araguacema, Rio Tiririca, -8.9886, -49.6675, 16 Nov 2005, R. R. Silva, R. M. Feitosa; Ponte Alta do Bom Jesus, -12.1212, -46.6176, 7 Oct 2004, R. R. Silva, B. H. Dietz.

#### Sericomyrmex mayri

Taxon classificationAnimaliaHymenopteraFormicidae

Forel, 1912

**Figure 36.**
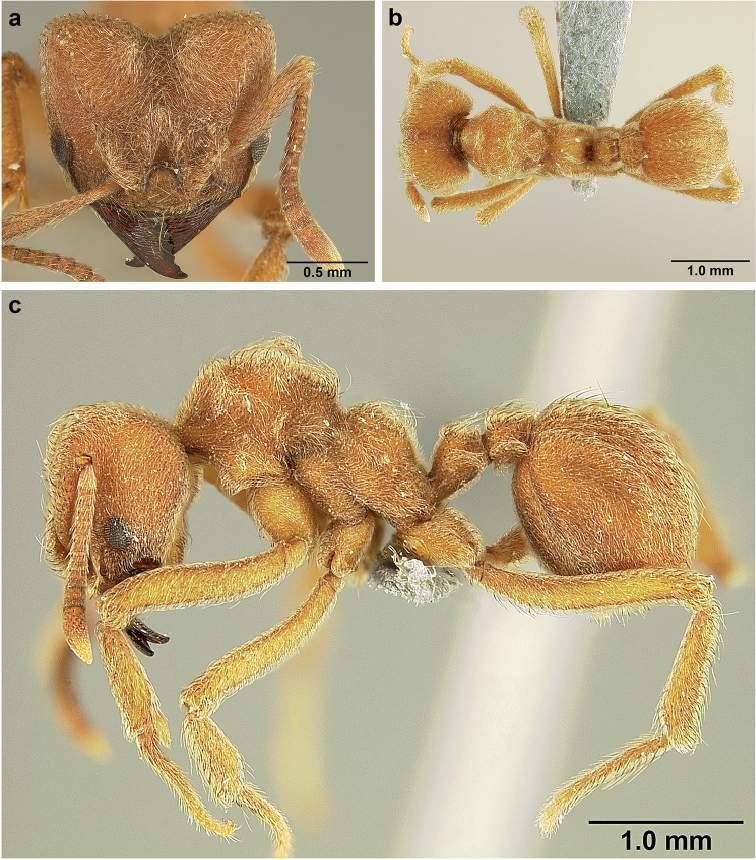
*S. mayri* worker (USNMENT01125171). **a** Head **b** dorsal view; and **c** lateral profile.

**Figure 37.**
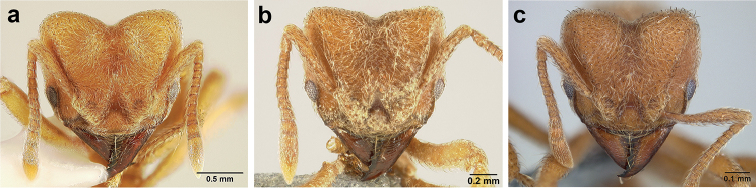
*S. mayri* worker variation; head, full-face view. **a** *mayri* worker from Brazil, Amazonas (USNMENT00444066) **b** *mayri* worker from Brazil, Bahia (USNMENT01125172) **c** *mayri* worker from Colombia, Meta (USNMENT01125151).

**Figure 38.**
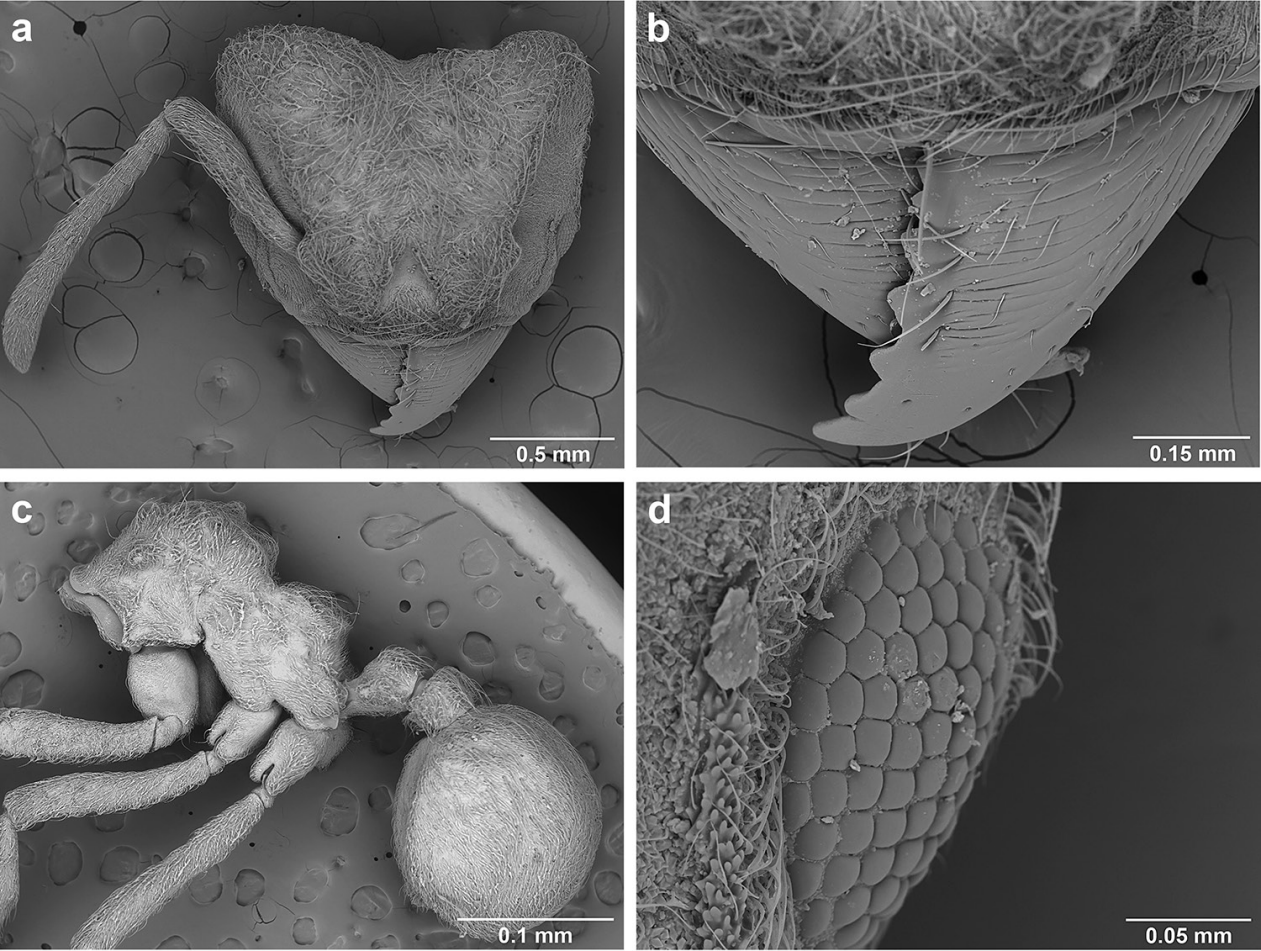
*S. mayri* worker (USNMENT01126229), SEM images. **a** Head, full-face view **b** mandibles **c** mesosoma and metasoma, lateral view **d** eye.

**Figure 39.**
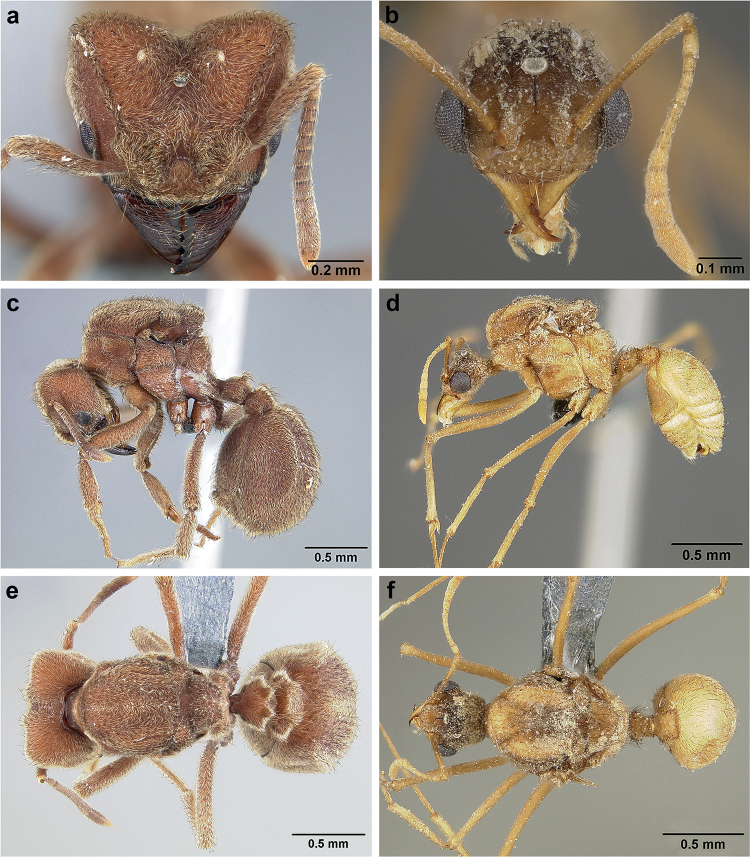
*S. mayri* queen and male; head, lateral profile, and dorsal view. Queen (USNMENT01126007) (**a, c, e**). Male (USNMENT01126022) (**b, d, f**).

**Figure 40.**
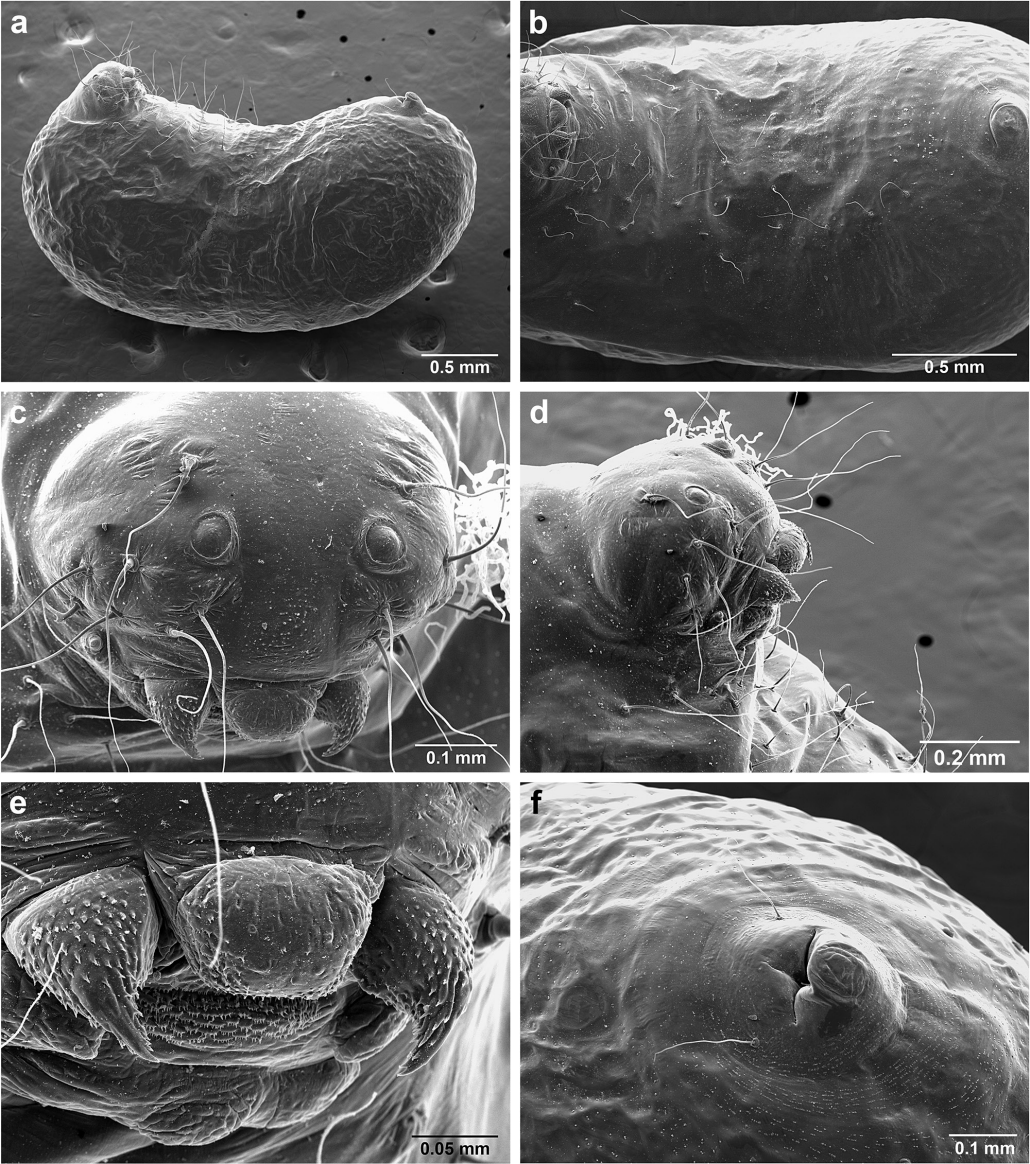
*S. mayri* larva (USNMENT01126227: **a, f** USNMENT01126230: **b, c, d, e**), SEM images. **a** Lateral view **b** ventral view **c** head, frontodorsal view **d** head, lateral view; **e** mouthparts **f** anal setae.

**Figure 41.**
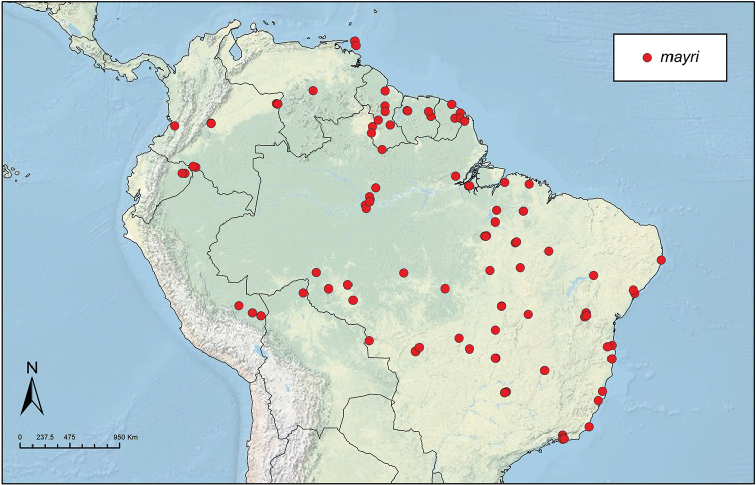
Distribution map of *S. mayri*.

Figures 36 , 37 , 38 (Worker); Figure 39 (Queen and male); Figure 40 (Larva); Figure 41 (Map)

Sericomyrmex mayri Forel, 1912: 194. Lectotype worker (here designated): BRAZIL, Rio de Janeiro, Niterói, [-22.8751, -43.2775], ANTC31816, A. Forel, (MHNG: 1w, CASENT0909370). *Paralectotypes*: same data as lectotype (MHNG: 1w, USNMENT00445567; 3m, USNMENT00445580).
Sericomyrmex urichi = Forel, 1912: 193. **syn. n.** Type material examined: TRINIDAD AND TOBAGO, ANTC31818, F. W. Urich (MHNG: 3w, CASENT0909372).
Sericomyrmex luederwaldti = Santschi, 1925: 15. **syn. n.** Type material examined: BRAZIL, Minas Gerais, Pirapora, [-17.355, -44.9447], ANTC35978, ANTC25817, E. Garbe (NHMB: 5w, CASENT0912516) (MSNG: 1w, CASENT0904989).
Sericomyrmex moreirai = Santschi, 1925: 16. **syn. n.** Type material examined: BRAZIL, Rio de Janeiro, [-22.8751, -43.2775], ANTC35979, Moreira (MCZ: 2w, MCZ 1-2 21140) (NHMB: 3w, CASENT0912517; 2w, USNMENT01126231; 2q, USNMENT01126232).
Sericomyrmex harekulli = Weber, 1937: 398. **syn. n.** Type material examined: GUYANA, East Berbice-Corentyne, Oronoque River, [2.75, -57.4167], NAW598, 27 Jul 1936, N. A. Weber (USNM: 1w, USNMENT00529483) (MCZ: 2w, USNMENT00924104; 2w, USNMENT00924105)
Sericomyrmex harekulli arawakensis = Weber, 1937: 399. **syn. n.** Type material examined: GUYANA, Cuyuni-Mazaruni, Mazaruni River, Forest Settlement, [6.39733, -58.6781], 10 m, NAW 277, 15 Aug 1935, N. A. Weber (MCZ: 2w, MCZ 23051; 2w, 1q, USNMENT00924106)

##### *S. mayri* worker diagnosis.

Large species; head broad; frontal lobe narrow, directed anterad; mandible usually striate; frontal carina often reduced, incomplete; eye flat to mildly convex; posterior cephalic margin shallow, abruptly to gradually impressed; posterior cephalic corner usually angled; mesosomal tubercles low and obtuse, first gastral tergite with lateral carina well developed, dorsal carinae absent or faint.

##### *S. mayri* worker description.

Measurements in mm, range (lectotype): HWe 1.05–1.60 (1.4) HW 1.05–1.64 (1.43) HW1 1.02–1.68 (1.44) HW2 1.12–1.8 (1.6) HW3 0.74–1.12 (1.05) IFW1 0.66–1.00 (0.93) IFW2 0.24–0.40 (0.35) HL1 1.02–1.52 (1.33) HL2 0.93–1.36 (1.21) SL 0.74–1.08 (0.99) EL 0.15–0.35 Om 10–13 WL 1.27–2.2 (1.84) PL 0.24–0.47 (0.4) PPL 0.18–0.35 (0.32) GL 0.92–1.42 (1.24) HFL 1.15–1.7 (1.58) PW 0.68–1.2 (1.05) CI 101–115 (105) FLI 59–68 (66) SI 61–76 (71) OI 11–26 CEI 5–19 (9) [N=103]

***Pilosity.*** Pubescence dense, often lighter colored than integument, appressed to decumbent. Hairs straight to curved, darker in color at base, yellow to gray, appressed to suberect, mostly decumbent.

***Head.*** In full-face view broader than long (CI=108 ± 3), posterior corner angular to acute, lateral margin of head straight, sometimes slightly convex. Posterior cephalic emargination distinct, shallow, usually abruptly, sometimes gradually impressed, variable within species and within colonies. Vertexal impression relatively deep, pronounced, sometimes extending anterad to include frons (Figure 36a), frontal tumuli often distinct. Mandibles with 7–8 teeth, dorsally glossy, usually striate. Frontal carina straight to slightly curved laterally, usually fading before reaching posterior cephalic corner, sometimes complete. Eye small to medium-sized (OI=14 ± 1), flat to slightly convex, lacking white layer, 10–13 ommatidia across largest diameter. Frontal lobe triangular, narrow (FLI=63 ± 2), posterior margin much shorter than medial, lateral margin long, directed anterad. Antennal scape short (SI=68 ± 3), never reaching posterior cephalic corner.

***Mesosoma.*** Mesosomal tubercles low and obtuse. Propodeal carinae low, without posterodorsal denticles, or denticles low and weak.

***Metasoma.*** Petiole and postpetiole each with two low, short, serrate, longitudinal carinae dorsally, on petiole sometimes reduced to low denticles, best seen in dorsolateral view. Postpetiole usually with another pair of low carinae laterally. First gastral tergite with lateral carinae well developed, dorsal carinae absent or faint.

##### *S. mayri* queen description.

Measurements in mm, range: HWe 1.44–1.64 HW 1.48–1.68 HW1 0.52–1.76 HW2 1.64–1.84 HW3 1.03–1.2 IFW1 0.98–1.13 IFW2 0.36–0.45 HL1 1.4–1.56 HL2 1.24–1.4 SL 0.96–1.09 EL 0.24–0.3 Om 16–21 EW 0.08–0.13 WL 2.12–2.5 PL 0.45–0.65 PPL 0.25–0.4 GL 1.76–2.21 HFL 1.5–1.85 PW 1.24–1.46 FWg 6.56–8.03 HWg 4.29–5.28 CI 100–109 FLI 64–72 SI 62–70 OI 16–19 [N=15]

***Head.*** Mandible with 8–9 teeth, dorsally striate. Preocular carina fading posterior to eye, rarely (in one queen from Ecuador) 1–3 supraocular carinae also present, not reaching posterior cephalic corner. Eye large (OI=18 ± 1), convex, sometimes mildly notched posteriorly, 16–21 ommatidia across largest diameter. Frontal lobe as in worker, antennal scape not reaching posterior cephalic corner.

***Mesosoma.*** Lateral pronotal tubercles low and obtuse. Scutum in dorsal view with notauli faint, median mesoscutal line sometimes anteriorly developed into weak costa, posteriorly with shallow longitudinal impressions on each side. Parapsidal lines thin, slightly curved. In dorsal view scutellum short, narrowing posteriorly, posterior notch shallow, sometimes continuing into median impression that divides scutellum in two lateral parts. Propodeal denticles reduced, low.

***Metasoma.*** Petiole in frontodorsal view with two pointed, distinct dorsal denticles, and two smaller, lateral denticles. Postpetiole with two dorsal and two lateral short, low carinae, sometimes reduced to small denticles. First gastral tergite with lateral carinae strongly developed, dorsal carinae weak, anteromedian groove distinct.

##### *S. mayri* male description.

Measurements in mm, range: HWe 0.84–1.02 HW 0.71–0.84 IFW1 0.32–0.38 IFW2 0.15–0.27 HL1 0.72–0.8 SL 0.77–0.85 EL 0.31–0.36 Om 24–32 EW 0.13–0.16 WL 1.88–2.05 PL 0.35–0.56 PPL 0.24–0.33 GL 1.32–1.8 HFL 1.9–2.2 PW 0.87–1.12 IOD 0.65–0.74 FWg 5.4–6.25 HWg 3.79–4.29 CI 105–131 FLI 34–42 SI 78–96 OI 31–42 (N=8)

Head in full-face view longer than broad (CI=124 ± 8). Eye large (OI=36 ± 3), convex, 24–32 ommatidia across largest diameter. Preocular carina long, extended posteriorly beyond lateral ocellus, slightly curved medially before fading. Notauli and mesoscutal line well developed, surrounding integument usually lighter colored, often reticulate, groove between axillae with 1–4 transverse keels. Propodeum smooth, without any protuberances except spiracular tubercles. Petiole with two lateral and two dorsal low, serrate carinae, postpetiole with reduced lateral denticles.

##### *S. mayri* larva description.

Two to four setae on each side of lateral body surfaces, none dorsally. Supra-antennal setae present. Six genal setae on each side. Mandibular apical tooth divided. Labial denticles absent. First thoracic segment ventrally with multiple multidentate spinules, arranged in transverse rows. Numbers of ventral hairs: ten to fourteen on T1, six on T2, four to six on T3, two to eight on abdomen (not including anal setae). Single pair of setae anterior to anal opening, no additional setae laterally.

##### *S. mayri* geographic range.

Bolivia, Brazil, Colombia, Ecuador, French Guiana, Guyana, Peru, Suriname, Trinidad and Tobago, Venezuela. Map: Figure 41.

##### *S. mayri* notes.

*S. mayri* can be separated from both *S. amabilis* and *S. saussurei* by its large size, broad head, narrow frontal lobes, and gaster lacking anteromedian dorsal carinae. In addition, *saussurei* has eyes covered with a thick white layer, which is never the case in *mayri. Sericomyrmex lutzi* is similar in size, but *lutzi* has a characteristic, much deeper cephalic emargination and smooth mandibles. In addition to worker characters, the *S. mayri* queen can be separated from the sympatric *S. saussurei* queen by its more pronounced petiolar denticles.

Variation within *S. mayri* includes the scape length, the head shape, and mandibular striation (Figure 37). As with *S. amabilis* and *saussurei*, two other species with striate mandibles, some individuals or populations of *S. mayri* have smooth or faintly striate mandibles. These alternative states are encountered less commonly in *mayri* than in the other two species, mostly in populations from Trinidad and Tobago (Figure 37a). Intermediate-state workers (i.e., those with faint mandibular striation) were collected at the same locality and, indeed, were found within the same nest as workers with typically striate mandibles. A callow worker of *mayri* studied with SEM had smoother, faintly striate mandibles (Figure 6i), so some observations of smooth mandibles may be due to sampling of recently eclosed workers, but this is unlikely to explain smooth-mandibled foragers.

The presence/absence of the anteromedian dorsal gastral carinae and robustness of the lateral gastral carinae are also variable in *mayri*. Typically, both carinae are present, but in some specimens the anteromedian carinae are very faint or absent, while lateral carinae can be weak to robust. The posterior cephalic emargination can be very shallow, so that the posterior margin of the head appears almost straight (e.g., specimens from Minas Gerais, Uberlandia, Brazil, and the type series of *S. moreirai*). The shape of the head varies to some extent, from distinctly broad in some specimens to more narrow in others (the CI range is wide: 101–115).

**Figure 42.**
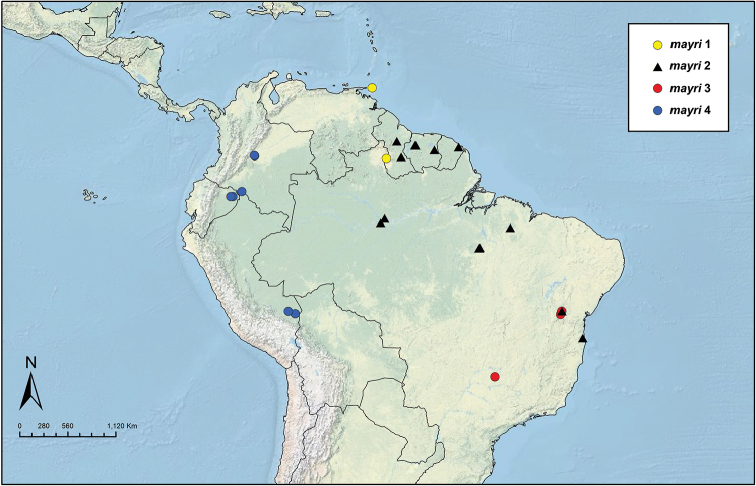
Distribution map of *S. mayri* populations. This map is based on the subset of *S. mayri* samples for which molecular data (either UCE or COI) were obtained. Detailed specimen locality data are included in Suppl. material 2: Table S2g.

We studied variation in the morphology of *S. mayri* with reference to the molecular phylogeny (Suppl. material 1), in which there is high statistical support for four subspecific clades within *S. mayri*, in order to investigate whether any of these separate, well-supported subclades could represent separate species. We found no consistent morphological differences between those four molecular clades (Figure 5c). Geographical separation can explain those clades to some extent (Figure 42), although the distribution ranges of two of the populations overlap in Brazil. Further, one specimen collected in Lençios, Brazil, has a COI sequence that differs from those of the other samples collected in that same locality, instead grouping with the other Brazilian *S. mayri* population. This could indicate the presence of two genetically distinct, sympatric populations, which would be consistent with a hypothesis of two separate Brazilian species or of introgression between two incipient species. However, we do not have UCE data for this sample and a previous comparative study of UCE and COI data for *Sericomyrmex* (Ješovnik et al., 2017) indicates that COI is inadequate for delimiting species, especially in the *mayri* and *bondari* clades. Further, these two populations are morphologically indistinguishable (Figure 5c). Therefore, based on our morphological studies and inconclusive molecular evidence, we currently regard *S. mayri* as a single, widespread species with molecularly and geographically distinct populations. We find *S. mayri* interesting in terms of population structure, and we report these inconclusive data to encourage further research. Population study of *S. mayri* with finer geographic sampling could be important for understanding speciation and population genetics in attine ants.

*Synonymy.* The examined syntypes of *S. luederwaldti*, *S. harekulli*, and *S. harekulli arawakensis* conform to typical *S. mayri* morphology. Their original authors (Santschi 1925, Weber 1937) focus on slight differences in mesosomal tubercles, head shape, and scape length, all of which are variable within *mayri*. The *moreirai* syntypes have the cephalic emargination less pronounced than in the *mayri* lectotype, but this difference is encompassed by the range of variation in *mayri* as here defined. In his description of *S. moreirai*, Santschi (1925) calls it the “neighbor” of *mayri*, but says it is “much more stocky.” He also compares *moreirai* with *urichi* and reports small differences in pilosity and mesosomal tubercles, both of which fall within the variation observed in *S. mayri*. The syntypes of *urichi* we examined, unlike the *mayri* lectotype, have almost completely smooth mandibles, but, as discussed above, smooth mandibles are encountered in some *mayri* populations, especially those from Trinidad and Tobago, the type locality of *urichi*. In all other characters and measurements, *urichi* clearly agrees with *S. mayri*. In his description Forel (1912) distinguished *mayri* and *urichi* by complete versus incomplete frontal carinae and by the depth of the cephalic emargination, but he does not mention striate vs. smooth mandibles. Again, the cited differences (depth of the emargination, length of the frontal carinae, and degree of mandibular sculpture) fall within the range of observed intraspecific variation in *S. mayri* as here defined.

##### Material examined.

**BOLIVIA: Beni**: Vaca Diez, nr. Reserva Ecológica El Tigre, [-10.8667, -65.75], 172m, 1 Jul 1999, R. Dunn; **BRAZIL: Amapá**: Oiapoque, [3.8333, -51.8333], 1 May 1979, W. L. Overal; **Amazonas**: Manaus, Rs2303, [-3.1133, -60.0253], 30 Sep 1993, A. B. Casimiro; Manaquiri, Br 319, km100, [-3.4, -60.4], 10 Oct 2010, F. Baccaro; Manaus, Reserva Ducke, [-2.917, -59.983], 9 Aug 1992, T. R. Schultz; Pres. Figueredo, I. Pe Inchado, -1.8971, -59.4865, 23 Aug 1993, Queiroz; Reserva Campina, EEST km 44, [-2.67, -60.03], 18 Aug 1992, T. R. Schultz; **Bahia**: Andaraí, Mata Carrasco (castanha), [-12.8055, -41.3312], 13 Dec 1990, C. R. F Brandão, Diniz, Oliveira; NP Chapada Diamantina - Mucugê, -12.9053, -41.5005, 1032m, 6 Sep 2009, E. Borges; Lençóis, nr. NP Chapada Diamantina, -12.5598, -41.3708 ±5m, 530m, 9 Nov 2008, J. Sosa-Calvo; Una, Fazenda Ararauna, -15.3071, -39.1626, 80m, 9 May 2014, I. O. Fernandes; **Espírito Santo**: Aracruz, [-19.8156, -40.3244], 10 Dec 1980, E. Campinos, D. R. Smith; Parque Sooretama, Linhares, -19.0725, -39.9491, 31 Mar 2004, J. H. C. Delabie; **Goiás**: Campo Limpo, Faz. Conceição, -16.3308, -49.1636, 20 Jan 2005, R. R. Silva, R. M. Feitosa; Colinas do Sul, Serra da Mesa, -14.0166, -49.2, 2 Dec 1995, B. H. Dietz, Campaner; Fazenda Pau Brasil, Reserva 19, -15.5813, -51.3987, 310m, 8 Apr 2008, S. E. Solomon; Ouro Verde, Faz Boa Vista, -16.3308, -49.2118, 1 Jul 2005, R. R. Silva, R. M. Feitosa; **Maranhão**: Bom Jardim, REBIO Gurupi Parcela 01 08, -3.9258, -46.7712, 20 Sep 2014, A. Y. Harada; Estreito, Fazenda Itaueiras, -6.5317, -47.3711, 1 Jun 2005, R. R. Silva, R. M. Feitosa; **Mato Grosso**: Chapada dos Guimarães, Cachoeira Pedra Furada, [-15.4671, -55.7363], 15 Feb 1985, J. C. Trager; Hwy to Santo Antônio do Leverger, 10 km S Cuiabá, [-15.780, -56.0638], 16 Feb 1985, J. C. Trager; Mata São João, Reserva Sapiranga, -12.5581, -33.0431, 21 Jun 2001, R. R. Silva, R. M. Feitosa, C. R. F Brandão; Xingu, [-10.5233, -53.5264], 1 Nov 1961, Alvarenga & Werner; **Minas Gerais**: Cachoeira da Fumaça (district of Novo São Joaquim), -14.697, -52.312, 288m, 5 Nov 2011; Panga, Uberlândia, -19.1831, -48.4014, 813m, 19 Oct 2012, A. Ješovnik; Serra de Ricardo Franco State Park, -14.9076, -60.0646, 200m, 11 Feb 2014, J. Maravalhas; Unaí, Fazenda Santo Antônio, -16.7544, -46.4825, 1 Feb 2014, L. N. Paolucci; **Pará**: Baixo Amazonas, [-1.4256, -48.3906], 1 Feb 1949, C. R. Gonçalves; Goianésia, Faz. Rio Capim, [-3.8384, -49.0986], 1 Jun 2003, A. M. Elizabeth; Melgaço, Caxiuanã, -1.7248, -51.4230, 10 Oct 2006, A. Y. Harada; Nova Ipixuna, Fazenda Bom Retiro, -4.8412, -49.218, 12 Jun 2012, M. Tavares, A. Palmeira; Parauapebas, FL Nacional de Carajás, Parque Zoobotânico, -6.0629, -50.0571, 626m, 2 Oct 2014, A. Ješovnik; Viseu São Jose do Gurupi, Parcela 3, -1.5718, -46.2672, 10 Aug 2014, A. Y. Harada; **Pernambuco**: Recife, [-8.096, -34.904], 1 Jan 1988, L. Lima Castro; Tapera, [-9.4272, -40.7218], B. Pickal; **Piaui**: Rio Uruçuí-Preto, [-7.3431, -44.6168], 20 Feb 1976, R. Negrett; **Rio de Janeiro**: Belford Roxo, [-22.7631, -43.3991], 15 Jun 1936, C. R. Gonçalves; Rio de Janeiro DF, [-22.8751, -43.2775], 1 Mar 1940, C. R. Gonçalves; Nova Iguaçu, ReBio Tinguá, -22.5705, -43.4141, 2 Feb 2002, A. Mayhe, S. Veiga-Ferreira; Represa Rio Grande Guanabara, [-22.9167, -43.4167], F. M. Oliveira; São Bento, [-21.9167, -41.1167], 25 Apr 1946, A. Silva; **Rondônia**: Fazenda São Sebastião, [-10.5336, -63.5457], 7 Oct 2008, S. E. Solomon; Ilha Pedras, km1, subparcela 150, -9.1744, -64.61222, 86m, 25 Oct 2013, I. O. Fernandes; Ouro Preto do Oeste, Res do INPA No 0078, [-10.2, -61.9], 26 Mar 1985, F. F. Ramos; **Tocantins**: Aguiarnópolis, -6.6137, -47.4814, 1 Jun 2005, R. R. Silva, R. M. Feitosa; Araguacema, -8.9888, -49.6780, 16 Nov 2005, R. R. Silva, R. M. Feitosa; Aurora do Tocantins, -12.6985, -46.3604, 9 Oct 2004, R. R. Silva, R. M. Feitosa; Gurupi, -12.0111, -48.6783, 30 Sep 2001, R.R. Silva, N.L. Albuquerque; Peixe, Fazenda Galileia, Transecto 1, -11.9788, -48.6591, 30 Sep 2001, R.R. Silva, N.L. Albuquerque; Recursolândia, Mata Ciliar Rio Mateiros, -8.7579, -47.0388, 9 May 2005, R. R. Silva, B. H. Dietz; **COLOMBIA: Meta**: San Martín, El Caduceo Reserve, 3.6694, -73.6585, 374m, 30 Sep 2007, T. R. Schultz; San Martín, El Caduceo Reserve, 3.6292, -73.6256, 364m, 1 Oct 2007, J. Sosa-Calvo; **Putumayo**: PNN La Paya Cabaña La Paya, -0.0333, -75.2, 330m, 15 Dec 2001, E. Lozano; PNN La Paya Cabaña Vivano Cocha, -0.1166, -74.9666, 320m, 30 Nov 2001, R. Cobete; **Valle del Cauca**: PNN Farallones de Cali, Anchicayá, [3.4333, -76.8], 730m, 20 Jul 2000, S. Sarria; **Vichada**: Cumaribo, Cgto. Santa Rita, PNN El Tuparro, 5.3555, -68.0244, 135m, 1 Feb 2004, I. Quintero, E. Gonzales; Cumaribo, Cgto. Santa Rita, PNN El Tuparro, 5.3316, -67.8908, 135m; **ECUADOR: Napo**: Tiputini, La Selva, Chorongo trail, -0.6382, -76.1493, 16 Jun 2003, A. Little; **Orellana**: Estación Chiruisla-Petrobras, Río Huiririma, -0.6438, -75.9124, 18m, 10 Sep 2005, D. Donoso; **FRENCH GUIANA**: **Cayenne**: Kaw Mt., Amazon Nature Lodge, 4.55, -52.2, 950m, 20 Jul 2005, T. R. Schultz; Nouragues Field Station, XII trail, 4.09, -52.6768, 75m, 27 Jul 2005, T. R. Schultz; Paracou Experimental forest, 5.2808, -52.9465, 10 Jul 1999, S. Durou; **Régina**: Nouragues Field Station, XII trail, 4.09, -52.6768, 75m, 27 Jul 2005, T. R. Schultz; **GUYANA: Cuyuni-Mazaruni**: Mabura Hill, camp at the end of Rd. to Lethem, 5.1552, -58.6997, 64m, 30 Oct 2002, J. S. La Polla; Mazaruni River, Forest Settlement, 6.3973, -58.6781, 1 Aug 1935, N. A. Weber; **Potaro-Siparuni**: Iwokrama, Kurapakari base Camp, [4.6698, -58.6854], 60m, 7 Apr 1996, T. R. Schultz, U. G. Mueller; **Upper Takutu-Upper Essequibo**: Annai-Georgetown Rd., nr. Essequibo Riv., [3.93, -59.27], 9 Apr 1996, T. R. Schultz, U. G. Mueller; Karanambo, [3.36, -59.78], 5 Apr 1996, T. R. Schultz, U. G. Mueller; Kusad Mountains, 2.8120, -59.8668, 135m, 26 Oct 2013, J. A. Helms; Upper Essequibo, CI concession, BBC camp, 3.5059, -58.2334, 11m, 21 Nov 2011, A. Ješovnik; Acarai Mountains, nr. Romeo’s camp, 1.3833, -58.9333, 282m, 7 Oct 2010, T. R. Schultz; **PERU: Madre de Dios**: Pakitza, PN Manu, [-11.95, -71.2833], 13 Feb 1992, R. Cambra, D. Quintero; Puerto Maldonado, Los Amigos Biol. Station, -12.569, -70.1005, 272m, 23 Nov 2005, J. Sosa-Calvo; Tambopata Reserve, -12.8187, -69.3636, 224m, 1 Aug 2012, A. Ješovnik; **SURINAME: Brokopondo**: Maripaheuvel, near Dam on Sara creek, [4.67, -54.95], 1 Sep 1959, I. v. d. Drift; Poeroe man Kemisa, [4.67, -54.95], 1 Sep 1959, I. v. d. Drift; **Sipaliwini**: Bakhuis Mountains, 4.7208, -56.726, 249m, 5 Mar 2006, J. Sosa-Calvo; Lely Mts., 4.2529, -54.7561, 619m, 26 Oct 2005, J. Sosa-Calvo; Nassau Mountains, 4.2529, -54.7561, 619m, 26 Oct 2005, J. Sosa-Calvo; **TRINIDAD AND TOBAGO: Tunapuna-Piarco**: Simla Research Station, [10.6836, -61.2833], 240m, 8 Jan 1995, U. G. Mueller; **VENEZUELA: Bolívar**: Río Tawadu, Nichare Field Stn., 6.4333, -64.8833, 200m, 9 Feb 1966, D. M. Olson.

#### Sericomyrmex opacus

Taxon classificationAnimaliaHymenopteraFormicidae

Mayr, 1865

**Figure 43.**
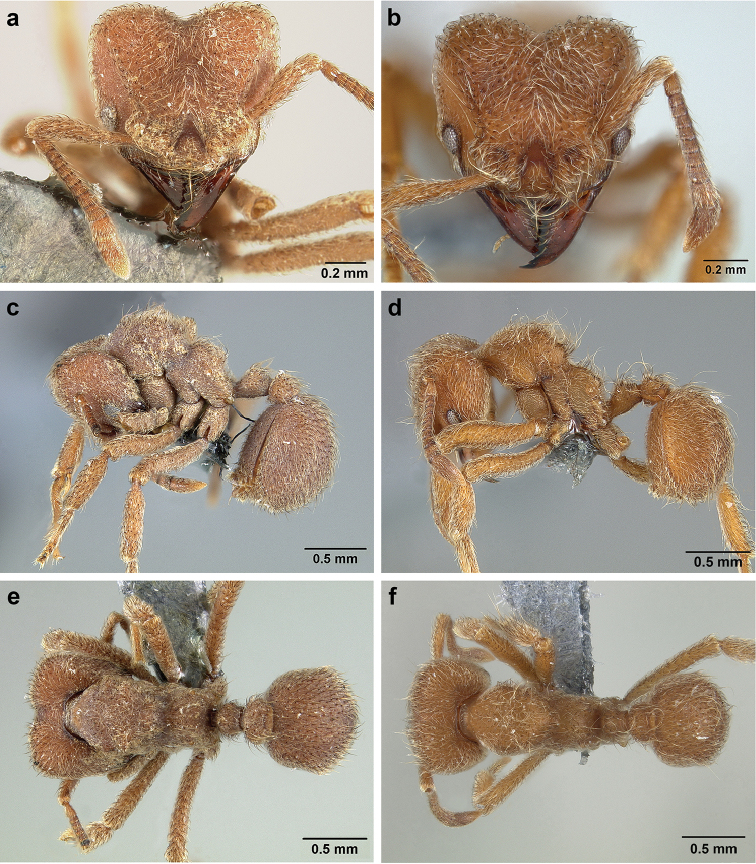
*S. opacus* workers; head, lateral profile, and dorsal view. Comparison of two conspecific individuals, one with the white layer over the eyes (USNMENT01125124) (**a, c, e**); and one without the white layer (USNMENT01125118) (**b, d, f**).

**Figure 44.**
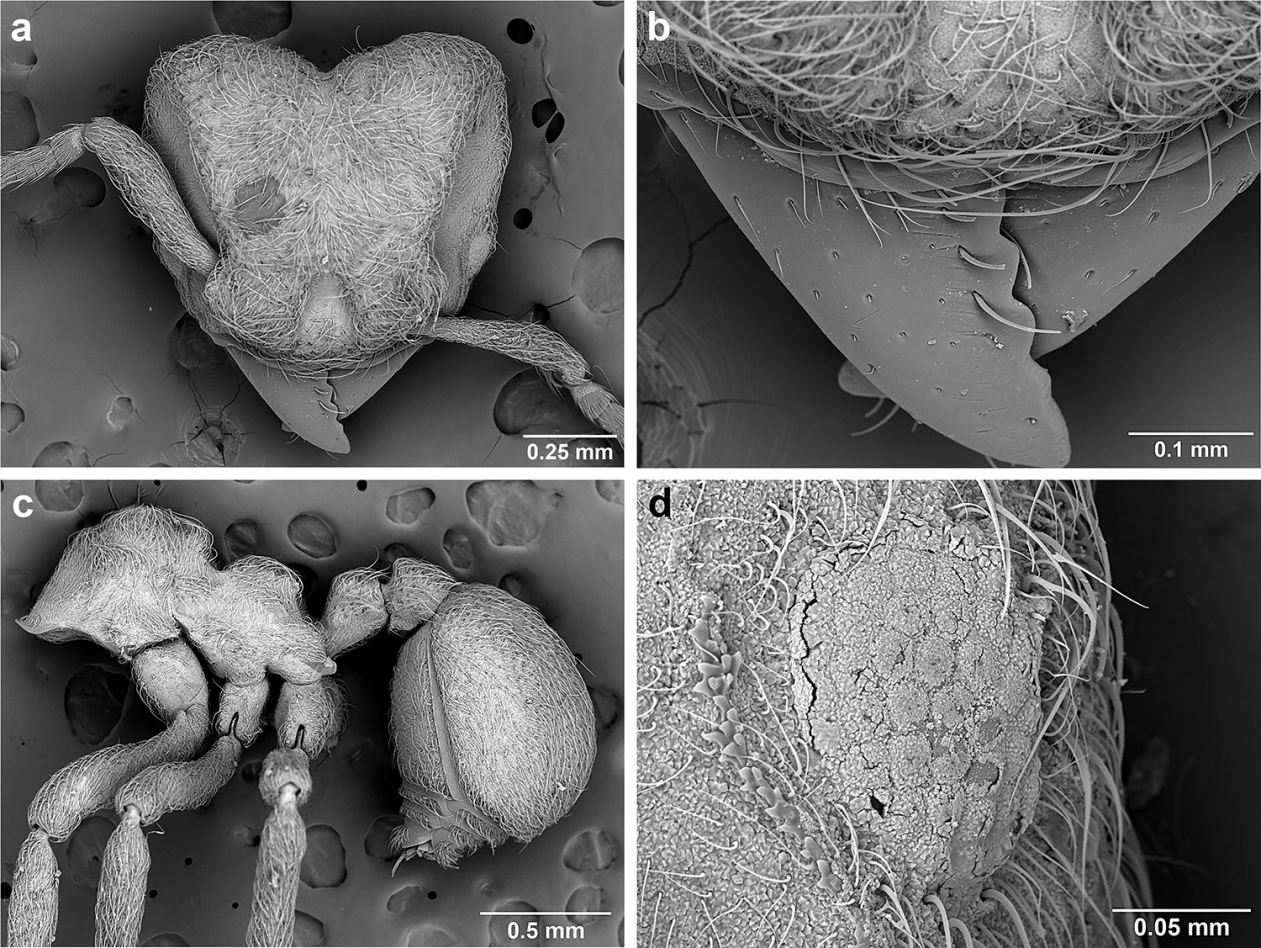
*S. opacus* worker (USNMENT01125331), SEM images. **a** Head, full-face view **b** mandibles **c** mesosoma and metasoma, lateral view **d** eye.

**Figure 45.**
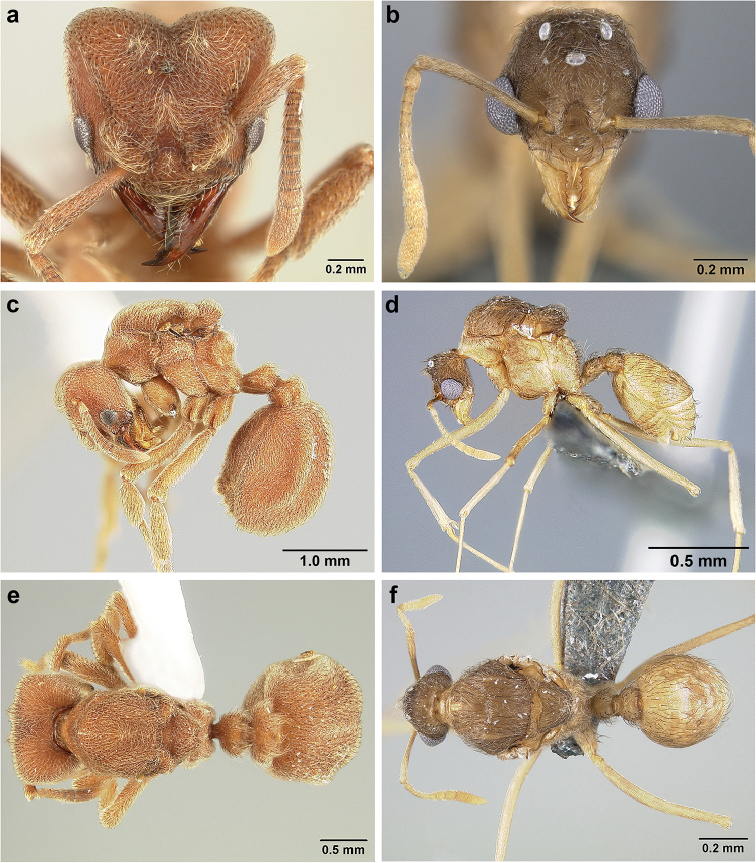
*S. opacus* queen and male; head, lateral profile, and dorsal view. Queen (USNMENT00305214) (**a, c, e**) Male (USNMENT01125314) (**b, d, f**).

**Figure 46.**
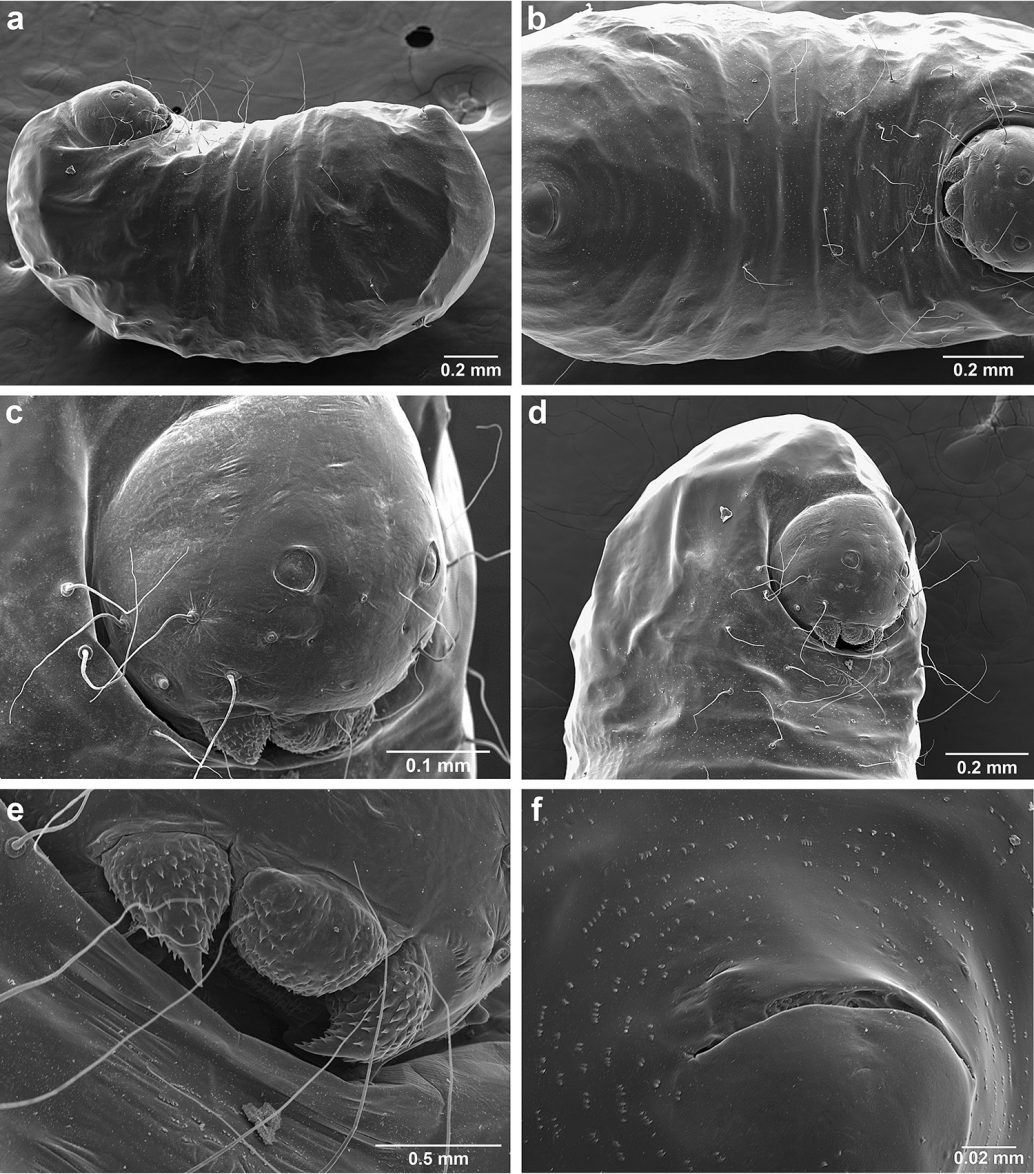
*S. opacus* larva (USNMENT01125317), SEM images. **a** Lateral view **b** ventral view **c** head, frontolateral view **d** head and thorax, frontolateral view **e** mouthparts **f** anal setae.

**Figure 47.**
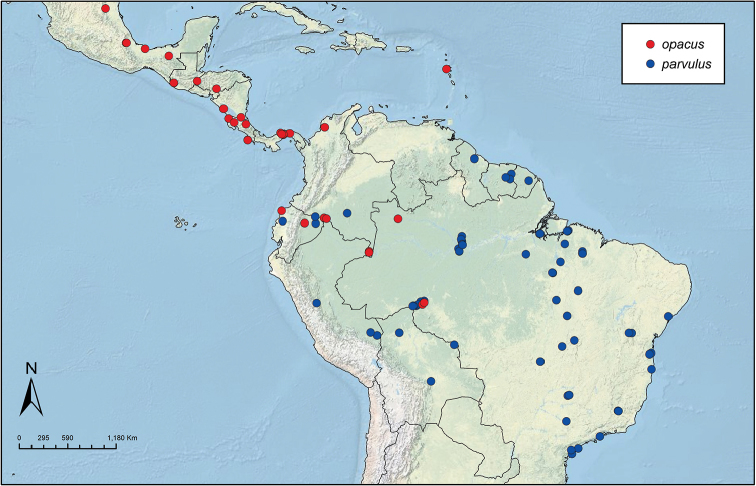
Distribution map of *S. opacus* and *S. parvulus*.

Figures 43 , 44 (Worker); Figure 45 (Queen and male); Figure 46 (Larva); Figure 47 (Map)

Sericomyrmex opacus Mayr, 1865: 84. Lectotype worker (here designated): (“Brazil”)* MEXICO, Veracruz, Córdoba, [18.8808, -96.9272], E. Norton (NHMW: 1w, CASENT0915956) *Paralectotypes*: Same data as lectotype: (NHMW: 1w, CASENT0915955) (USNM: 1w, USNM00924096).
Sericomyrmex aztecus = Forel, 1885: 363. **syn. n.** Type material examined: MEXICO, Veracruz, Orizaba, [18.85, -97.08], A. Forel (MSNG: 1w, CASENT0904987) (MHNG: 1w, CASENT0909368) (NHMW: 1w, USNMENT00924097).
Sericomyrmex diego = Forel, 1912: 192. **syn. n.** Type material examined: COLOMBIA, Magdalena, Don Diego, [11.23, -73.7], A. Forel (USNM: 1w, USNMENT00529165; 2w, USNMENT00921744) (BMNH: 2w, CASENT0901678) (MSNG: 2w, CASENT0904988; 1m, USNMENT00924098) (MHNG: 3w, CASENT0909369).
Sericomyrmex zacapanus = Wheeler, 1925a: 54. **syn. n.** Type material examined: GUATEMALA, Zacapa, [14.9722, -89.5306], 15 Dec 1911, W. M. Wheeler, (MCZ: 3w, USNMENT00924099; 2w, USNMENT00924100; 3w, USNMENT00924101) *For a discussion of the type locality of S. opacus see the notes section.

##### *S. opacus* worker diagnosis.

Small species; mandible smooth, glossy; posterior cephalic corner smoothly rounded; frontal lobe rectangular; eye small, often with at least partial white layer; mesosomal tubercles low, reduced; first gastral tergite with lateral carinae weakly developed, dorsal carinae absent.

##### *S. opacus* worker description.

Measurements in mm, range (lectotype): HWe 0.8–1 (0.93) HW 0.8–1 (0.95) HW1 0.78–1 (0.84) HW2 0.88–1.13 (0.96) HW3 0.54–0.74 (0.62) IFW1 0.54–0.73 (0.64) IFW2 0.16–0.28 (0.24) HL1 0.82–1 (0.9) HL2 0.6–0.9 (0.82) SL 0.58–1.08 (0.62) EL 0.12–0.18 (0.15) Om 6–10 (8) WL 0.99–1.3 (1.23) PL 0.2–0.33 (0.21) PPL 0.15–0.25 (0.18) GL 0.78–1 (0.87) HFL 0.68–1.02 (0.93) PW 0.50–0.72 (0.63) CI 95–106 (103) FLI 62–78 (69) SI 65–78 (67) OI 13–19 (16) CEI 5–19 (9) [N=68]

***Pilosity.*** Pubescence dense, often lighter than integument, appressed to decumbent. Hairs curved, darker in color at base, yellow to gray, appressed to suberect, mostly decumbent.

***Head.*** In full-face view almost equally broad and long (CI=100 ± 3), posterior corner smoothly rounded, posterior cephalic emargination relatively shallow (CEI=9 ± 2), gradually impressed. Vertexal impression and frontal tumuli faint. Mandibles with 7–8 teeth, dorsally smooth and glossy, finely transversely striate only along masticatory margin. Eye medium-sized (OI =16 ± 1), flat to slightly convex, 6–10 ommatidia across largest diameter, with (Figure 43a, 44d) or without (Figure 43b) thin white layer. Frontal lobe wide (FLI=70 ± 3), rarely triangular, mostly rectangular to trapeziform, posterior margin as long, or almost as long, as medial. Frontal carina usually incomplete, fading before reaching posterior cephalic corner. Antennal scape relatively short, never reaching posterior cephalic corner (SI=71 ± 2).

***Mesosoma.*** Mesosomal tubercles small, low and obtuse. Propodeal carinae low and feeble, sometimes serrate, sometimes reduced to just posterodorsal denticles.

***Metasoma.*** Petiole with two low, reduced denticles; postpetiole with two faint, short carina dorsally. First gastral tergite with lateral carinae weakly developed, dorsal carinae absent.

##### *S. opacus* queen description.

Measurements in mm, range: HWe 1.02–1.16 HW 1.05–1.20 HW1 1.10–1.25 HW2 1.20–1.30 HW3 0.72–0.85 IFW1 0.76–0.85 IFW2 0.24–0.33 HL1 1.09–1.12 HL2 0.98–1.00 SL 0.74–0.78 EL 0.20–0.24 Om 15–17 EW 0.08–0.10 WL 1.58–1.80 PL 0.34–0.48 PPL 0.24–0.28 GL 1.50–1.64 HFL 0.99–1.28 PW 0.90–0.96 FWg 4.85–5.17 HWg 3.60–3.64 CI 93–106 FLI 71–76 SI 64–73 OI 18–22 [N=6]

***Head.*** Mandible with 7–8 teeth, dorsally glossy and smooth, finely transversely striate only along masticatory margin. Preocular carina fading posterior to eye, 1–3 isolated, short, thin, supraocular carinae sometimes present, never reaching posterior cephalic corner. Eye large (OI=20 ± 1), convex, 15–17 ommatidia across largest diameter. Frontal lobe as in worker, antennal scape not reaching posterior cephalic corner.

***Mesosoma.*** Lateral pronotal tubercles low and obtuse. Scutum in dorsal view with notauli and median mesoscutal line reduced, parapsidal lines thin. Scutellum small and short, with posterior notch shallow to absent and with median impression sometimes separating scutellum in two lateral halves. Propodeal carinae low, each with low posterodorsal denticle.

***Metasoma.*** Petiole with two dorsal and two lateral low and obtuse denticles, best seen in frontodorsal view. Postpetiole with two short, low carinae dorsally and two low denticles laterally. First gastral tergite with lateral carinae well developed, dorsal carinae absent, anteromedian groove visible.

##### *S. opacus* male description.

Measurements in mm, range: HWe 0.66–0.74 HW 0.46–0.58 IFW1 0.20–0.27 IFW2 0.13–0.18 HL1 0.53–0.61 SL 0.49–0.62 EL 0.22–0.28 Om 19–28 EW 0.09–0.14 WL 1.20–1.44 PL 0.27–0.38 PPL 0.16–0.22 GL 0.96–1.12 HFL 1.20–1.50 PW 0.50–0.80 IOD 0.36–0.50 FWg 3.48–4.10 HWg 2.20–2.84 CI 115–125 FLI 30–38 SI 71–88 OI 29–39 [N=7]

Head in full-face view longer than broad (CI=121 ± 4). Eye large (OI=34 ± 3), convex, 19–28 ommatidia across largest diameter. Preocular carina fading posterior to eye, medially curved before fading. Notauli and mesoscutal line faint, parapsidal lines thin, groove between axillae smooth, sometimes weakly transversely costate. Propodeum without denticles or carinae. Petiole and postpetiole with lateral denticles very reduced, dorsal denticles absent.

##### *S. opacus* larva description.

Approximately eight setae on each side of dorsal and lateral body surfaces (i.e., approximately 16 total). Supra-antennal setae absent. Four genal setae on each side. Mandibular apical tooth undivided. Labial denticles not visible. First thoracic segment ventrally with multiple multidentate spinules, arranged in transverse rows. Numbers of ventral hairs: six on each thoracic segment, eight on the abdomen (not including anal setae). Single pair of sensilliform setae anterior to anal opening.

##### *S. opacus* geographic range.

Brazil, Central America, Colombia, Mexico. Map: Figure 47.

##### *S. opacus* notes.

The species most similar to *S. opacus* are *S. parvulus*, *S. saramama*, and, in Central America, smooth-mandibled populations of *S. amabilis*. Typical *amabilis* can be distinguished from *opacus* by their completely striate mandibles, triangular frontal lobes, and larger size. The distinction between *opacus* and smooth-mandibled *amabilis* is less obvious, but the frontal lobes, head shape, and size are still good indicators. An ameliorating factor for this difficulty is that, when sympatric, *amabilis* and *opacus* are very distinct; we have not encountered the smooth-mandibled *amabilis* variant sympatric with *opacus*, which might indicate character displacement. The queen of *S. opacus* can easily be separated from that of the sympatric *S. amabilis* by its smaller size, smooth mandibles (striate in *amabilis*), and usually less conspicuous notauli on the scutum. The main characters separating *S. opacus* from *saramama* are the shape of the frontal lobes (triangular in *saramama*) and the white layer over the eyes (absent in *saramama*). The *S. saramama* queen can easily be separated from the *opacus* queen by its striate mandibles (smooth in *opacus*).

*S. parvulus* can be distinguished from the typical *opacus* by having smaller, narrower, triangular frontal lobes; smaller overall size; and shorter frontal carinae, often fading well before reaching the posterior cephalic corners. Separating non-typical representatives of *opacus*, which may also have reduced frontal lobes, from *parvulus* is difficult. Also, it is very difficult to separate queens of *opacus* and *parvulus*. The *parvulus* queen is slightly smaller and lacks the faint supraocular carinae; however, these carinae are absent in some *opacus* queens as well. The region of overlap of the known distributions of these two species is limited, so geographic origin can aid in species identification (Figure 47).

*S. opacus* is morphologically variable across its geographic range. This variation is correlated with patterns in the molecular phylogeny (Suppl. material 1) as well as with geography. The three main subspecific geographical and molecular subclades, all reciprocally monophyletic, are: Central American (population *opacus* 1), North Colombian (population *opacus* 2), and South Colombian and West Brazilian (population *opacus* 3). The most pronounced variation within *opacus* occurs in the shape of the frontal lobes and in the eyes. The typical *S. opacus* has rectangular frontal lobes and the eyes covered with a thin white layer. In populations of *opacus* 1 there are occasional, rare individuals with smaller, almost triangular lobes and eyes lacking the white layer. These rare, odd specimens are also smaller in size, so these atypical character states could be correlated with size (e.g., in nanitic workers). The specimens from *opacus* 2 populations, from Northern Colombia, are fairly uniform, typical representatives of the species. This uniformity, however, may be an artifact of our small sample size (20 individuals). Most *opacus* 2 specimens were collected in pitfall traps, so no nest series were available for evaluating within-nest variation. The specimens from the *opacus* 3 population all have eyes lacking the white layer and the frontal lobes are often (but not always) more triangular than rectangular. The number of samples available from this population is also low (19 specimens examined compared to 97 for *opacus* 1) and there are a few specimens from this population that are morphologically typical, including individuals with rectangular frontal lobes. The principal component analysis of the morphological data for just these three *opacus* populations (Figure 5f) shows no separation of the three populations along the two main axes. With regard to the high degree of morphological variation in *S. opacus*, one possibility well worth considering is that *opacus* and *parvulus* (which are similar in size and which overlap in distribution in the area where *opacus* 3 occurs) can hybridize (Figure 47). If so, then the specimens that are difficult to identify as either *parvulus* or *opacus* could be *opacus*-*parvulus* hybrids. Separate PCA analysis of only *parvulus* and *opacus* indicates a large amount of overlap (Figure 5b) along the two main axes. The data currently available are insufficient for recognizing the three *opacus* subclades as species and we believe that the observed pattern (especially in *opacus* 3) is likely an artifact of our low sample sizes. It is entirely possible that further sampling, especially of whole-nest series, might reveal that *S. opacus* actually consists of multiple species and/or that *opacus* hybridizes with *parvulus*.

*S. opacus* *type locality.* The original description of *S. opacus* by G. L. Mayr in 1865, which is also the original description of the genus *Sericomyrmex*, lists “Brasilien” as the type locality. Based on the route of the Novara-Expedition, which is the expedition from which Mayr’s specimens supposedly originated, the Brazilian type locality is most likely Rio de Janiero (Mayr, 1865). However, the locality labels of the type specimens (CASENT0915955, CASENT0915956, USNMENT00924096) of *Sericomyrmex opacus* that we studied indicate “Mexico, Cordoba,” and “Norton” as the collector. The labels look original when compared to the labels of other Mayr type specimens, both in terms of handwriting and in resemblance to the labels of other specimens collected by Norton. Indeed, Mayr described other species based on specimens collected by E. Norton in Mexico, so he clearly had access to Norton’s Mexican material. Importantly, the type specimen of *S. opacus* is identical to the type specimen of *S. aztecus*, a Mexican species described by Forel (1885), which in turn is identical to numerous specimens collected in Mexico and elsewhere in Central America. *S. aztecus* is the name most commonly applied to such specimens. Given these facts, the most likely explanation is that the locality of the type specimen of *S. opacus* (“Brasilien”) given in the original description is incorrect and that the locality labels affixed to the syntype specimens are correct. The alternative explanation is that the published locality is instead correct, the specimen labels are incorrect, and that the type specimen of *S. opacus* originated in Brazil. We judge this alternative to be highly unlikely, because *S. opacus* (under which we have synonymized *S. aztecus*, *S. diego*, and *S. zacapanus*) does not occur anywhere near Rio de Janeiro (Figure 47).

##### Synonymies.

The type specimens of *S. aztecus*, *S. diego*, and *S. zacapanus* all possess typical *opacus* morphology, including the rectangular frontal lobes. In his description of *zacapanus* Wheeler (1925a) mentions that it is smaller in size than *diego*, but our measurement data indicate that the cited difference is very small (Suppl. material 2: Table S2a) and falls entirely within the size variation of the species. In his description of *aztecus* Forel (1912) indicates that it has a relatively smooth petiole, whereas in *opacus* dorsal denticles are present. Variation in petiolar and postpetiolar carinae and denticles is not diagnostic for species of *Sericomyrmex*, and those characters often vary within a single colony.

##### *S. opacus* material examined.

**BRAZIL**: **Amazonas**: São Gabriel de Cachoeira (Uapés), [-0.1237, -67.0476], 120m, 23 Aug 1992, T. R. Schultz; **Rondônia**: Ilha do Bufalo, km 0.5, -9.2656, -64.2125, 90m, 19 Jan 2014, I. O. Fernandes; Jaci MD, km 3, -9.2656, -64.2125, 94m, 22 Jan 2014, I. O. Fernandes; Jaci-Paraná, km 2, -9.2656, -64.2125, 94m, 6 Jun 2012, I. O. Fernandes; Novo Modulo, Jaci-Paraná, km4, -9.4630, -64.3911, 122m, 24 Jan 2014, I. O. Fernandes; **COLOMBIA**: **Amazonas**: PNN Amacayacu Matamata, -3.6833, -70.25, 150m, 20 May 2000, A. Parente; PNN Amacayacu, -3.8103, -70.2662, 88m, 7 Oct 2007, J. Sosa-Calvo, J. Rodriguez; **Bolívar**: PNN Los Colorados, Villa Roca, 9.9, -75.1166, 180m, 26 May 2001, E. Deulufeut; PNN Los Colorados, La Yaya, 9.9, -75.1166, 280m, 21 Jul 2001, E. Deulufeut; PNN Los Colorados, Alto el Mirador, 9.9, -75.1166, 400m, 11 Apr 2001, E. Deulufeut; PNN Los Colorados, Venado, 9.9, -75.1166, 320m, 1 Jan 2001, E. Deulufeut; **Putumayo**: PNN La Paya Cabaña Chagra, -0.1166, -74.9333, 320m, 1 May 2002, R. Cobete; PNN La Paya Cabaña La Paya Chagra, -0.0333, -75.2, 330m, 26 Feb 2002, R. Cobete; PNN La Paya Cabaña La Paya, -0.0333, -75.2, 330m, 2 Jul 2002, R. Cobete; PNN La Paya Cabaña Viviano, -0.1166, -74.9333, 320m, 26 May 2002, A. Morales; PNN La Paya Río Caucaya, -0.1166, -74.9333, 330m, 15 Oct 2001, R. Cobete; **COSTA RICA: Guanacaste**: Canas, Finca Pacifica, [10.42, -85.10], 16 Jul 1986, S. B. Peck; Hacienda La Pacifica, nr. Canas, 10.24, -83.80, 50m, 1 May 1979, P. S. Ward; PN Santa Rosa, [10.8378, -85.7051], 14 Jun 1995, U. G. Mueller; **Puntarenas**: Osa Peninsula, Corcovado, Sirena Station, Pavo trail, [8.51, -83.60], 2 Jun 1992, T. R. Schultz; **ECUADOR: Esmeraldas**: 10 km S Atacames, [0.7755, -79.8462], 205m, 7 Nov 1987, C.R.F. Brandão, C.D. Bastidas; **Los Ríos**: Río Palenque Research Station, [-0.583, -79.367], 20 Dec 1980, S. Sandoval; **GUATEMALA: Retalhuleu**: El Asintal, 14.6524, -91.73901, 670m, 30 Jul 2013, K. Delgado; Nuevo San Carlos, 14.6388, -91.72193, 585m, 9 Nov 2008, K. Delgado; **HONDURAS: Copán**: Copán Ruinas, 14.8379, -89.1428, 629m, 4 Jan 2008, C. Rabeling; **Francisco Morazán**: Esc. Zamorano, 14.0134, -87.0076 ±25m, 800m, 19 May 2009, J. T. Longino; **MEXICO: Chiapas**: 8km SE Salto de Agua, 17.5143, -92.2949 ±200m, 70m, 16 Jun 2008, M. G. Branstetter; **San Luis Potosí**: Cuesta de los Cedros, 36 km E of Ciudad del Maíz, [22.3889, -99.2515], 685m, 12 Jun 1962; **Veracruz**: Ocotal Chico, [18.2588, -94.8619], 579m, 26 Jun 1963, G. N. Ross; **NICARAGUA: Masaya**: Masatepe, vic. San Marcos, Cafetal San José del Llano, 11.917, -86.25, 485m, 23 Jun 1992, T. R. Schultz; **Río San Juan**: Río San Juan, Isla de Diamante, [10.9794, -84.3415], 9 Oct 1994, J. P. Caldwell; **PANAMÁ: Colón**: Gamboa, PN Soberanía, Pipeline Rd. Km 6, 9.08, -79.66, 24 Apr 1996, T. R. Schultz, U. G. Mueller; San Lorenzo Forest R1, 9.2833, -79.9666, 30 Dec 2004, A. Dejean, J. Orivel, B. Corbara, H. Aberlenc, M. Leponce; **Panamá**: Barro Colorado Island, Canal Zone, 9.15, -79.84, 1 Nov 1941, J. Zetek; El Llano-Carti Suitupo Rd, ca. 6km ex El Llano, [9.22, -78.97], 27 Apr 1996, T. R. Schultz, U. G. Mueller, S. Rehner.

#### Sericomyrmex parvulus

Taxon classificationAnimaliaHymenopteraFormicidae

Forel, 1912

**Figure 48.**
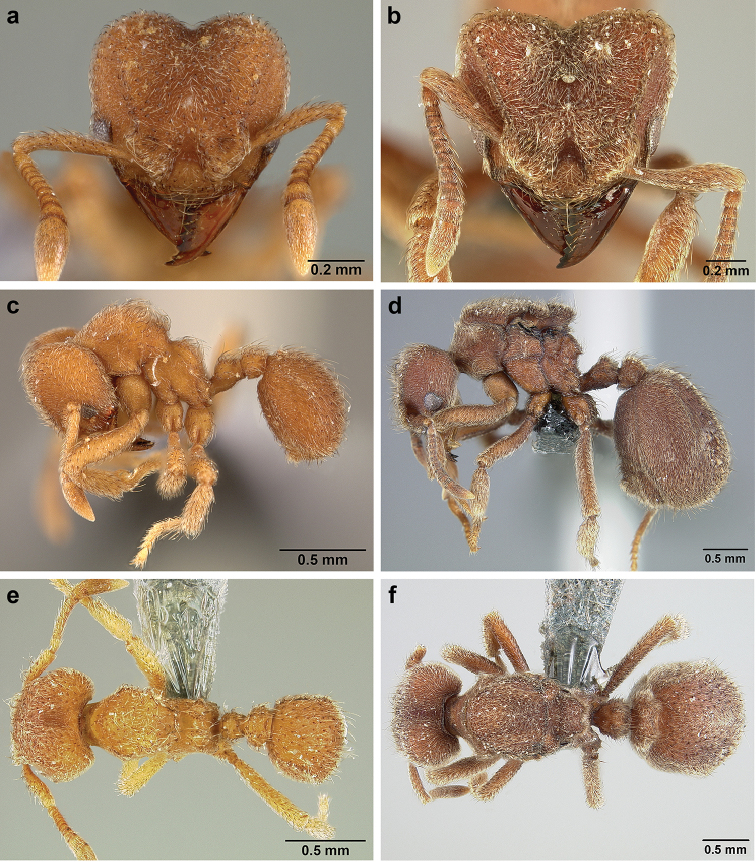
*S. parvulus* worker and queen; head, lateral profile, and dorsal view. Worker (USNMENT00446157) (**a, c, e**). Queen (USNMENT01125594) (**b, d, f**).

**Figure 49.**
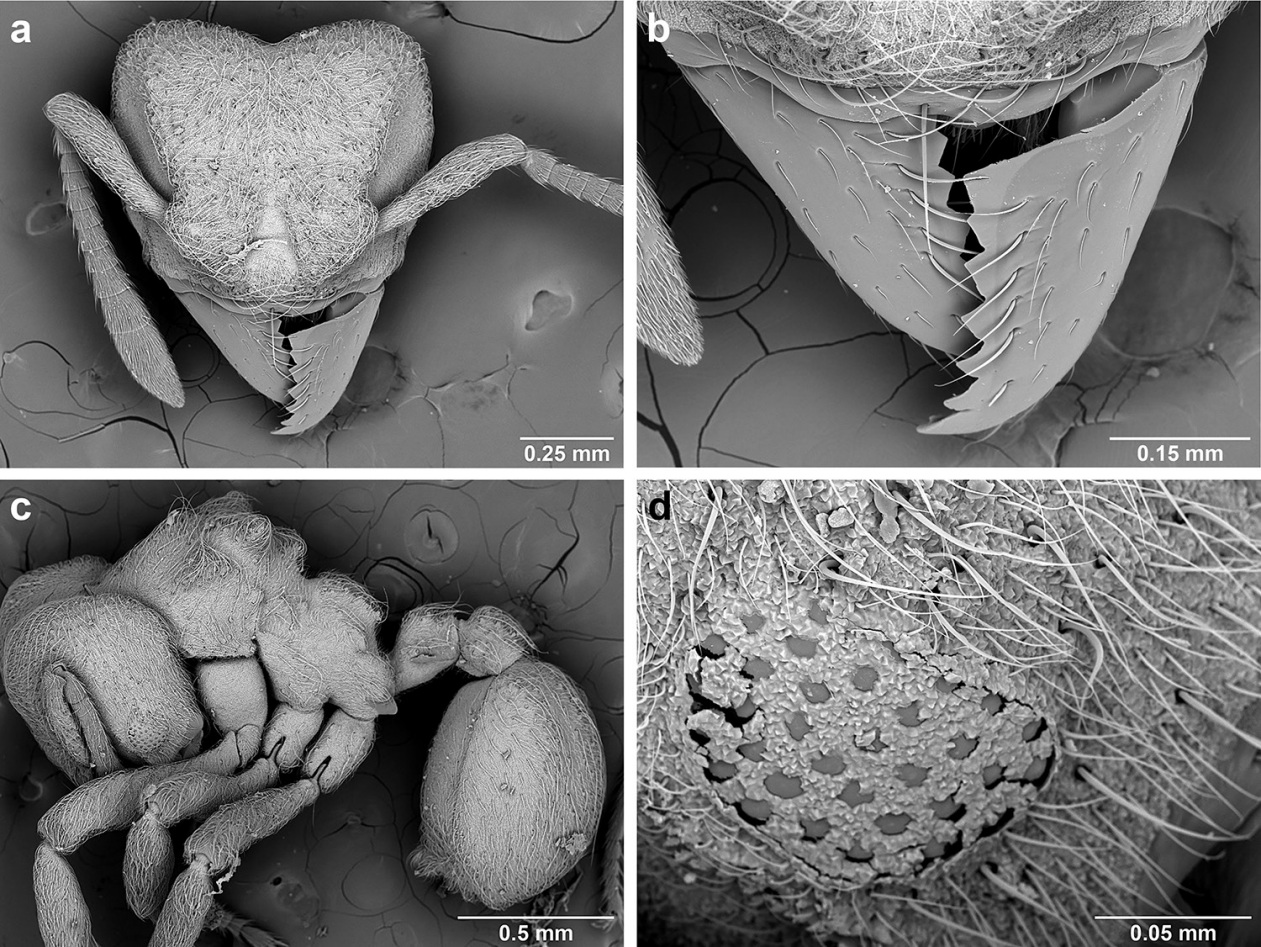
*S. parvulus* worker (USNMENT01125593), SEM images. **a** Head, full-face view **b** mandibles **c** mesosoma and metasoma, lateral view **d** eye.

**Figure 50.**
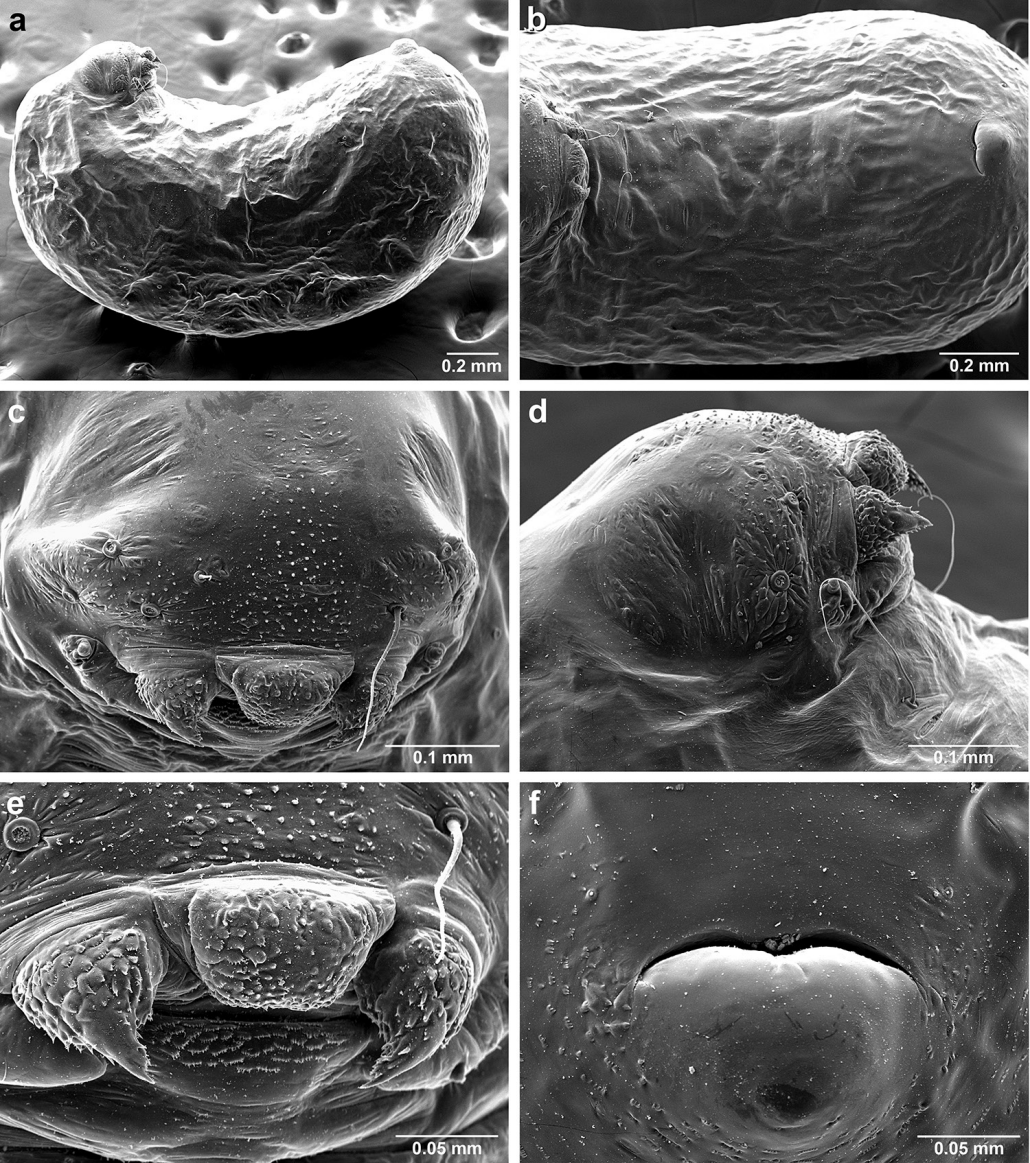
*S. parvulus* larva (USNMENT01125592), SEM images. **a** Lateral view **b** ventral view **c** head, frontodorsal view **d** head, lateral view **e** mouthparts **f** anal setae.

Figures 48 , 49 (Worker); Figure 49 (Queen); Figure 50 (Larva); Figure 47 (Map)

Sericomyrmex parvulus Forel, 1912: 193. Lectotype worker (here designated): BRAZIL, Pará, [-4, -53] C. Emery (MHNG: 1w, USNM00445579, bottom specimen). *Paralectotype*: same data as lectotype (MHNG: 1w, USNM00445579, top specimen).
Sericomyrmex myersi = Weber, 1937: 400. **syn. n.** Type material examined: SURINAME, Nickerie, Upper Courantyne River, JGM5931, 29 Dec 1935, J. G. Mayers (MCZ: 1w, USNMENT00924107).

##### *S. parvulus* worker diagnosis.

Small species; posterior cephalic corner smoothly rounded; frontal lobe triangular, small, narrow; frontal carina faint, incomplete; mesosomal tubercles small, low, first gastral tergite with lateral carinae weakly developed, dorsal carinae absent.

##### *S. parvulus* worker description.

Measurements in mm, range (lectotype): HWe 0.66–0.9 (0.9) HW 0.66–0.93 (0.93) HW1 0.6–0.93 (0.84) HW2 0.68–1.03 (0.93) HW3 0.48–0.8 (0.6) IFW1 0.42–0.65 (0.62) IFW2 0.15–0.28 (0.26) HL1 0.62–0.9 (0.88) HL2 0.58–0.82 (0.8) SL 0.48–0.72 (0.64) EL 0.11–0.15 (0.15) Om 6–9 WL 0.74–1.23 (1.23) PL 0.16–0.34 (0.25) PPL 0.13–0.24 (0.16) GL 0.6–0.9 (0.83) HFL 0.65–0.99 (0.92) PW 0.46–0.64 (0.62) CI 94–106 (103) FLI 60–75 (69) SI 64–78 (71) OI 13–19 (17) CEI 5–12 (8) [N=55]

***Pilosity.*** Pubescence dense, lighter than integument, appressed to decumbent. Hairs moderately thick, relatively sparse, often curved, yellow to gray, appressed to suberect.

**Head.** In full-face view evenly broad and long (CI=102 ± 3), posterior corner smoothly rounded, posterior cephalic emargination shallow (CEI=9 ± 2), gradually impressed. Vertexal impression faint, frontal tumuli barely visible. Mandible with 7–8 teeth, dorsally smooth and glossy, finely transversely striate only along masticatory margin. Eye medium-sized (OI =16 ± 1), flat to slightly convex, 6–9 ommatidia across largest diameter, in some specimens eyes partially covered with white layer (Figure 6l), in others eyes without white layer. Frontal lobe triangular, relatively small and narrow (FLI=70 ± 3), posterior margin shorter than medial. Frontal carina straight to slightly curved laterally, incomplete, weak, fading before reaching posterior cephalic corner. Antennal scape relatively short, not reaching posterior cephalic corner (SI=71 ± 3).

***Mesosoma.*** Mesosomal tubercles low and obtuse. Propodeal carinae low and weak, with small posterodorsal denticles.

***Metasoma.*** Petiole with two low, reduced dorsal denticles; postpetiole with two faint, short dorsal carina; both best seen in dorsolateral view. First gastral tergite with lateral carinae weakly developed, dorsal carinae faint or absent.

##### *S. parvulus* queen description.

Measurements in mm, range: HWe 0.98–1.05 HW 1–1.08 HW1 1–1.13 HW2 1.08–1.22 HW3 0.74–0.8 IFW1 0.7–0.78 IFW2 0.24–0.28 HL1 0.95–1.08 HL2 0.88–0.95 SL 0.64–0.7 EL 0.21–0.24 Om 14–15 EW 0.08–0.08 WL 1.56–1.65 PL 0.34–0.48 PPL 0.2–0.25 GL 1.4–1.58 HFL 1–1.18 PW 0.82–0.92 CI 95–103 FLI 72–76 SI 66–70 OI 21–23 [N=4]

***Head.*** Mandible with 7–8 teeth, dorsally glossy and smooth, finely transversely striate only along masticatory margin. Preocular carina fading posterior to eye. Eye large (OI=22 ± 1), mildly convex, 14–15 ommatidia across largest diameter. Frontal lobe more robust than in worker, antennal scape not reaching posterior cephalic corner.

***Mesosoma.*** Lateral pronotal tubercles very low. Scutum in dorsal view with notauli and median mesoscutal line absent or very faint. Parapsidal lines faint, slightly curved. Axillae small, groove separating axillae from scutellum smooth. Scutellum short in dorsal view, narrowing posteriorly, posterior margin with V-shaped notch, notch sometimes continuing into median impression that divides scutellum in two lateral parts. Propodeal denticle low, obtuse, laterally flattened, diverging posteriorly in dorsal view.

***Metasoma.*** First tergite of gaster with lateral carinae well developed, dorsal carinae absent or weak, anteromedian groove shallow.

##### *S. parvulus* male.

Unknown.

##### *S. parvulus* geographic range.

Bolivia, Brazil, Colombia, Ecuador, French Guiana, Guyana, Peru, Suriname. Map: Figure 47.

##### *S. parvulus* larva.

Lateral and dorsal surfaces without any setae. Supra-antennal setae absent. Four genal setae on each side. Mandibular apical tooth undivided. Labial denticles either absent or small number of denticles present anterior to sericteries. Thoracic segment 1 (T1) ventrally with multidentate spinules. Number of ventral setae: T1, T2, and T3 with two setae each, abdomen without setae (not including anal setae). Single pair of sensilliform setae anterior to anal opening.

##### *S. parvulus* notes.

The aptly named *S. parvulus* is the smallest *Sericomyrmex* species. In the regions where their distributions overlap (Figure 47), *parvulus* is most easily mistaken for *S. opacus*. In general *opacus* is larger, with wider, rectangular frontal lobes. Similarly, the *S. opacus* queen can be separated from the *parvulus* queen by its slightly larger size and by the presence of supraocular eye carinae in some specimens (absent in *parvulus*). The queen of *S. saramama* is similar in size but can be recognized by its striate mandibles (smooth in *parvulus*).

Within-species morphological variation in *S. parvulus* includes the frontal carinae (typically incomplete and faint, but complete and stronger in some populations), eyes (sometimes covered with a white layer, but sometimes not), and the general robustness of denticles and tubercles on the mesosoma and metasoma.

In the populations of *S. opacus* from southern Colombia and northwestern Brazil (clade *opacus* 3, see discussion in notes for *S. opacus* and Suppl. material 1), some of the workers have small, triangular frontal lobes, making them easy to mistake for *parvulus*. They are usually larger than the typical *parvulus* worker, but given the overlap in size between the two species, they could be mistaken for larger *parvulus* workers. It is possible that these intermediate individuals are hybrids and that the molecular and morphological differences between the Colombian and Brazilian populations of *opacus* are a consequence of hybridization and introgression with *parvulus* (see also discussion in notes for *S. opacus*).

*Synonymies.* The holotype specimen of *S. myersi* we examined is almost identical to the *parvulus* lectotype, except that the tubercles on the mesosoma are slightly more distinct in *myersi*, but this difference falls well within the variation observed in *parvulus*. In his description of *myersi* Weber (1937) distinguishes it from *S. lutzi* and *S. harekulli* by its much smaller size but does not compare it to *S. parvulus*.

##### *S. parvulus* material examined.

**BOLIVIA: Beni**: Cavinas, [-12.5311, -66.9146], 30 Oct 1921, W. M. Mann; **Santa Cruz**: 10km NW Terevinto, -17.6667, -63.45, 380m, 9 Dec 1993, P. S. Ward; 35 km SSE Flor de Oro, -13.8333, -60.8667, 450m, 27 Nov 1993, P. S. Ward; **BRAZIL: Amazonas**: Embrapa Amazônia Ocidental, 30 km N Manaus, -2.89824, -59.9903, 4 Sep 2006, C. Rabeling; Manaquiri, Br 319, km100, [-3.6829, -60.32], 10 Oct 2010, F. Baccaro; Manaus, Br 174 Km 46-EEST INPA, [-2.58, -60.03], 83m, 21 Feb 1991, A. Y. Harada; Manaus, Dimona, INPA,100 ha plot, -2.3833, -60.1, 80m, 6 Jan 2009, J. Sosa-Calvo; Manaus, Rs2303, [-2.96, -59.93], 29 Sep 1993, A. B. Casimiro; Pres. Figueiredo, [-2.02, -60.02], 27 Jan 1994, Queiroz; São Gabriel de Cachoeira, [-0.1237, -67.0476], 120m, 23 Aug 1992, T. R. Schultz; **Bahia**: Ilhéus, Itabuna, CEPEC area Zoolog, km 22, [-14.7, -39.2], 1 Oct 1986, J. H. C. Delabie; Itabuna, Ferradas A27, -14.8258, -39.4044, 21 Sep 2000, J. R. M. Santos; Lençóis, -12.56151, -41.36942 ±5m, 487m, 10 Nov 2008, J. Sosa-Calvo; Lençóis, trevo, [-12.55, -41.6833], 30 Mar 2001, J. R. M. Santos; Porto Seguro, -16.3925, -39.1694; **Goiás**: Colinas do Sul, Serra da Mesa, -14.0166, -49.02, 2 Dec 1995, B. H. Dietz, Campaner; Faz. Pau Brasil, nr. Jussara, -15.5835, -51.3966 ±6m, 305m, 30 Sep 2008, J. Sosa-Calvo, T. R. Schultz; **Maranhão**: Bom Jardim, REBIO Gurupi Parcela 01 08, -3.9258, -46.7712, 20 Sep 2014, A. Y. Harada; Centro Novo Maranhão, REBIO Gurupi, Parcela 02, -3.682111, -46.7798, 18 Jul 2014, A. Y. Harada; **Minas Gerais**: Uberlândia, Panga, -19.18314, -48.40141, 813m, 18 Oct 2012, A. Ješovnik; Uberlândia, Panga, -19.10557, -48.23849, 810m, 22 Sep 2008, J. Sosa-Calvo; Uberlândia, Panga, -19.1666, -48.3833, 790m, 22 Sep 2008, T. R. Schultz; Viçosa, -20.7833, -42.8333, 30 Oct 2014, R. Jesus, J. Chaul; **Pará**: Belém, IPEAN APEG, [-1.4373, -48.4706], 19 Jul 1971, I. B. de Almeida; Belém, Parque R.A., [-1.4585, -48.4372], R.C.G.; Belém, Utinga Forest Preserve, -1.4174, -48.4288 ±5m, 45m, 3 Feb 2009, J. Sosa-Calvo; Marituba, CEPLAC Station, [-1.3666, -48.3333], 16 Oct 2004, J. R. M. Santos; Melgaço, Caxiuanã, ECFPn, -1.7248, -51.4230, 20 Oct 2007, A. Y. Harada; Nova Ipixuna, Fazenda Bom Retiro, Parcela 02, -4.8412, -49.218, 12 Apr 2012, M. Tavares, A. Palmeira; Parauapebas, FL Nacional de Carajás, Parque Zoobotânico, -6.06292, -50.05712, 626m, 3 Oct 2014, A. Ješovnik; Tailândia, Fazenda Santa Marta, Juruá Florestal, [-3.0167, -64.2667], 28 May 2002; **Rondônia**: Ilha do Bufalo, km 0.5, subparcela 250, -9.0818, -64.2125, 90m, 26 Oct 2013, I. O. Fernandes; Ilha Pedras, km 4, subparcela 100, -9.1512, -64.578, 86m, 25 Oct 2013, I. O. Fernandes; Jaci-Paraná, km 4, subparcela 150, -9.2656, -64.2125, 94m, 26 Nov 2011, I. O. Fernandes; Rio Jamari, São Pedro, [-10.2, -63.25], 11 Jun 1960, OP Fora Hini ; **São Paulo**: Cananéia, Ilha do Cardoso, -28.0968, -47.9298, 24 Dec 2002, R. R. Silva, C. R. F. Brandão, C. Scott; Faz. Itaquerê, Nova Europa, [-21.7838, -48.5578], 2 Dec 1963, K. Lenko; Jacupiranga, [-24.7055, -48.0167], 1 Nov 1963, F. Plaumann; Jureia-Itantins, -24.5442, -47.235; **Sergipe**: Areia Branca, PN Serra de Itabaiana, -10.765, -37.3326, 19 May 2003, R. R. Silva, B. H. Dietz, L. S. Ferreira; **Tocantins**: Araguacema, -8.9886, -49.6675, 16 Nov 2005, R. R. Silva, R. M. Feitosa; Goiatins, -7.9793, -47.2507, 3 May 2005, R. R. Silva, B. H. Dietz; Paraná, Serra da Contenda, -13.3576, -47.6756, 13 Oct 2004, R. R. Silva, B. H. Dietz; Porto Nacional, Faz. Alto Paraíso, Transecto 1, -10.7089, -48.4680, 30 Sep 2001, R. R. Silva, N. L. Albuquerque; **COLOMBIA: Amazonas**: PNN Amacayacu Matamata, -3.6833, -70.25, 150m, 17 Jan 2001, A. Alvarado; **Caquetá**: Puerto Solano, PNN Serranía de Chiribiquete, Río Cuñaré, 0.5, -72.631, 250m, 1 Nov 2000, E. Gonzales; **ECUADOR: Manabi**: 73km NE Chone, B364, [-0.363, -79.739 ±10000m], 300m, 12 Jun 1976, S., J. Peck; **Orellana**: Tiputini Biodiversity Station, -0.6333, -76.1333, 10 Feb 2003, K. T. Ryder Wilkie; **Sucumbíos**: Reserva Faunistico Cuyabeno, 0.1167, -76.1833, 1 Nov 1994, J. P. Caldwell; **FRENCH GUIANA: Cayenne**: Régina, Nouragues Field Station, intersect. trails 16&L, 4.0849, -52.6771, 95m, 4 Aug 2005, T. R. Schultz; **GUYANA: Cuyuni-Mazaruni**: Calm Water Creek, along Essequibo River, nr. Bartica, 6.466667, -58.65, 26 Sep 2002, J. S. La Polla; **PERU: Huánuco**: Monson Valley, Tingo Maria, -9.3167, -76.0167, 600m, 10 Nov 1954, E. I. Schlinger, E. S. Ross; **Madre de Dios**: Tambopata Reserve, -12.8187, -69.3636, 224m, 1 Aug 2012, A. Ješovnik; **SURINAME: Sipaliwini**: Lely Mountains, 4.2529, -54.7561, 619m, 26 Oct 2005, J. Sosa-Calvo, R. Badal; Nassau Mountains, 4.8172, -54.6067, 514m, 3 Nov 2005, J. Sosa-Calvo.

#### Sericomyrmex radioheadi sp. n.

Taxon classificationAnimaliaHymenopteraFormicidae

http://zoobank.org/F29FE139-E3F8-4806-B977-2EF79FB566F7

**Figure 51.**
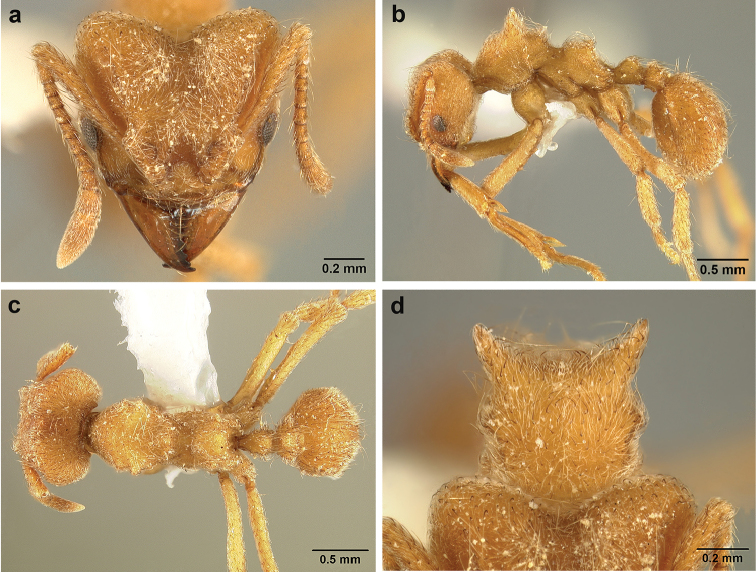
*S. radioheadi* worker (USNMENT00924059). **a** Head **b** lateral profile **c** dorsal view; and **d** frontodorsal view of mesonotal tubercles.

**Figure 52.**
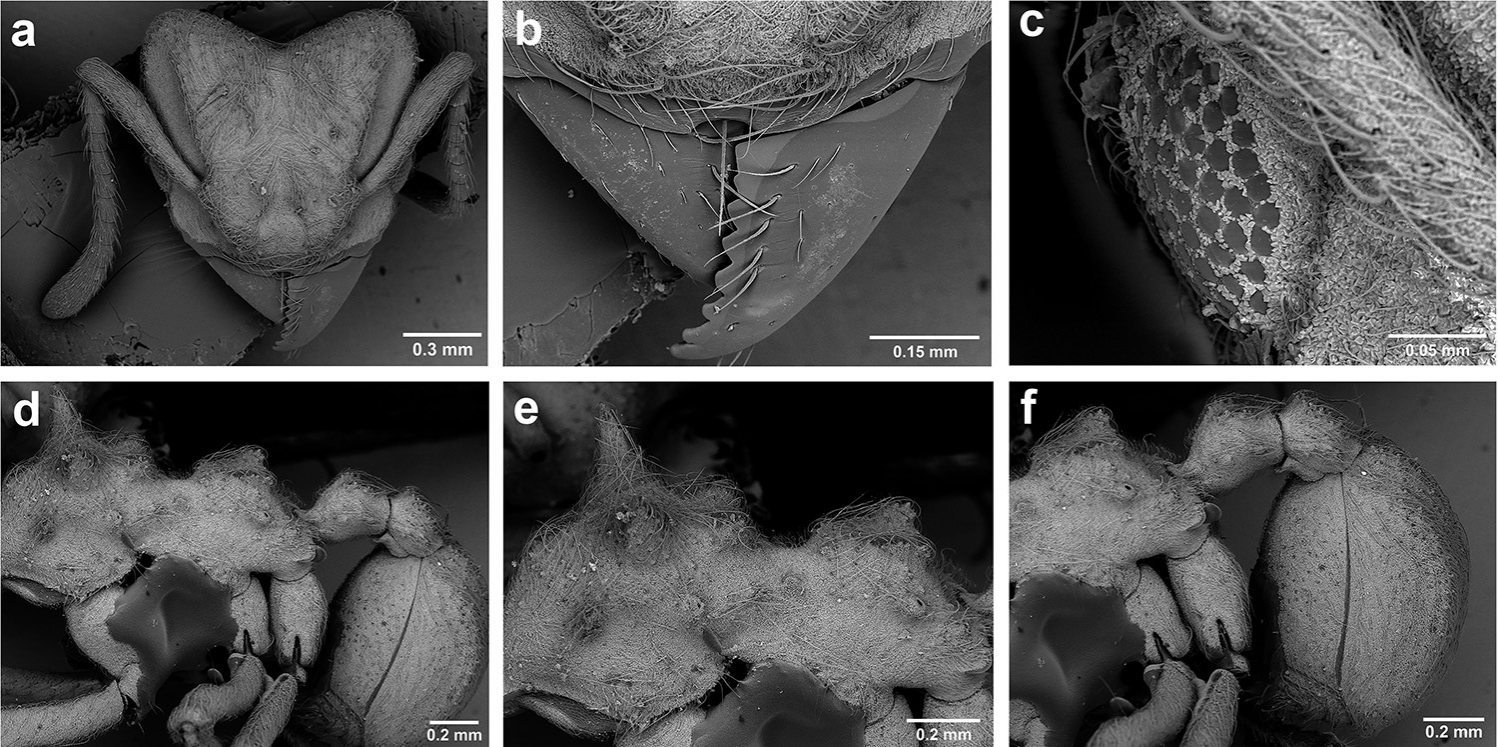
*S. radioheadi* worker (USNMENT00924061), SEM images. **a** Head, full-face view **b** mandibles **c** eye **d** mesosoma and metasoma, lateral view **e** mesosoma (detail), lateral view **f** propodeum and metasoma, anterolateral view.

Figures 51 , 52 (Worker); Figure 31 (Map)

##### Type material.

*Holotype worker*: VENEZUELA, Amazonas, 10 km N of San Carlos de Río Negro, [2.0164, -67.0599] Jul-Aug 1978, K. Clark (MCZ: 1w, USNMENT00924059). *Paratypes*: same data as holotype (USNM: 2 w: USNMENT00924060; 2w, USNMENT00924063) (CASC: 2w, USNMENT00924061) (MZSP: 2w, USNMENT00924062).

##### *S. radioheadi* worker diagnosis.

Medium-sized species; mandible dorsally smooth; frontal lobe triangular, narrow, directed anterad; antenna long, antennal scape reaching posterior cephalic corner; posterior cephalic emargination deep; lateral mesonotal tubercles sharp and long; first gastral tergite with lateral carinae distinct, dorsal carinae faint or absent.

##### *S. radioheadi* worker description.

Measurements in mm, range (holotype): HWe 1–1.08 (1.02) HW 1–1.08 (1.04) HW1 0.93–1 (1) HW2 1.08–1.15 (1.1) HW3 0.64–0.73 (0.72) IFW1 0.61–0.66 (0.62) IFW2 0.24–0.27 (0.24) HL1 1–1.08 (1) HL2 0.84–0.9 (0.88) SL 0.75–0.84 (0.8) EL 0.15–0.18 (0.15) Om 9–12 (9) WL 1.35–1.43 (1.36) PL 0.24–0.34 (0.29) PPL 0.2–0.25 (0.25) GL 0.92–1.02 (0.93) HFL 1.18–1.3 (1.25) PW 0.64–0.75 (0.65) CI 98–103 (102) FLI 59–62 (61) SI 75–82 (78) OI 15–18 (15) CEI 12–17 (12) [N=9]

***Pilosity.*** Pubescence dense, appressed to decumbent, light yellow. Hairs curved, darker in color at base, yellow to gray, appressed to suberect, mostly decumbent.

***Head.*** In full-face view evenly broad and long (CI=101 ± 2), posterior corner acute, posterior cephalic emargination deep (CEI=14 ± 2), gradually impressed. Vertexal impression distinct, frontal tumuli barely visible. Mandible with 7–8 teeth, dorsally smooth and glossy, finely transversely striate only along masticatory margin, striation sometimes faint. Eye medium-sized (OI =16 ± 1), mildly convex, without white layer, 9–12 ommatidia across largest diameter. Frontal lobe triangular, narrow (FLI=61 ± 1), posterior margin shorter than medial, lateral margin long, sometimes mildly convex. Frontal carina straight to slightly curved laterally, complete, reaching posterior cephalic corner. Antennal scape long (SI=77 ± 2), reaching posterior cephalic corners (Figure 3SI).

***Mesosoma.*** Lateral pronotal tubercles short, lateral mesonotal tubercles sharp and long (Figure 51d, 52e); posterior mesonotal tubercles low and obtuse. Propodeal carinae faint, with distinct posterodorsal denticles.

***Metasoma.*** Petiole with two low dorsal denticles; postpetiole with four low, short carina, two dorsal and two lateral, lateral pair faint. First gastral tergite with lateral carinae distinct, dorsal carinae absent or faint.

##### *S. radioheadi* queen, male, and larva.

Unknown.

##### *S. radioheadi* geographic range.

Amazonian Venezuela. Map: Figure 31.

##### *S. radioheadi* notes.

The body color of *S. radioheadi* is evenly light yellow, lighter than in other *Sericomyrmex* species. Only dried, pinned specimens were available for this species, however, so the pale color may be due to age. Morphology indicates that *S. radioheadi* is the sister species to *S. bondari*, with which it shares a similar head shape, smooth mandibles, and sharp mesonotal tubercles. *S. bondari* can be separated from *radioheadi* by its larger body size, shorter scape, and shorter and blunter lateral mesonotal tubercles, and by the presence of at least some thick black hairs.

##### *S. radioheadi* etymology.

This species is named after the English rock band Radiohead as an acknowledgement of their longstanding efforts in environmental activism, especially in raising climate-change awareness, and in honor of their music, which is an excellent companion during long hours at the microscope while conducting taxonomic revisions of ants. The species name is a masculine noun in the genitive case.

#### Sericomyrmex saramama sp. n.

Taxon classificationAnimaliaHymenopteraFormicidae

http://zoobank.org/0FE12543-27B8-4E70-B7D5-D3EFC7FA8AEC

**Figure 53.**
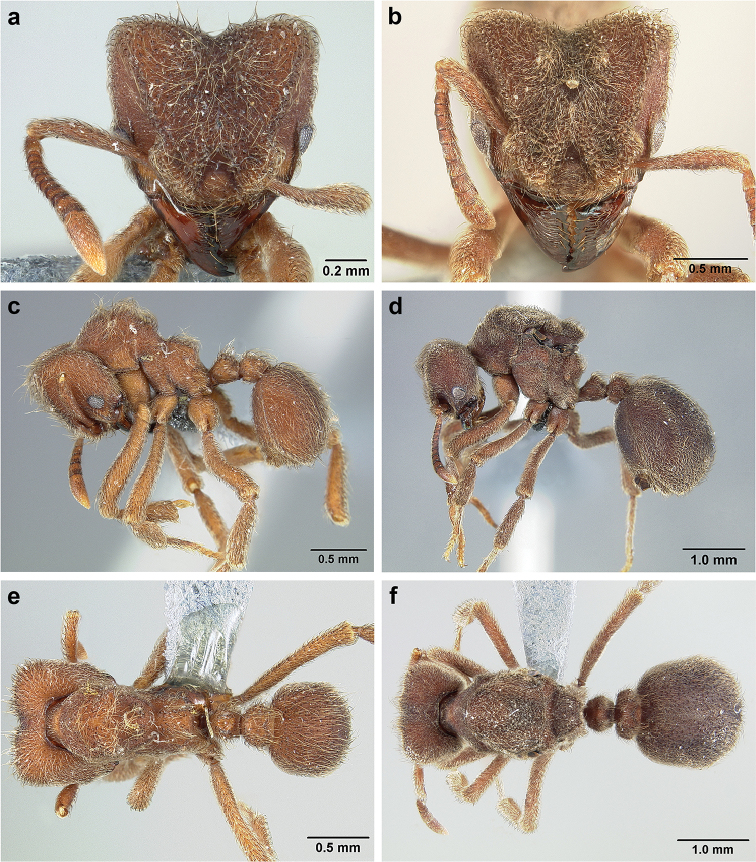
*S. saramama* worker (USNMENT00924064) and queen (USNMENT00924065); head, profile and dorsal views. Worker: **a**, **c**, **e**. Queen: **b**, **d**, **f**.

**Figure 54.**
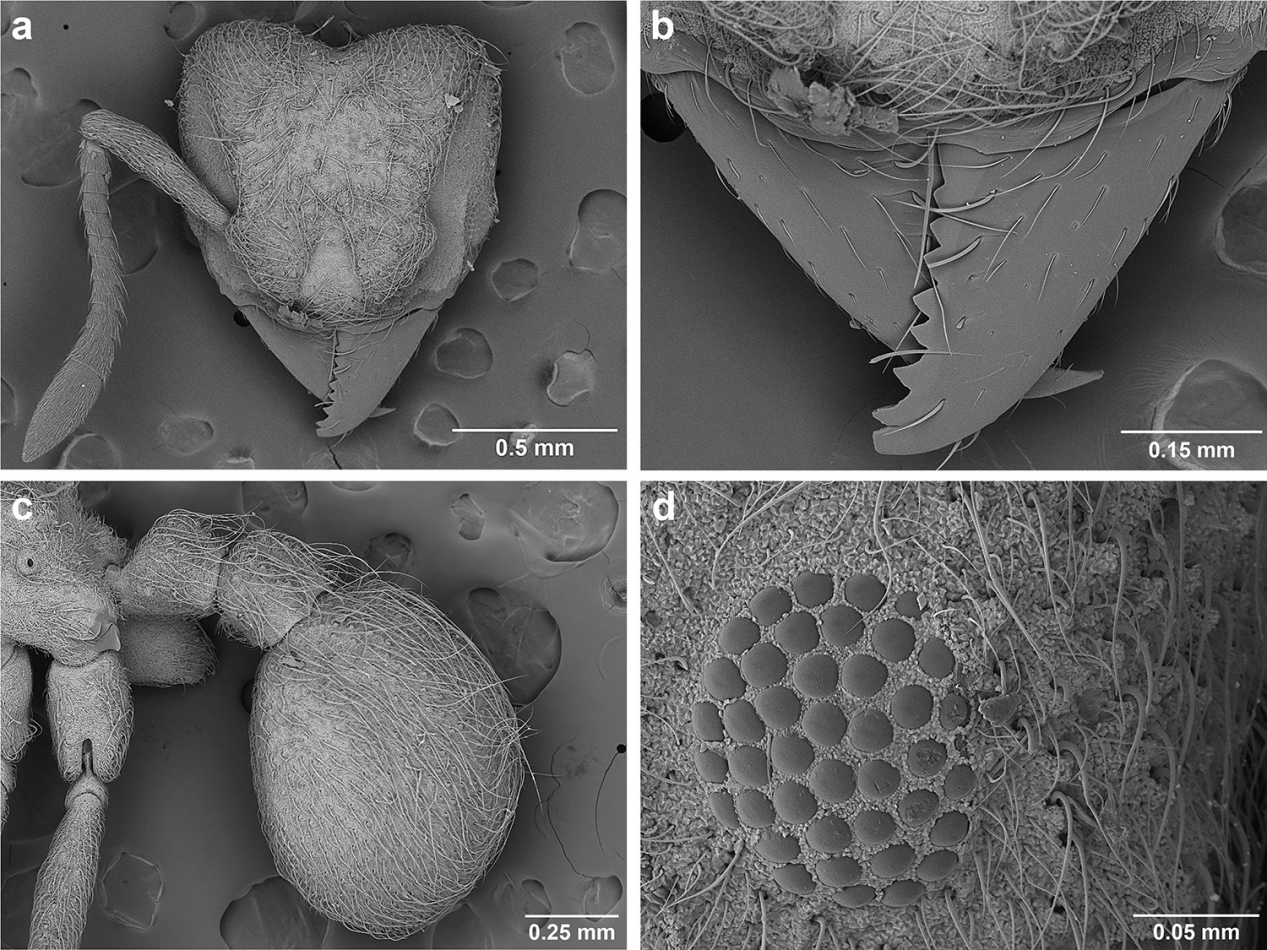
*S. saramama* worker (USNMENT01125262), SEM images. **a** Head, full-face view **b** mandibles **c** metasoma, lateral view **d** eye.

**Figure 55.**
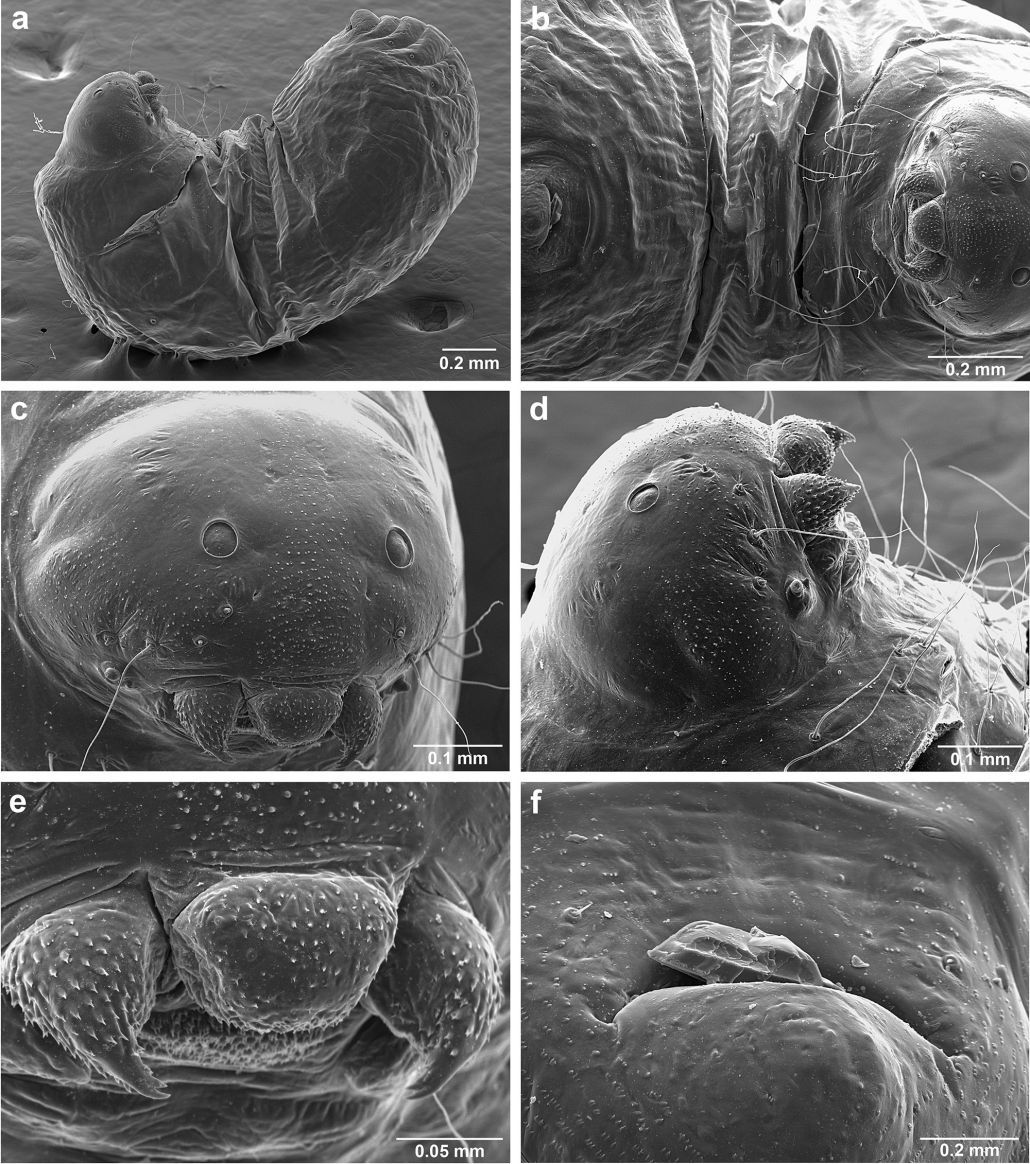
*S. saramama* larva (USNMENT01125266), SEM images. **a** Lateral view **b** ventral view **c** head, frontodorsal view **d** head, lateral view **e** mouthparts **f** anal setae.

Figures 53 , 54 (Worker); Figure 53 (Queen); Figure 55 (Larva); Figure 35 (Map)

##### Type material.

*Holotype worker*: PERU: Madre de Dios, Tambopata Reserve, -12.8187, -69.3636, 224m, A. Ješovnik, AJ120729-03, primary forest, nest on forest trail. (MHNL: 1w, USNMENT00924064). *Paratypes*: same data as holotype (USNM: 1q, USNMENT00924065; 1w, USNMENT00924070) (MZSP: 1w, USNMENT00924068; 1w, USNMENT00924069) (MCZ: 1w, USNMENT00924071; 1w, USNMENT00924080), (CASC: 1w, USNMENT00924072; 1w, USNMENT00924073) (MHNG, 1w, USNMENT00924074) (BMNH: 1w, USNMENT00924077) (MSNG: 1w, USNMENT00924078; 1w, USNMENT00924079).

##### *S. saramama* worker diagnosis.

Small species; mandibles dorsally smooth and glossy; frontal lobe triangular, weakly directed anterad, with short posterior margin; frontal carina complete; eye without white layer; mesosomal tubercles low; gaster with lateral carinae moderately developed, dorsal carinae weakly to strongly developed.

##### *S. saramama* worker description.

Measurements in mm, range (holotype): HWe 0.9–1.13 (1) HW 0.9–1.13 (0.98) HW1 0.6–1.05 (0.93) HW2 0.9–1.16 (1.02) HW3 0.53–0.76 (0.65) IFW1 0.58–0.78 (0.65) IFW2 0.22–0.35 (0.31) HL1 0.84–1.1 (0.96) HL2 0.78–0.98 (0.87) SL 0.65–0.8 (0.72) EL 0.11–0.18 (0.16) Om 7–9 (8) WL 1.12–1.4 (1.3) PL 0.22–0.38 (0.28) PPL 0.19–0.3 (0.25) GL 0.78–0.98 (0.86) HFL 0.95–1.2 (1.13) PW 0.54–0.74 (0.65) CI 97–109 (104) FLI 61–82 (65) SI 68–78 (72) OI 12–17 (16) CEI 7–12 (9) [N=25]

***Pilosity.*** Pubescence dense, lighter than integument, appressed to decumbent. Hairs curved, darker in color at base, appressed to suberect, mostly decumbent.

***Head.*** In full-face view slightly broader than long (CI=103 ± 3), posterior corner smoothly rounded to acute, lateral margin of head slightly convex, posterior cephalic emargination distinct (CEI=10 ± 2), gradually impressed. Vertexal impression usually distinct, frontal tumuli faint. Mandible with 7–8 teeth, dorsally smooth and glossy, finely transversely striate only along masticatory margin. Eye medium-sized (OI =15 ± 1), slightly convex, without white layer, 7–9 ommatidia across largest diameter. Frontal lobe moderately wide (FLI=67 ± 3), triangular, weakly directed anterad, posterior margin shorter than medial. Frontal carina not very robust, usually complete, reaching posterior cephalic corner. Antennal scape moderately long (SI=72 ± 3), sometimes almost reaching posterior cephalic corner.

***Mesosoma.*** Mesosomal tubercles low and obtuse. Propodeal carinae low, rarely with posterodorsal denticles.

***Metasoma.*** Petiole with two low, reduced dorsal denticles; node of postpetiole with two faint, short dorsal carinae, both best seen in dorsolateral view. First gastral tergite with lateral carinae relatively weak, dorsal carinae from barely visible to well developed.

##### *S. saramama* queen description.

Measurements in mm: HWe 1.35 HW 1.4 HW1 1.4 HW2 1.5 HW3 0.95 IFW1 0.93 IFW2 0.38 HL1 1.32 HL2 1.16 SL 0.88 EL 0.22 Om 15 EW 0.11 WL 1.92 PL 0.48 PPL 0.36 GL 1.82 HFL 1.48 PW 1.08 CI 102.27 FLI 68.89 SI 64.81 OI 16 [N=1]

***Head.*** Mandible with 8 teeth, dorsally striate, differing from worker. Preocular carina fading posterior to eye. Eye large, slightly convex, 15 ommatidia across largest diameter. Frontal lobe slightly more robust than in worker.

***Mesosoma.*** Scutum in dorsal view with notauli very reduced, median mesoscutal line absent. Parapsidal lines thin, slightly curved. Scutellum in dorsal view narrowing posteriorly, posterior notch shallow. Propodeal denticles blunt and low, directed posterolaterad.

***Metasoma.*** First gastral tergite with lateral carinae weakly to moderately developed, dorsal carinae absent, anteromedian dorsal groove visible.

##### *S. saramama* male.

Unknown.

##### *S. saramama* larva description.

Setae on dorsal and lateral body surfaces and supra-antennal setae absent. Four genal setae on each side. Mandibular apical tooth divided. Labial denticles absent. First thoracic segment ventrally with multiple multidentate spinules, arranged in transverse rows. Numbers of ventral setae: six on each thoracic segment, two on the abdomen (not including anal setae). Single pair of papilliform setae anterior to anal opening.

##### *S. saramama* geographic range.

Colombia, Ecuador, Peru. Map: Figure 35.

##### *S. saramama* notes.

*S. saramama* can be separated from the similar *S. parvulus* and *S. opacus* by its larger size, complete frontal carinae, larger eyes that lack a white layer, and frontal lobe shape and size. The queen of *S. saramama* is similar in size to *S. opacus* and *S. parvulus* queens, but it can be separated from them by its striate mandibles (smooth in *parvulus* and *opacus*).

Within-species variation includes the dorsal gastral carinae, which are robust in the Peru population but hardly visible in the Ecuador population. *S. saramama* occurs in forested habitats in Peru, Colombia, and Ecuador (Figure 35), but has never been collected in Amazonian Brazil. No other *Sericomyrmex* species has a similar distribution, although the known distribution could easily be an artifact of undersampling. *S. saramama* is the sister species of a clade containing *S. maravalhas* + *scrobifer*, Brazilian cerrado species that are also rarely collected (Figure 35). Together, *S. saramama* + (*S. maravalhas* + *S. scrobifer)* form a clade that is the sister to all remaining *Sericomyrmex* species (Suppl. material 1). Morphologically, *S. saramama* resembles *opacus* more than its sister species. The fungal cultivar associated with this species, at least where it was collected in Peru, is a member of the diverse clade of generalized higher-attine fungi grown by species of *Sericomyrmex* and *Trachymyrmex*, but it occupies a long, separate branch from all other higher-attine cultivars (Ješovnik et al. 2017). *S. saramama* grows this cultivar species even when other cultivars are readily available; it was collected at the same locality in which two *S. mayri* and two *S. parvulus* nests were growing a different fungal species, the “amabilis-mayri” species, that is widely cultivated by *Sericomyrmex* species across the range of the genus.

##### *S. saramama* etymology.

This species is named after the Incan goddess of grain, Saramama, because the type locality is in the former Incan Empire (modern-day Peru) and because *Sericomyrmex* is an ant that practices agriculture. The species name is a noun in apposition.

##### Material examined.

**COLOMBIA**: **Amazonas**: PNN Amacayacu, Matamata, -3.6833, -70.25, 150m, 1 Oct 2001, D. Chota; **ECUADOR: Orellana**: Tiputini, Chorongo trail, -0.6382, -79.8490, 15 Jun 2003, N. M. Gerardo; **PERU: Madre de Dios**: Tambopata Reserve, -12.81867, -69.36364, 224m, 29 Jul 2012, A. Ješovnik.

#### Sericomyrmex saussurei

Taxon classificationAnimaliaHymenopteraFormicidae

Emery, 1894

**Figure 56.**
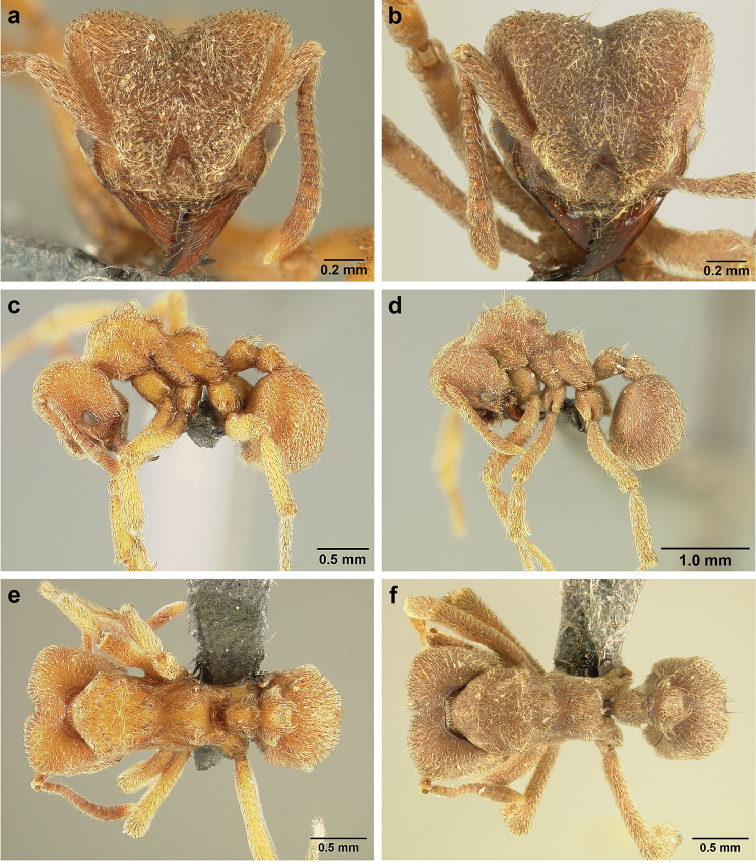
*S. saussurei* worker; head, profile, and dorsal view. Striate-mandibled form (USNMENT01125217) (**a, c, e**). Smooth-mandibled form (USNMENT01125221) (**b, d, f**).

**Figure 57.**
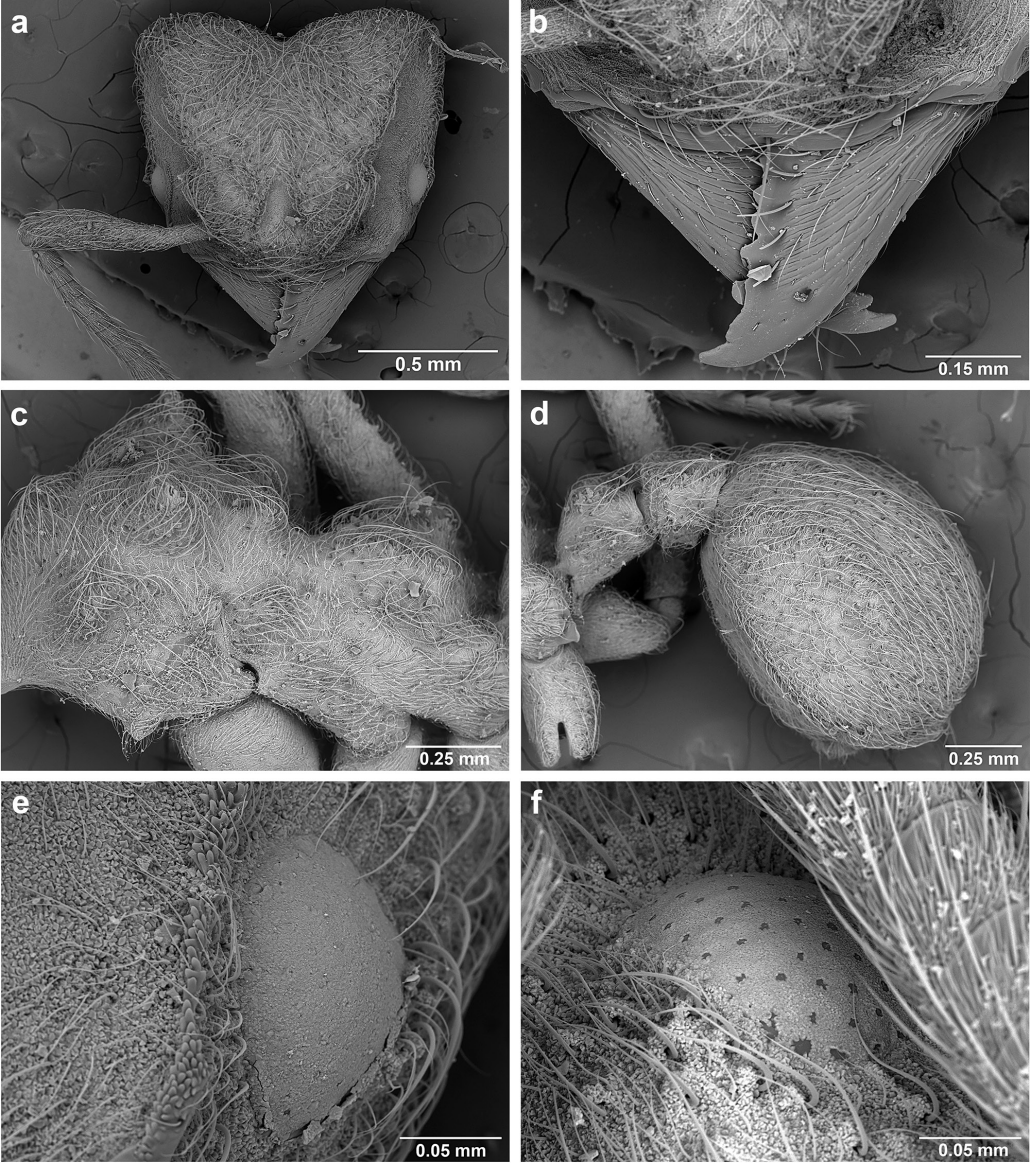
*S. saussurei* worker (USNMENT01126237), SEM images. **a** Head, full-face view **b** mandibles **c** mesosoma, lateral view **d** metasoma, lateral view **e** eyes completely covered with white layer, individual ommatidia not visible **f** eyes with thick white layer, ommatidia visible through small holes in the layer.

**Figure 58.**
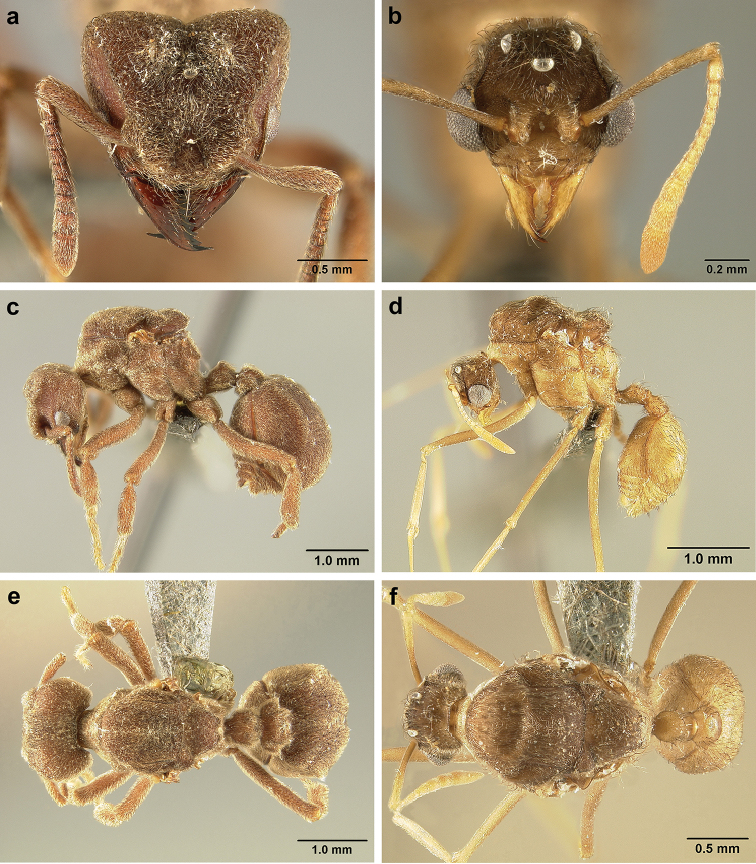
*S. saussurei* queen and male; head, lateral profile, and dorsal view. Queen (USNMENT01125514) (**a, c, e**) Male (USNMENT01125515) (**b, d, f**).

**Figure 59.**
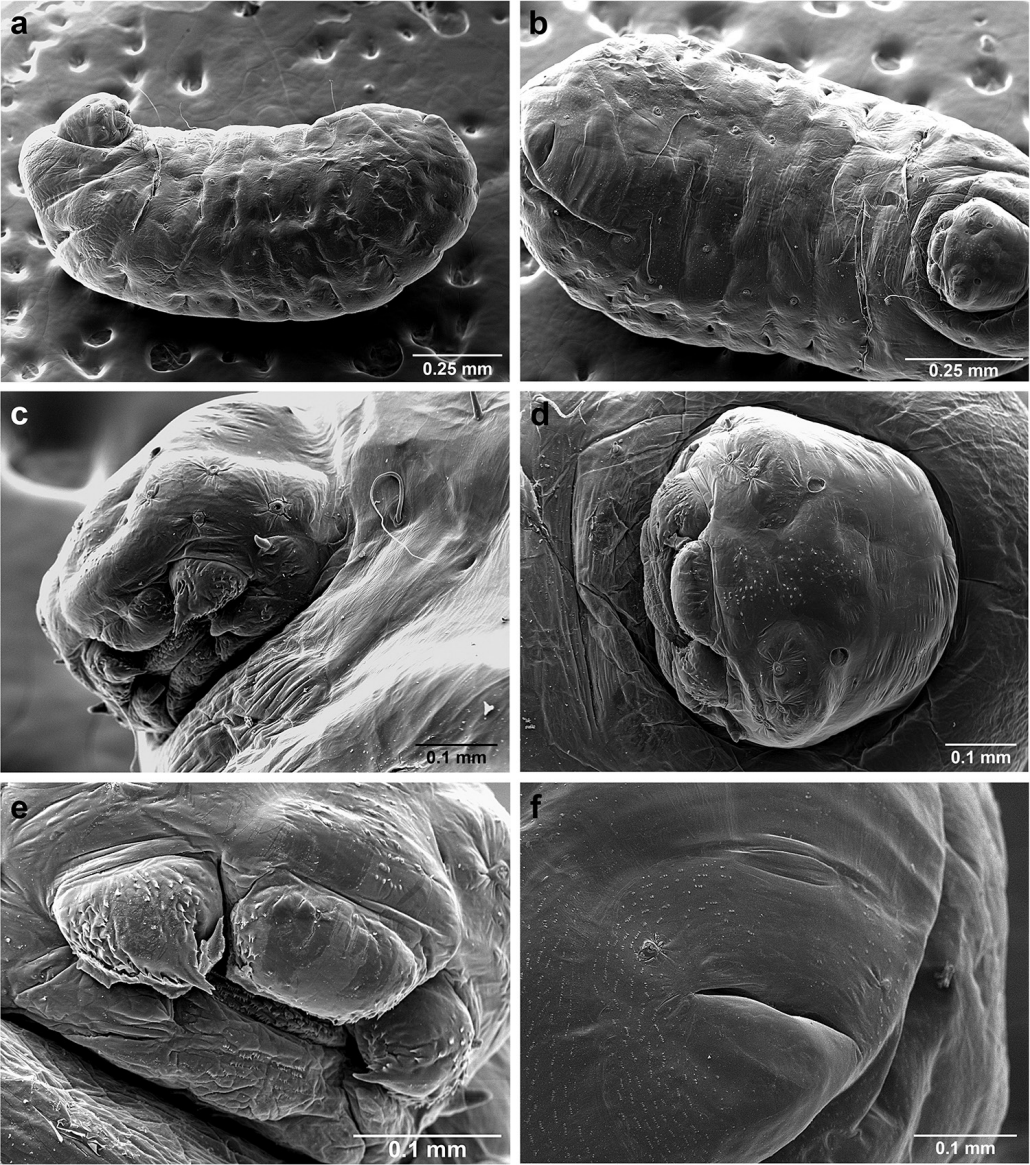
*S. saussurei* larva (USNMENT01126236), SEM images. **a** Lateral view **b** ventral view **c** head, frontolateral view **d** head, dorsal view **e** mouthparts **f** anal setae.

**Figure 60.**
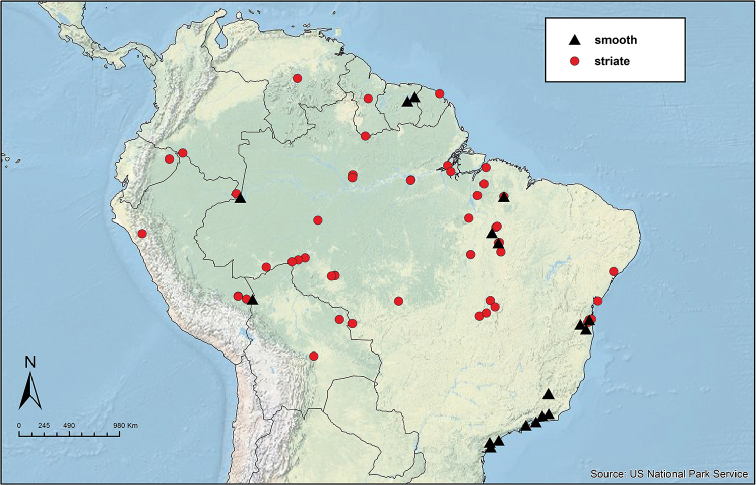
Distribution map of *S. saussurei* indicating smooth-mandibled (black triangles) and striate-mandibled (red circles) forms.

Figures 56 , 57 (Worker); Figure 58 (Queen and male); Figure 59 (Larva); Figures 21 , 60 (Map)

Sericomyrmex saussurei Emery, 1894: 223. *Holotype worker*: BRAZIL, Mato Grosso, [-13, -56], ANTC25804, 1886, P. Germain (MSNG: 1w, USNM00445513).
Sericomyrmex burchelli = Forel, 1905: 183. **syn. n.** Type material examined: BRAZIL, Goiás [-15.9, -50.3], W. J. Burchell (MHNG: 1q, USNM00445563; 1m, USNM00445564).
Sericomyrmex impexus = Wheeler, 1925a: 54. **syn. n.** Type material examined: GUYANA, Cuyuni-Mazaruni, Kartabo [6.3551, -58.6944], Jul-Aug 1920, W. M. M. Wheeler (MCZ: 2w, MCZ-ENT00021138).
S. urichi maracas = Weber, 1937: 395. **syn. n.** Type material examined: TRINIDAD AND TOBAGO, San Juan-Laventille, Maracas Valley [10.75, -61.43], 1 Oct 1935, N. A. Weber, NAW373-1 (MCZ: 2w, MCZ23048).

##### *S. saussurei* worker diagnosis.

Medium-sized species; mandible usually dorsally striate, frontal carina complete; frontal lobe triangular; eye convex, moderately protruding from sides of head, covered with thick white layer; posterior cephalic emargination abruptly to gradually impressed, mesosomal tubercles from low and obtuse to well developed; first gastral tergite with lateral carinae well developed, dorsal carinae weak to well developed.

##### *S. saussurei* worker description.

Measurements in mm, range (holotype): HWe 0.88–1.23 (0.98) HW 0.88–1.23 (NA) HW1 0.82–1.32 (1) HW2 0.92–1.56 (1.13) HW3 0.55–0.84 (0.7) IFW1 0.59–0.88 (0.67) IFW2 0.19–0.36 (0.24) HL1 0.84–1.15 (0.96) HL2 0.76–1.08 (0.87) SL 0.62–0.86 (0.73) EL 0.12–0.24 (0.14) Om 7–11 (NA) WL 1.1–1.64 (1.35) PL 0.21–0.38 (0.25) PPL 0.15–0.3 (0.2) GL 0.70–1.13 (1.13) HFL 0.75–1.38 (1.13) PW 0.61–0.85 (0.76) CI 94–112 (102) FLI 63–74 (69) SI 65–81 (74) OI 12–23 (15) CEI 4–14 (9) [N=68]

***Pilosity.*** Pubescence dense, often lighter than integument, appressed to decumbent. Hairs often curved, darker in color at base, yellow to gray, appressed to suberect, mostly decumbent.

***Head.*** In full-face view slightly broader than long (CI=104 ± 3), posterior corner rounded to angular, posterior cephalic emargination distinct (CEI=9 ± 2), gradually (Figure 7j) to abruptly (Figure 7k) impressed. Vertexal impression and frontal tumuli distinct. Mandible with 7–8 teeth, dorsally glossy and striate, sometimes striation reduced or absent (Figure 56b). Eye medium-sized (OI =15 ± 1), conspicuously convex, protruding slightly from side of head in full-face view, 7–9 ommatidia across largest diameter, always covered with thick, white layer (Figure 6n–o), which makes discerning individual ommatidia difficult. Frontal lobe relatively wide (FLI=69 ± 2), triangular, slightly laterally expanded, with posterior margin shorter than medial. Frontal carina complete, reaching posterior cephalic corner. Antennal scape relatively short (SI=71 ± 2), not reaching posterior cephalic corner.

***Mesosoma.*** Mesosomal tubercles from low and obtuse to moderately pronounced. Propodeal carinae low, reduced, sometimes with posterodorsal denticle.

***Metasoma.*** Petiole and postpetiole with two low, short, serrate, longitudinal carinae dorsally, in petiole sometimes reduced to low denticles, best seen in dorsolateral view. Postpetiole usually with another pair of low carinae laterally. First gastral tergite with lateral carinae well developed, dorsal carinae weak to well developed.

##### *S. saussurei* queen description.

Measurements in mm, range: HWe 1.3–1.38 HW 1.27–1.4 HW1 1.4–1.46 HW2 1.49–1.56 HW3 0.9–0.93 IFW1 0.95–1 IFW2 0.35–0.4 HL1 1.27–1.33 HL2 1.18–1.25 SL 0.9–0.96 EL 0.23–0.29 Om 20–24 EW 0.08–0.1 WL 2–2.16 PL 0.43–0.56 PPL 0.25–0.3 GL 1.83–1.95 HFL 1.22–1.6 PW 1.15–1.2 FWg 7.04–7.37 HWg 4.73–4.73 CI 98–106 FLI 70–75 SI 65–72 OI 17–21 [N=6].

***Head.*** Mandible with 8–9 teeth, dorsally glossy and smooth, finely transversely striate only along masticatory margin. Preocular carina fading posterior to eyes. Eye large (OI=19 ± 2), convex, partially covered with white layer, layer thinner than in workers, 20–24 ommatidia across largest diameter. Frontal lobe as in worker, antennal scape not reaching posterior cephalic corner.

***Mesosoma.*** Scutum in dorsal view notauli faint, median mesoscutal line visible only anteriorly. Parapsidal lines thin, slightly curved. Groove separating axillae in dorsal view weakly transversely costate. Scutellum in dorsal view narrowing posteriorly, posterior margin medially with wide, shallow V-shaped notch. Propodeal carinae low, posteriorly diverging, with posterodorsal denticles.

***Metasoma.*** First gastral tergite with lateral carinae strongly developed, dorsal carinae weak, anteromedian groove visible.

##### *S. saussurei* male description.

Measurements in mm, range: HWe 0.74–0.9 HW 0.62–0.7 IFW1 0.3–0.32 IFW2 0.16–0.19 HL1 0.66–0.68 SL 0.65–0.74 EL 0.25–0.3 Om 23–26 EW 0.13–0.14 WL 1.6–1.72 PL 0.28–0.38 PPL 0.18–0.22 GL 1.18–1.4 HFL 1.52–1.78 PW 0.74–0.88 IOD 0.56–0.61 FWg 4.73–5.23 HWg 3.15–3.4 CI 112–133 FLI 34–41 SI 79–91 OI 32–36 [N=6]

Head longer than broad (CI=125 ± 7), eye large (OI=34 ± 1) and convex, 23–26 ommatidia across the largest diameter. Preocular carina long, extending posteriorly almost to lateral ocellus, slightly curved medially before fading. Notauli and mesoscutal line well developed, integument surrounding parapsidal lines sometimes darker colored, groove between axillae sometimes with one short costa. Propodeum smooth, without protuberances except small spiracular tubercle. Petiole with lateral and dorsal serrate carinae, postpetiole with reduced lateral denticles.

##### *S. saussurei* larva.

Around eight setae on each side of lateral and dorsal body surfaces (i.e., ~16 total). Supra-antennal setae absent. Four genal setae on each side. Mandibular apical tooth divided. Labial denticles present anterior to sericteries. First thoracic segment without multidentate spinules. Numbers of ventral setae: two on T1, two on T2, three on T3, and around 10 on abdomen (not including anal setae). Single pair of setae anterior to anal opening.

##### *S. saussurei* geographic range.

Bolivia, Brazil, Ecuador, French Guiana, Guyana, Peru, Suriname, Trinidad and Tobago, Venezuela. Map: Figure 21, Figure 60.

##### *S. saussurei* notes.

The species most similar to *S. saussurei* is its sister species *S. amabilis*, but *amabilis* can easily be distinguished by its more or less flat eyes lacking the white layer, and usually by geography (Figure 21). *S. mayri*, which, like *S. saussurei* has striate mandibles, is larger, has a wider head, and flat eyes without a white layer. The smaller species *S. parvulus* and *S. opacus* may have a similar white layer over the eyes, but their eyes are small and flat, and the white layer is thinner than in *saussurei*, sometimes incomplete and with individual ommatidia still distinguishable (Figure 6l–m). Also, both *parvulus* and *opacus* have smooth mandibles. The white layer is not as distinct in the queen of *S. saussurei*, but the combination of body size, pilosity, and frontal lobe shape are enough to separate the *S. saussurei* queen from those of the sympatric *mayri*, *bondari*, and *parvulus*.

Several populations of *S. saussurei* have faintly striate or completely smooth mandibles. The atypical smooth-mandibled state was in at least one case consistent within an entire nest; all individuals in a colony collected in Viçosa (Brazil) have smooth mandibles. Individuals with intermediate states are sometimes encountered, with mandibles faintly or partly striate. Smooth-mandibled *saussurei* are distributed more or less across the entire range of the species, but are more concentrated in eastern Brazil (Figure 60). However, in the molecular phylogeny the two forms cluster into two imperfect subclades in which some striate-mandibled forms mix with predominantly smooth-mandibled forms and vice versa, and a similar pattern is observed in principal component analyses of the morphological measurement data (Figure 5h). More data are clearly needed to determine if the smooth-mandibled variant represents a separate species. Here we choose to include both forms in a single species because they are morphologically very similar, the molecular evidence is indecisive, and this character is generally plastic (e.g., both *amabilis* and *mayri*, which typically have striate mandibles, likewise have a few populations with smooth or smoother mandibles).

Interestingly, the white layer on the eyes of *saussurei* is exceptionally consistent compared to character-state distributions in other *Sericomyrmex* species. In fact, it is among the most consistent of all morphological characters across all *Sericomyrmex* species. In all specimens of *S. saussurei* examined, from across a large geographic range (Figure 21), the eyes are convex and covered with a thick white layer. Similar white layers are also seen in *parvulus* and *opacus*. In those species, however, the layer itself is thinner and often incomplete, and the eyes are generally smaller and flat, creating a distinctly different appearance (Figure 6l–m). Also, in both *parvulus* and *opacus* the layer is completely absent in some individuals or populations. In the remaining species of *Sericomyrmex* the eyes are uncoated, without a white layer. It would be interesting to determine the biological significance of this layer and to analyze its chemical properties. Based on our SEM images it seems to be an extension of the waxy, crystal-like cuticular layer found on the integuments of workers and queens in all *Sericomyrmex* species (Figure 6b–c), but which is absent in males and in callow workers (Figure 6d–f), as well as in some individuals of *S. maravalhas*. Why this layer extends to and completely covers the eyes in some species but not others remains unknown.

*Synonymies*. No character states in *S. impexus* and *S. urichi maracas* distinguish them from *S. saussurei*. The two examined syntypes of *impexus* are only slightly larger in size than the single *saussurei* type specimen, but well within the size range for the species. When describing *impexus* Wheeler (1925a) only distinguishes it from *urichi* and *lutzi*. Likewise, in his description of *urichimaracas* Weber (1937) simply lists differences from *urichi*, to which this species is not very closely related (*S. urichi* is a junior synonym of *S. mayri*). *S. burchelli*, described only from a queen and a male, can be recognized as belonging to *saussurei* based on morphological measurements, and in the queen also by a thin white layer partially covering the eye. Forel (1905) separates the queen of *burchelli* from *saussurei* by its sparser hair, more developed petiole and postpetiole, and less developed metanotal tubercles. However, this comparison must have been made to asaussurei worker, because the queen of *S. saussurei* was unknown prior to the present study. Consequently, Forel’s (1905) differences are due both to differences between the worker and queen castes as well as to observed within-species variation.

##### Material examined.

**BOLIVIA: Santa Cruz**: 10 km NW Terevinto, -17.6667, -63.45, 380m, 9 Dec 1993, P. S. Ward; Aserradero Moira, -14.5667, -61.2, 180m, 27 Nov 1993, P. S. Ward; **BRAZIL: Amazonas**: Floresta de Tapauá, km 4, -6.01, -63.1, 69m, 13 Oct 2013, I. O. Fernandes; Manaus, Br 174, Km.70, [-2.26, -60.04], 95m, 30 Aug 1995, H. Vasconcelos; Pres. Figueredo, I. Pe Inchado, [-2.02, -60.02], 26 Aug 1994, Queiroz; **Bahia**: CEPLAC, arboreto, -14.7535, -39.2313, 11 Oct 2013, J. H. C. Delabie; Ilhéus, Ponta do Ramo, Cacau 02, -14.5294, -39.0619, 28 Aug 1998, J. R. Maia; Itabuna, Ferradas A27, -14.8258, -39.4044, 21 Sep 2000, J. R. M. Santos; Itororó, -14.9744, -40.0502, 11 Aug 2000, J. R. M. Santos; Maraú, Fazenda Água Boa, -14.5847, -39.2672, 1 Jul 1997, J. R. M. Santos; Salvador, [-12.9833, -38.5167], 1 Oct 2012, T. S. Melo; Uruçuca, -14.5847, -39.2672, 16 Dec 1997, J. R. M. Santos; **Goiás**: Cavalcante, Serra da Contenda, -13.4951, -47.5504, 15 Oct 2004, R. R. Silva, B. H. Dietz; Niquelândia, -14.0166, -48.3, 24 Sep 1995, R. Silvestre, B. H. Dietz, C. R. F. Brandão; **Maranhão**: Bom Jardim, REBIO Gurupi Parcela 01 A2, -3.9258, -46.7712, 19 Sep 2014, A. Y. Harada; Estreito, Fazenda Itaueiras, -6.5317, -47.3711, 1 Jun 2005, R. R. Silva, R. M. Feitosa; Estreito, João Lisboa, -6.5317, -47.3711, 1 Jun 2005, R. R. Silva, R. M. Feitosa; **Minas Gerais**: Camacan, Serra Bonita, -15.3907, -39.5634 ±6m, 789m, 20 Mar 2009, J. Sosa-Calvo; Serra de Ricardo Franco State Park, -14.9076, -60.0646, 200m, 7 Feb 2014, J. Maravalhas; Viçosa, Mata do Seu Nico, -20.7833, -42.8333, 8 May 2013, R. Jesus, J. Chaul; Viçosa, UFV Mata da Biologia, -20.7578, -42.8636, 10 Oct 2015, J. Chaul, S. Epifânio; **Pará**: Alter do Chão, -2.4607, -54.926 ±6m, 39m, 27 Jan 2009, J. Sosa-Calvo; Goianésia, Faz. Rio Capim, [-3.8384, -49.0986], 16 Jun 2003, A. M. Elizabeth; Gurupá, [-1.197, -51.7], 18 Oct 2003, J. M. S. Vilhena; Marituba, Cacau, [-1.3666, -48.3333], 16 Oct 2004, J. R. M. Santos; Melgaço, Caxiuanã, ECFPn IV Transecto 9-100) Winkler #5, -1.7248, -51.4230, 27 Jun 2003, A. Y. Harada; Paranapuebas Palmares, Lote BPR Mensa Ponto: 35583, -5.8072, -49.8325, 9 Apr 2008, M. Martíns; Tailândia, Faz. Marupiara, Parcela 05, -2.8121, -48.5122, 25 Apr 2013, M. Tavares, A. Palmeira; **Rio de Janeiro**: Nova Iguaçu, ReBio Tinguá, -22.5705, -43.4141, 2 Feb 2002, A. Mayhe, S. Veiga-Ferreira; Restinga da Marambaia, [-23.0685, -43.9531], P. S. Meneguete; Teresopólis, PN Serra dos Órgãos, -6.5317, -47.3711, 23 Nov 1999, Racha, B. H. Dietz, Rosa; **Rondônia**: Jaci-Paraná, km 0, subparcela 100, -9.2656, -64.2125, 94m, 27 Jan 2013, I. O. Fernandes; Ji- Paraná, -10.7997, -61.5947, 273m, 7 Oct 2008, T. R. Schultz; **São Paulo**: Cananéia, Ilha do Cardoso, -28.0968, -47.9298, 24 Dec 2002, R. R. Silva, C. R. F. Brandão, C. Scott; Jacupiranga, [-24.7055, -48.0167], 1 Nov 1963, F. Plaumann, C. Kempf; Jureia-Itantins, -24.54416, -47.235; Picinguaba, P.E. Serra do Mar, -23.3361, -44.8375, 1 Apr 2001, C. R. F. Brandão; **Tocantins**: Aguiarnopólis, -6.6137, -47.4814, 14 Jan 2005, R. R. Silva, R. Silvestre; Araguacema, Rio Tiririca, -8.9886, -49.6675, 16 Nov 2005, R. R. Silva, R. M. Feitosa; Babaçulândia, -7.0878, -47.8286, 10 Dec 2001, R. R. Silva, N. L. Albuquerque; Goiatins, -7.9413, -47.1586, 3 May 2005, R. R. Silva, B. H. Dietz; Paraná, -12.9343, -47.9618, 13 Oct 2004, R. R. Silva, B. H. Dietz; Recursolândia, -8.7579, -47.0388, 9 May 2005, R. R. Silva, B. H. Dietz; **COLOMBIA: Amazonas**: Leticia, Reserva Forestal Del Río Calderón, Estac. Biol. El Zafire, -4.0058, -69.9125, 146m, 1 Dec 2007, L.E. Franco, S. Florez; PNN Amacayacu Matamata, -3.6833, -70.25, 150m, 15 Oct 2000, A. Parente; **Putumayo**: PNN La Paya Cabaña Viviano Cocha, -0.1166, -74.9666, 350m, 1 Jun 2003, R. Cobete; **ECUADOR: Orellana**: Tiputini Biodiversity Station, -0.6333, -76.1333, 220m, 14 Feb 2002, K. T. Ryder Wilkie; **FRENCH GUIANA: Cayenne**: Kourou, 5.096, -52.404, 18 Dec 2014, M. Fichaux, Orivel; **GUYANA: Potaro-Siparuni**: Iwokrama, Kurapakari base camp, [4.6698, -58.6854], 60m, 9 Apr 1996, T. R. Schultz, U. G. Mueller; **Upper Takutu-Upper Essequibo**: Acari Mountains, nr. Romeo’s camp, 1.3833, -58.9333, 282m, 10 Oct 2006, J. Sosa-Calvo; **PERU: Cajamarca**: Cajamarca, 32 km W Jaen, -7.1667, -78.5167, 600m, 19 Jan 1955, E. I. Schlinger, E. S. Ross; **Madre de Dios**: PN Pampas de Heath, Río Heath, -12.8333, -68.8333, 26 Jul 1991, B. L. Fisher; Puerto Maldonado, Los Amigos Biol. Station, trail 3, Huangana, -12.569, -70.1008, 277m, 9 Oct 2004, T. R. Schultz, J. Sosa-Calvo; Tambopata Reserve, -12.8187, -69.3636, 224m, 1 Aug 2012, A. Ješovnik; **SURINAME: Sipaliwini**: Lely Mountains, 4.4507, -55.2302, 550 m, 29 Oct 2005, J. Sosa-Calvo, R. Badal; Nassau Mountains, 4.8172, -54.6067, 514m, 3 Nov 2005, J. Sosa-Calvo; **VENEZUELA: Bolívar**: Río Tawadu, Nichare Field St., 6.4333, -64.8833, 200m, 9 Feb 1966, D. M. Olson.

#### Sericomyrmex scrobifer

Taxon classificationAnimaliaHymenopteraFormicidae

Forel, 1911

**Figure 61.**
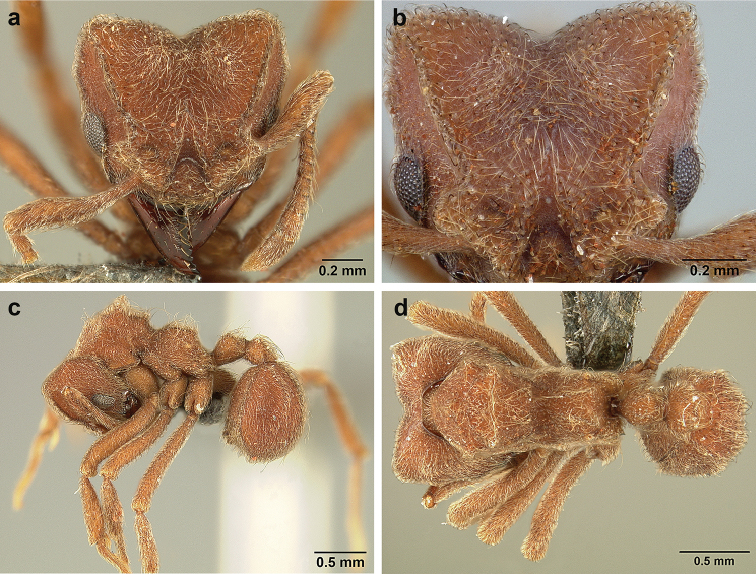
*S. scrobifer* worker (USNMENT01125115). **a** Head **b** head (detail) (USNMENT01125290) **c** lateral profile; and **d** dorsal view.

**Figure 62.**
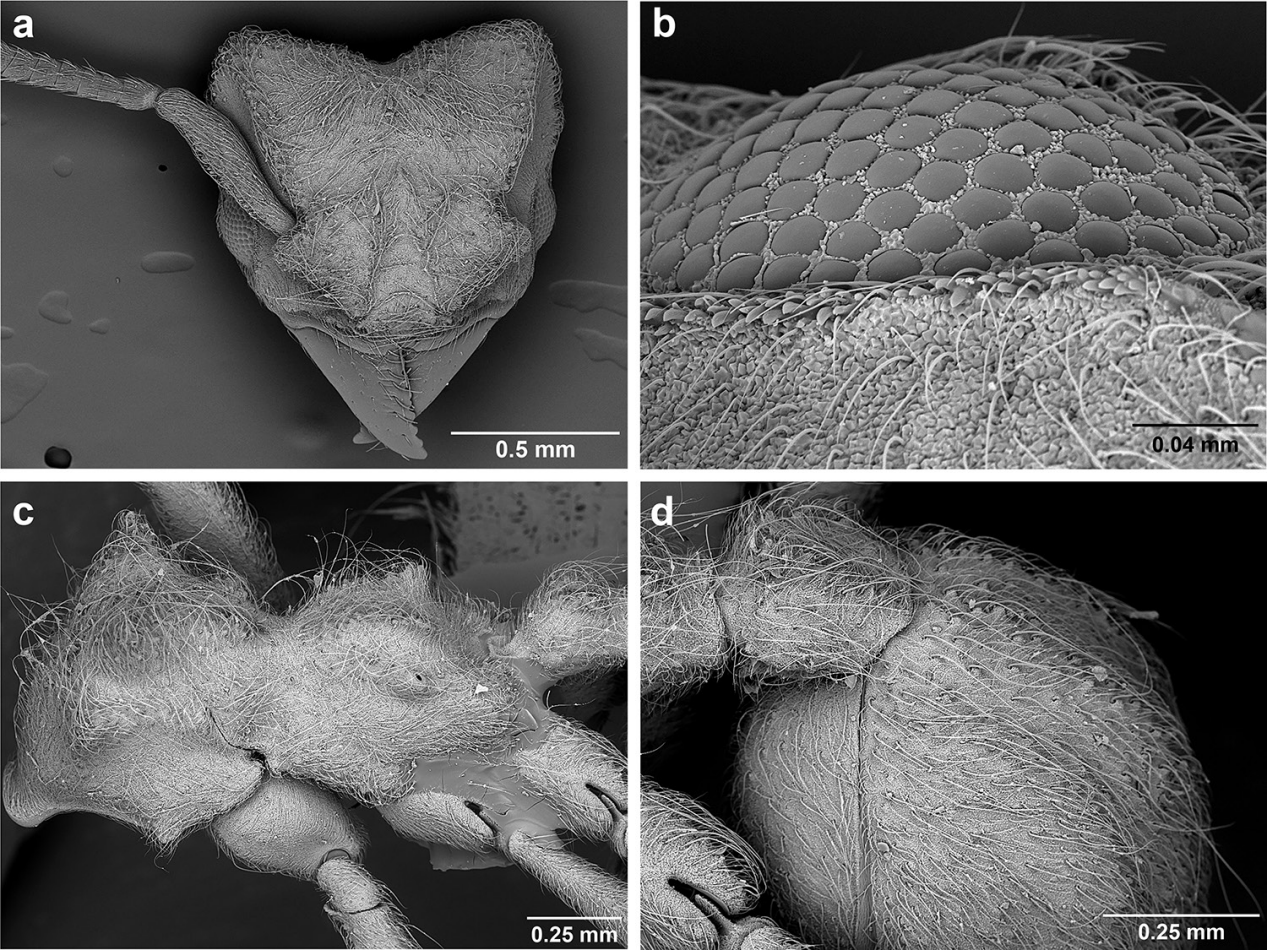
*S. scrobifer* worker (USNMENT01125273), SEM images. **a** Head, full-face view **b** eye **c** mesosoma, lateral view **d** metasoma (partial), dorsolateral view.

Figures 61 , 62 (Worker); Figure 35 (Map)

Sericomyrmex scrobifer Forel, 1911: 296. Lectotype worker (here designated): BRAZIL, São Paulo,Ypiranga, [-23.5, -46.6], ANTC35980, Luederwaldt (NHMB, 1w, CASENT0912518). *Paralectotypes*: same data as lectotype (MHNG: 3w, USNMENT00445578).

##### *S. scrobifer* worker diagnosis.

Medium-sized species; mandible dorsally smooth and glossy; frontal lobe robust, wide, trapeziform to rectangular; frontal carina strongly developed; eye large, convex, protruding laterally in full-face view, lateral mesonotal tubercles sharp, first gastral tergite with both lateral and dorsal carinae strongly developed.

##### *S. scrobifer* worker description.

Measurements in mm, range (lectotype): HWe 0.83–1.12 (1) HW 0.78–1.05 (NA) HW1 0.83–1.13 (1.03) HW2 0.88–1.2 (1.1) HW3 0.55–0.85 (0.7) IFW1 0.66–0.84 (0.78) IFW2 0.22–0.32 (0.22) HL1 0.8–1.1 (1) HL2 0.7–0.98 (0.9) SL 0.62–0.78 (0.68) EL 0.15–0.24 (0.17) Om 10–14 (13) WL 1.12–1.4 (1.25) PL 0.24–0.38 (0.24) PPL 0.2–0.28 (0.25) GL 0.74–1.68 (0.95) HFL 0.93–1.26 (1.07) PW 0.63–0.8 (0.7) CI 97–111 (100) FLI 72–83 (78) SI 62–77 (68) OI 16–23 (17) CEI 8–15 (10) [N=31]

***Pilosity.*** Pubescence dense, often lighter than integument, appressed to decumbent. Setae often curved, darker in color at base, appressed to suberect, mostly decumbent.

***Head.*** In full-face view slightly broader than long (CI=104 ± 3), posterior corner angular to acute. Lateral margin of head straight to slightly convex, posterior cephalic emargination distinct, relatively deep (CEI=11 ± 1), gradually impressed. Vertexal impression usually distinct, frontal tumuli faint. Mandible with 7–8 teeth, dorsally smooth and glossy, finely transversely striate only along masticatory margin. Eye large (OI=20 ± 2), distinctly convex, protruding from sides of head in full-face view, without white layer, 10–14 ommatidia across largest diameter. Frontal lobe wide (FLI=76 ± 3), laterally expanded, trapeziform to rectangular, posterior margin as long as medial, slightly shorter in some specimens, lateral margin sometimes mildly concave and serrate (Figure 61b). Frontal carina robust, complete, straight to slightly curved laterally. Antennal scape relatively short, not reaching posterior cephalic corner (SI=70 ± 3).

***Mesosoma.*** Lateral mesonotal tubercles well developed, acute, sometimes weakly tuberculate apically. Propodeal carinae low, sometimes serrate, with low posterodorsal denticles.

***Metasoma.*** Petiole with two low, reduced dorsal denticles, node of postpetiole with two faint, short dorsal carinae, and two low lateral carinae, best seen in dorsolateral view. Postpetiole in dorsal view sometimes slightly posteriorly emarginate. First gastral tergite with lateral and dorsal carinae strongly developed.

##### *S. scrobifer* queen, male, and larva.

Unknown.

##### *S. scrobifer* geographic range.

Brazil, Paraguay. Map: Figure 35.

##### *S. scrobifer* notes.

*S. scrobifer* is most similar to its sister species, *S. maravalhas*, from which *scrobifer* can be separated by its larger size; much wider, trapeziform frontal lobes; larger, more protruding eyes; and stronger frontal carinae. The combination of large eyes, trapeziform frontal lobes, and four carinae on the gaster will separate it from all other *Sericomyrmex* species. Smaller individuals can have less pronounced mesonotal tubercles and weaker dorsal and lateral gastral carinae.

##### *S. scrobifer* material examined.

**BRAZIL: Bahia**: Vitória da Conquista, -14.84, -40.84, 27 Jan 1997, J. H. C. Delabie; **Goiás**: Faz. Cachoeirinha, Jataí, [-17.89, -51.70], 28 Oct 1962, Exp. Dep. Zool.; **Mato Grosso**: Utiariti, Rio Papagaio, [-13.03, -58.28], 23 Oct 1966, Lenko, Pereira; **Minas Gerais**: Santana do Riacho, -19.17, -43.72, 19 Feb 2001, S. M. Soares; Uberlândia, Clube Caça, Pesca, [-18.97, -48.28], 14 Sep 2007, R. M. Feitosa; Uberlândia, Panga, -19.1667, -48.3833, 816m, 2 Sep 2008, T. R. Schultz; **Paraná**: Jaguariaíva, -24.168, -49.667, 804m, 15 Jan 2015; **Piaui**: Rio Uruçuí-Preto, [-7.3431, -44.6168], 20 Feb 1976, R. Negrett; **São Paulo**: São Paulo, [-21.8, -48.5], 26 May 1905, N. A. Weber; Agudos, [-22.46, -48.97], 27 Apr 1952, W. W. Kempf; Faz. Itaquerê, Boa Esperança do Sul, [-21.9802, -48.3881], 25 Jan 1964, K. Lenko; Mogi Guaçu, -22.37, -46.94, 570m, 6 Feb 1997, I. R. Leal; **Tocantins**: Cartucho e Goiatins, [-8.10, -47.64], 9 Nov 1998, C. R. F. Brandão, C. I. Yamamoto. **PARAGUAY**: Canindeyú: Reserva Mbaracayú, Aguara Ñu, -24.1833, -55.2833, 240m, 16 Nov 2002, A. L. Wild.

## Supplementary Material

XML Treatment for Sericomyrmex amabilis

XML Treatment for Sericomyrmex bondari

XML Treatment for Sericomyrmex lutzi

XML Treatment for Sericomyrmex maravalhas

XML Treatment for Sericomyrmex mayri

XML Treatment for Sericomyrmex opacus

XML Treatment for Sericomyrmex parvulus

XML Treatment for Sericomyrmex radioheadi

XML Treatment for Sericomyrmex saramama

XML Treatment for Sericomyrmex saussurei

XML Treatment for Sericomyrmex scrobifer

## Supplementary materials

Supplementary material 1 Data type: Adobe PDF file
**UCE phylogeny**
Explanation note: The maximum-likelihood phylogeny of the 90% complete concatenated matrix containing 799 UCE loci (702,574 base pairs), adapted from Ješovnik et al. 2017. This dataset is made available under the Open Database License (http://opendatacommons.org/licenses/odbl/1.0/). The Open Database License (ODbL) is a license agreement intended to allow users to freely share, modify, and use this Dataset while maintaining this same freedom for others, provided that the original source and author(s) are credited.Ana Ješovnik, Ted R. Schultz

Supplementary material 2 Table S2. Full list of measured, imaged, and type specimens; localities for all specimens examined; and full statistics for morphological measurements. Data type: specimens measurements Explanation note:
**a) Measured specimens.** All workers measured for this study, with all measurements in millimeters.
**b) Measured specimens.** All queens and males measured for this study, with all measurements in millimeters.
**c) Imaged specimens**. Specimen data for all figures.
**d) Type specimens**. Specimen data for type specimens examined in this study.
**e) Statistics** for all measurements and indices for each species.
**f) Localities list**. A list of localities and other specimen data for all pinned specimens examined.
**g) *S. mayri* populations.** A list of localities and other specimen data for specimens used to create the *S. mayri* population map (Figure 42). This map is based on the subset of *S. mayri* samples for which molecular data (either UCE or COI) were obtained. This dataset is made available under the Open Database License (http://opendatacommons.org/licenses/odbl/1.0/). The Open Database License (ODbL) is a license agreement intended to allow users to freely share, modify, and use this Dataset while maintaining this same freedom for others, provided that the original source and author(s) are credited.Ana Ješovnik, Ted R. Schultz
