# Corrigenda: Ješovnik A, Schultz TR (2017) Revision of the fungus-farming ant genus *Sericomyrmex* Mayr (Hymenoptera, Formicidae, Myrmicinae). ZooKeys 670: 1–109. https://doi.org/10.3897/zookeys.670.11839

**DOI:** 10.3897/zookeys.691.15088

**Published:** 2017-08-17

**Authors:** Ana Ješovnik, Ted R. Schultz

**Affiliations:** 1 Department of Entomology, National Museum of Natural History, Smithsonian Institution, Washington, District of Columbia, United States of America; 2 Maryland Center for Systematic Entomology, Department of Entomology, University of Maryland, College Park, Maryland, United States of America

In our recent revision (Ješovnik and Schultz 2017) of the ant genus *Sericomyrmex*, we failed to address the taxonomic standing of the subspecies *S. opacus muelleri* Forel, 1912. Even though we examined the type specimen, measured it, and included it in Suppl. material 1: Table S2, we did not include it in our synonymy list because *S. opacus muelleri* was described as a variety (Forel 1912) and not as a subspecies. However, it has subsequently been brought to our attention that, according to International Code of Zoological Nomenclature Articles 45.5 and 45.6 (International Commission on Zoological Nomenclature 1999), if a variety was described before 1961 it should be treated as a subspecies. We therefore here correct the relevant portions of the abstract, taxonomic synopsis for the genus, detailed taxonomic synopsis for the species, and notes section of *S. mayri*, as well as Suppl. material 1: Table S2.

Corrected “Abstract” (p. 1):

The following species and subspecies are synonymized: under *S. opacus* [=*S. aztecus* Forel **syn. n.**, *S. zacapanus* Wheeler **syn. n.**, and *S. diego* Forel **syn. n.**]; under *S. bondari* [=S. *beniensis* Weber **syn. n.**]; under *S. mayri* [=*S. opacus muelleri* Forel **syn. n.**, =*S. luederwaldti* Santschi **syn. n.**, *S. moreirai* Santschi **syn. n.**, *S. harekulli* Weber **syn. n.**, *S. harekulli arawakensis* Weber **syn. n.**, *S. urichi* Forel **syn. n.**]; under *S. saussurei* [=*S. burchelli* Forel **syn. n.**, *S. impexus* Wheeler **syn. n.**, *S. urichi maracas* Weber **syn. n.**]; and under *S. parvulus* [=*S. myersi* Weber **syn. n.**].

Corrected “**Taxonomic synopsis**” for the genus (p. 31):

***Sericomyrmex mayri*** Forel, 1912, Colombia to Bolivia and Brazil (w, q, m, l).

=*Sericomyrmex opacus muelleri* Forel, 1912, **syn. n.**

=*Sericomyrmex urichi* Forel, 1912, **syn. n.**

=*Sericomyrmex luederwaldti* Santschi, 1925, **syn. n.**

=*Sericomyrmex moreirai* Santschi, 1925, **syn. n.**

=*Sericomyrmex harekulli* Weber, 1937, **syn. n.**

=*Sericomyrmex harekulli arawakensis* Weber, 1937, **syn. n.**

Corrected detailed taxonomic synopsis for the species (pp. 61–62):

***Sericomyrmex mayri* Forel, 1912**

Figures 36, 37, 38 (Worker); Figure 39 (Queen and male); Figure 40 (Larva); Figure 41 (Map)

*Sericomyrmex mayri* Forel, 1912: 194. *Lectotype worker* (here designated): BRAZIL, Rio de Janeiro, Niterói, [-22.8751, -43.2775], ANTC31816, A. Forel, (MHNG: 1w, CASENT0909370). *Paralectotypes*: same data as lectotype (MHNG: 1w, US- NMENT00445567; 3m, USNMENT00445580).

=*Sericomyrmex opacus muelleri* Forel, 1912, **syn. n.** Type material examined: BRAZIL, ANTC31817, A. Forel, (MHNG: 1q, CASENT0909371).

=*Sericomyrmex urichi* Forel, 1912: 193. **syn. n.** Type material examined: TRINIDAD AND TOBAGO, ANTC31818, F. W. Urich (MHNG: 3w, CASENT0909372).

=*Sericomyrmex luederwaldti* Santschi, 1925: 15. **syn. n.** Type material examined: BRAZIL, Minas Gerais, Pirapora, [-17.355, -44.9447], ANTC35978, ANTC25817, E. Garbe (NHMB: 5w, CASENT0912516) (MSNG: 1w, CASENT0904989).

=*Sericomyrmex moreirai* Santschi, 1925: 16. **syn. n.** Type material examined: BRAZIL, Rio de Janeiro, [-22.8751, -43.2775], ANTC35979, Moreira (MCZ: 2w, MCZ 1-2 21140) (NHMB: 3w, CASENT0912517; 2w, USNMENT01126231; 2q, USNMENT01126232).

=*Sericomyrmex harekulli* Weber, 1937: 398. **syn. n.** Type material examined: GUYANA, East Berbice-Corentyne, Oronoque River, [2.75, -57.4167], NAW598, 27 Jul 1936, N. A. Weber (USNM: 1w, USNMENT00529483) (MCZ: 2w, USNMENT00924104; 2w, USN- MENT00924105)

=*Sericomyrmex harekulli arawakensis* Weber, 1937: 399. **syn. n.** Type material examined: GUYANA, Cuyuni-Mazaruni, Mazaruni River, Forest Settlement, [6.39733, -58.6781], 10 m, NAW 277, 15 Aug 1935, N. A. Weber (MCZ: 2w, MCZ 23051; 2w, 1q, USNMENT00924106)

Corrected “**Synonymy**” section in the “*S. mayri* notes” section (pp. 68–69):

The examined syntypes of *S. luederwaldti*, *S. harekulli*, and *S. harekulli arawakensis* conform to typical *S. mayri* morphology. Their original authors (Forel 1912, Santschi 1925, Weber 1937) focus on slight differences in mesosomal tubercles, head shape, and scape length, all of which are variable within *mayri*. Likewise, the subspecies *S. opacus muelleri*, described from a single queen specimen by Forel, is a typical *mayri* queen, both in morphological characters and measurements. The *moreirai* syntypes have the cephalic emargination less pronounced than in the *mayri* lectotype, but this difference is encompassed by the range of variation in *mayri* as here defined. In his description of *S. moreirai*, Santschi (1925) calls it the “neighbor” of *mayri*, but says it is “much more stocky.” He also compares *moreirai* with *urichi* and reports small differences in pilosity and mesosomal tubercles, both of which fall within the variation observed in *S. mayri*. The syntypes of *urichi* we examined, unlike the *mayri* lectotype, have almost completely smooth mandibles, but, as discussed above, smooth mandibles are encountered in some *mayri* populations, especially those from Trinidad and Tobago, the type locality of *urichi.* In all other characters and measurements, *urichi* clearly agrees with *S. mayri*. In his description Forel (1912) distinguished *mayri* and *urichi* by complete versus incomplete frontal carinae and by the depth of the cephalic emargination, but he does not mention striate vs. smooth mandibles. Again, the cited differences (depth of the emargination, length of the frontal carinae, and degree of mandibular sculpture) fall within the range of observed intraspecific variation in *S. mayri* as here defined.

**Table:** Corrected Suppl. material 2: Table S2 (sheet **d** Type specimens):

Genus	Species	Type	Pins	Institution	Specimen code	Coll. code	Collector	Country
*Sericomyrmex*	*mayri*	*opacusmuelleri* holotype	1pin, 1q	MHNG	CASENT0909371	ANTC31817	A. Forel	Brazil

## Supplementary materials

Supplementary material 1 Table S2. Full list of measured, imaged, and type specimens; localities for all specimens examined; and full statistics for morphological measurements. Data type: specimens measurements Explanation note:
**a) Measured specimens.** All workers measured for this study, with all measurements in millimeters.
**b) Measured specimens.** All queens and males measured for this study, with all measurements in millimeters.
**c) Imaged specimens**. Specimen data for all figures.
**d) Type specimens**. Specimen data for type specimens examined in this study.
**e) Statistics** for all measurements and indices for each species.
**f) Localities list**. A list of localities and other specimen data for all pinned specimens examined.
**g) *S. mayri* populations.** A list of localities and other specimen data for specimens used to create the *S. mayri* population map (Figure 42). This map is based on the subset of *S. mayri* samples for which molecular data (either UCE or COI) were obtained. This is an open access article distributed under the terms of the Creative Commons Attribution License (CC BY 4.0), which permits unrestricted use, distribution, and reproduction in any medium, provided the original author and source are credited.Ana Ješovnik, Ted R. Schultz

