# Supplementary material for: Revision of the fungus-farming ant genus Sericomyrmex Mayr (Hymenoptera, Formicidae, Myrmicinae)
Source: Zookeys. 2017 Apr 24;(670):1–109. doi: 10.3897/zookeys.670.11839 (PMC5523163; doi:10.3897/zookeys.670.11839)
Supplement: Supplementary material 1 [file zookeys-670-001-s001.pdf]

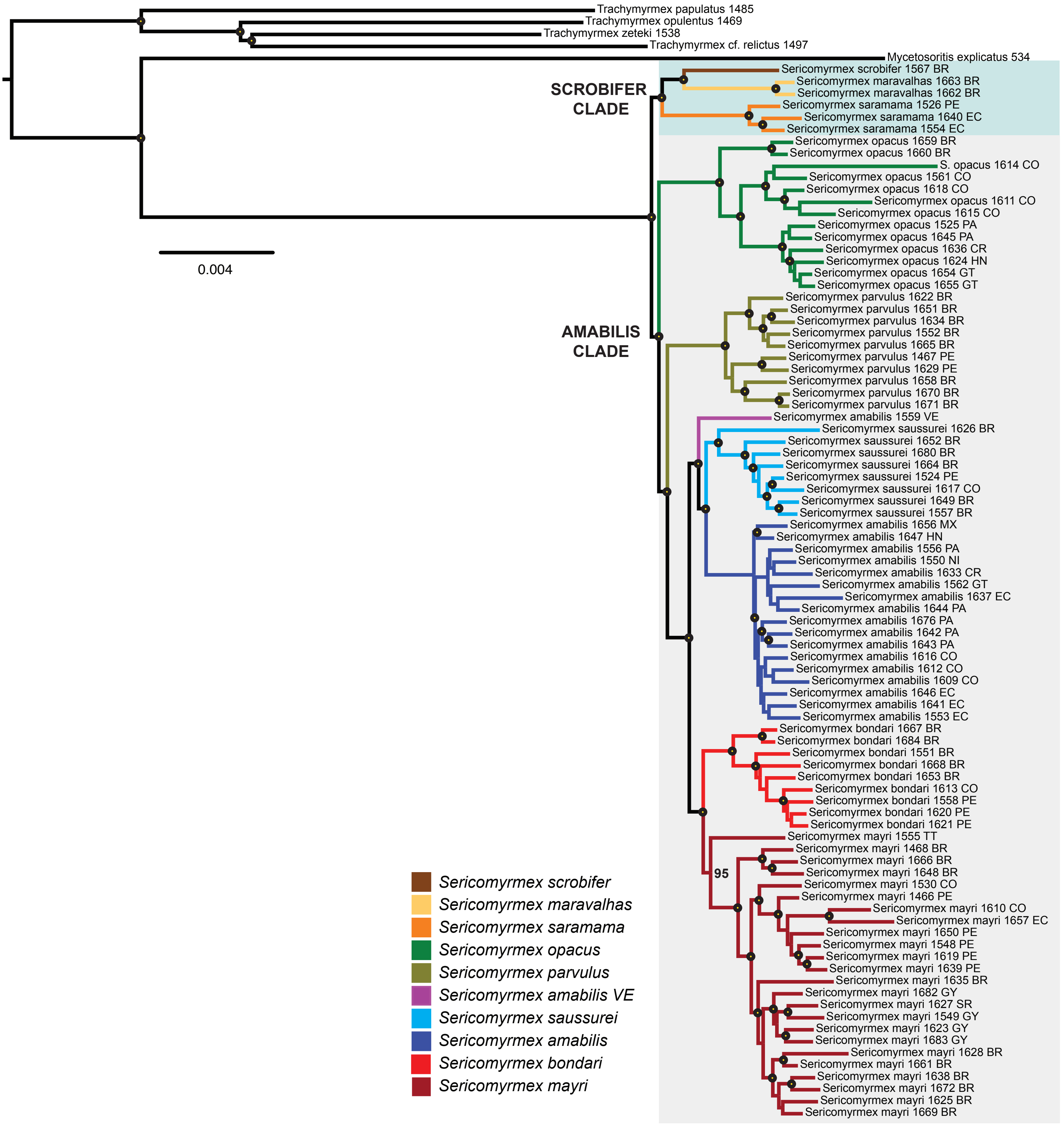

Figure S1. UCE phylogeny. The maximum-likelihood phylogeny of the 90% complete concatenated matrix containing 799 UCE loci (702,574 base pairs), adapted from Ješovnik et al., 2017. Black circles at nodes indicate 98–100 bootstrap frequencies (N=500). Taxon names include DNA extraction codes (numbers following species names) and country codes as follows: BR–Brazil, CO–Colombia, CR–Costa Rica, HN–Honduras, EC–Ecuador, GY–Guyana, GF– French Guiana, GT–Guatemala, MX–Mexico, NI–Nicaragua, PA–Panama, PE–Peru, SR–Suriname, TT– Trinidad and Tobago, VE– Venezuela.
